# Stereoselective Syntheses of Masked β-Amino Acid Containing Phthalides

**DOI:** 10.1002/hlca.202200110

**Published:** 2022-11

**Authors:** Lorenzo Serusi, Paul Zebrowski, Johannes Schörgenhumer, Antonio Massa, Mario Waser

**Affiliations:** a Institute of Organic Chemistry, Johannes Kepler University Linz, Altenbergerstr. 69, AT-4040 Linz, Austria; b Dipartimento di Chimica e Biologia “A. Zambelli”, Università degli Studi di Salerno, Via Giovanni Paolo II, IT-84084-Fisciano (SA), Italy; c Department of Chemistry, University of Zurich, Winterthurerstrasse 190, CH-8057 Zurich, Switzerland

**Keywords:** amino acids, cascade reactions, cyclization, heterocycles, organocatalysis

## Abstract

We herein report a protocol for the asymmetric aldol-initiated cascade addition of isoxazolidin-5-ones to *ortho*-cyanobenzaldehydes by using *Takemoto*’s bifunctional organocatalyst. This approach allows for the synthesis of various novel β^2,2^-amino acid-phthalide conjugates with good enantio- and diastereoselectivities in reasonable yields and the further ring-opening of these compounds to acyclic carboxylic acid derivatives was demonstrated too.

## Introduction

**Scheme 1:**
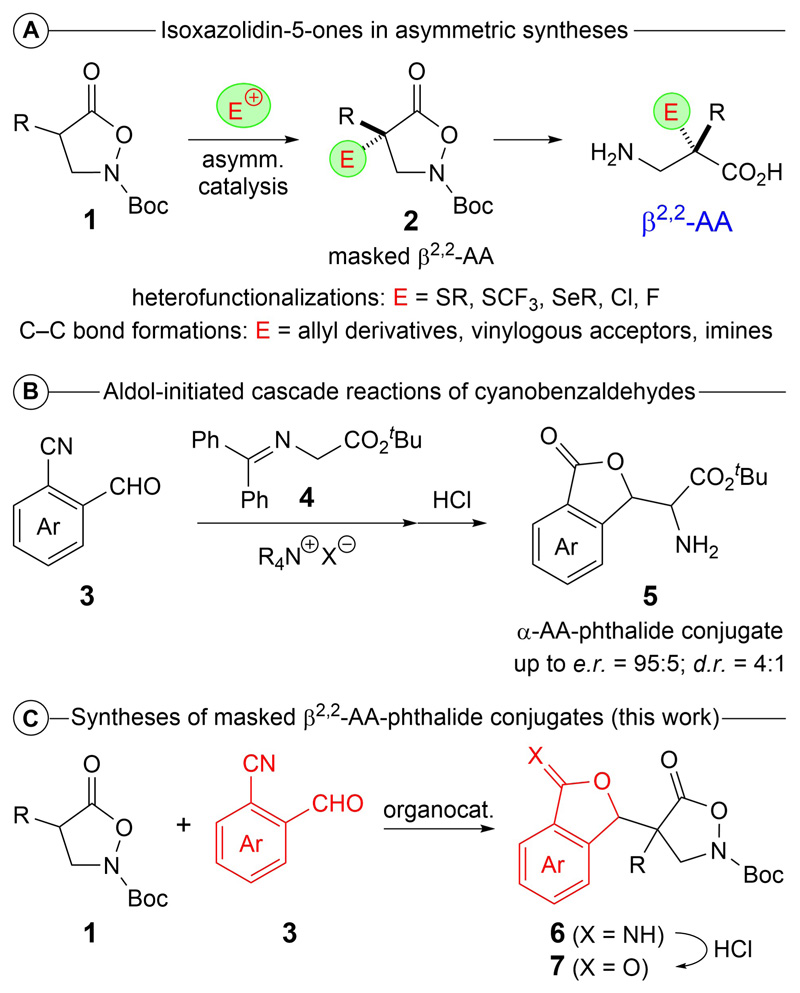
Isoxazolidin-5-ones **1** in asymmetric syntheses (A), the recently introduced aldol-initiated cascade reaction to access α-AA-phthalide conjugates **5** (B) and the herein described approach towards β-AA-phthalide conjugates **7** (C).

Isoxazolidin-5-ones **1** emerged as powerful masked β^2^-amino acid (AA) derivatives over the last years.^[1–4]^ The main value of these easily accessible heterocycles lies in the fact that they can directly be subjected to a variety of catalytic asymmetric α-functionalization reactions, which deliver the masked β^2,2^-AA derivatives **2** straightforwardly.^[5–17]^ So far, this strategy has been very successfully applied to asymmetric α-heterofunctionalizations^[6,14–17]^ as well as C C bond forming reactions^[7–13]^ either using chiral organocatalysts or asymmetric transition metal catalysis. In addition, the highly functionalized chiral compounds **2** can then be transformed into free β^2,2^-AA, peptides thereof, or other valuable chiral heterocycles, as demonstrated by several research groups over the course of the last five years (Scheme 1,A).^[5–20]^

Another class of highly valuable heterocycles are 3*H*-isobenzofuran-1-ones, or so called phthalides.^[21–23]^ A few years ago, some of us (*A. Massa*’s group) developed a chiral bifunctional ammonium salt-catalyzed aldol-initiated cascade reaction between glycine *Schiff* base **4** and *ortho*-cyanobenzaldehydes **3**, which gives access to the α-amino acid-phthalide conjugates **5** upon acidic hydrolysis of the primarily formed imidate species as well as the ketimine group (Scheme 1,B).^[24–26]^

Considering the complexity-generating potential of this cascade approach, as well as the general interest into new chiral phthalides and β-amino acid derivatives, we now became interested in addressing the asymmetric addition of isoxazolidin-5-ones **1** to cyanobenzaldehydes **3** under organocatalytic conditions. This strategy will give access to a series of highly functionalized compounds **7** (upon acidic hydrolysis of the initial reaction products **6)** and will thus provide a straightforward entry to novel β^2,2^-amino acid-phthalide conjugates (Scheme 1,C).

## Results and Discussion

**Figure 1:**
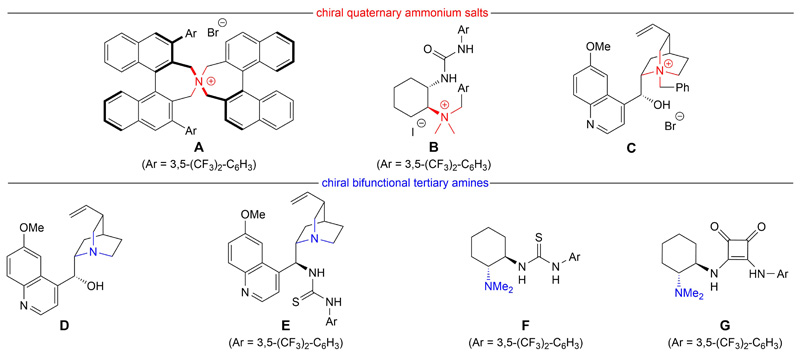
Chiral organocatalysts tested for the syntheses of products 7.

Based on our own previous experience with pronucleophiles **1**,^[11,13,15–17]^ we tested a series of easily available chiral ammonium salt catalysts (compounds **A**–**C**; Figure 1)^[25,27–29]^ as well as chiral bifunctional organobases (**D**–**G**; Figure 1)^[30–32]^ for our target reaction.

We started by investigating and optimizing the addition of the phenyl-substituted isoxazolidin-5-one **1a** to the parent cyanobenzaldehyde (**3a**; Table 1). The uncatalyzed reaction performed well in the presence of K_2_CO_3_ as a base, delivering racemic **7a** as a mixture of diastereoisomers after acidic hydrolysis of the initial reaction product **6a** (*Entry 1*). First attempts to render this reaction enantioselective were carried out with the chiral ammonium salts **A**–**C**, which we used successfully in the past for reactions of pronucleophiles **1**^[11,13,15,16]^ as well as acceptors **3**.^[24,25]^ Unfortunately however, it was not possible to obtain any reasonable levels of enantioselectivity hereby (see *Entries 2–4* for representative examples; other conditions were tested as well). Due to these unexpected results with chiral ammonium salt catalysts, we next screened the well-established bifunctional organo-bases **D**–**G**. The cinchona alkaloid quinine (**D**) was first used in the presence, as well as in the absence, of an external base (*Entries 5* and *6*). Here, we observed promising initial levels of enantioselectivity without the external base (*Entry 6*), but unfortunately, the outcome could not be improved further by using other classical cinchona alkaloids or changing the conditions. We thus next tested the well-established thiourea-containing derivative **E**^[32]^ which demonstrated the beneficial effect of the thiourea group on the enantioselectivity, albeit the conversion was found to be rather limited hereby (*Entry 7*).

Moving away from cinchona alkaloids as the chiral backbone, we then screened *Takemoto*’s cyclohexanediamine-based catalyst **F**.^[31]^ This allowed for the best enantioselectivity so far (*e.r.* = 87 : 13 for the major diastereomer) combined with a reasonable isolated yield of 51% after 24 h reaction time (*Entry 8*). Interestingly, despite the fact that notable quantities of unconverted starting materials were still observable, longer reaction times did not result in higher yields of isolated product. This is due to the fact that intermediate **6a** decomposed upon prolonged stirring in the presence of reagents and catalyst, thus leading to lower yields despite of further conversion of the starting materials upon prolonged reaction times. In order to improve the yield, we varied the stoichiometric ratios of the two reaction partners and found that an excess of **1a** allowed for better yields compared to an excess of acceptor **3a** (*Entries 9* and *10*), albeit in the latter case a slightly higher stereoselectivity was obtained. Unfortunately, longer reaction times again resulted in reduced yields, and we therefore used two equivalents of **1a** for the rest of our screening. With this information at hand, we also tested the squaramide analog **G** (*Entry 11*), but unfortunately this catalyst was found to be less suited than the thiourea derivative. We therefore carried out the final optimization with *Takemoto*’s catalyst **F**, but, as summarized in *Entries 12–16*, neither changing the solvent, nor lowering the temperature, or working under more diluted conditions allowed for any improvement anymore (compared to the results shown in *Entry 10*).

**Scheme 2:**
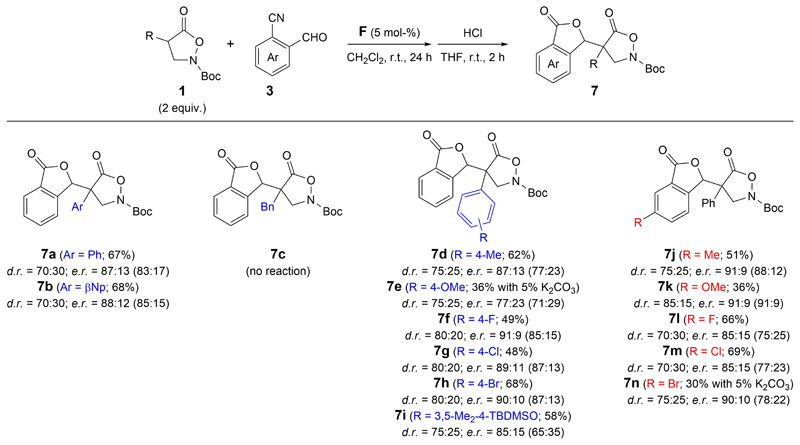
Application scope of the asymmetric cascade addition of isoxazolidin-5-ones **1** to cyanobenzaldehydes **3** (values in brackets give the *e.r.* of the minor diastereomers).

Accordingly, we used these conditions to investigate the asymmetric application scope for different isoxazolidin-5-ones **1** and cyanobenzaldehydes **3** next (Scheme 2). Unfortunately, we found that this cascade reaction is limited to α-aryl substituted pronucleophiles **1**, as the analogous α-benzyl derivative turned out to be unreactive and did not allow for any formation of product **7c**. On the other hand, a variety of different α-aryl groups was pretty well-tolerated, giving the products **7a**, **7b**, **7d**–**7i** with reasonable selectivities. However, we also observed that conversion of some starting materials was incomplete after 24 h reaction time, and especially the presence of a strongly electron donating aryl-group, as shown for product **7e**, did lead to significantly reduced reactivities. For **7e**, it was thus necessary to add a catalytic amount of an external base to allow for product formation, although the yield was still limited to 36% hereby.

Variations of the acceptor **3** were possible as well, as demonstrated for the successful formation of products **7j**–**7n**, although here as well conversion of some starting materials was somewhat limited (and especially for **7n**, the addition of external base was necessary again), showing that some structural limitations exist.

**Scheme 3:**
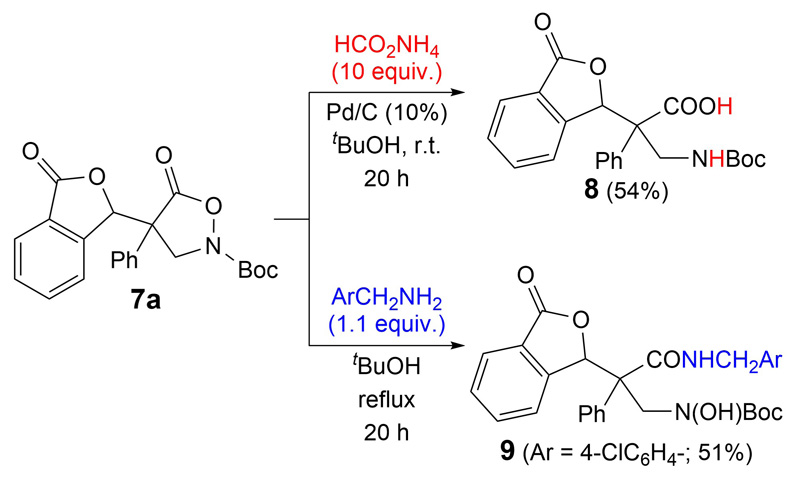
Ring opening reactions of compound 7a.

Having investigated the application scope of the asymmetric cascade reaction between compounds **1** and **3**, we finally also tested the (reductive) ring-opening of compounds **7**. As outlined in Scheme 3, the reductive N—O-cleavage could be carried out by using ammonium formate under Pd-catalysis, giving the free acid **8**. On the other hand, nucleophilic ring-opening was possible by addition of benzylamine derivatives, giving the amide **9** straightforwardly.

Unfortunately, we have not been able to obtain any suited crystals of products **7** or ring opening products **8** and **9** that would have allowed us to determine the relative, as well as the absolute, configuration of these compounds by single crystal X-ray analysis.

To at least get a plausible hint for the relative configuration of the products, we then performed DFT calculations on compound **7a**. Structure optimization revealed the *unlike*-configuration being slightly more stable than the *like*-isomer (+ 0.6 kJmol^−1^).^1^ For the geometries lowest in energy, ^13^C-NMR shifts were computed using different methods and then compared to the experimentally obtained values for both diastereomers. As simple MAE (mean absolute errors) analyses remained inconclusive and generally showed quite high deviations. Therefore, an MAE_ΔΔ*δ*_ approach as reported by the group of *Bifulco* was undertaken.^[33]^ To that end, the absolute difference ΔΔ*δ* between the calculated (Δ*δ*_calc_) and experimental differences (Δ*δ*_exp_) in chemical shifts between major and minor isomer are determined and the average of the ΔΔ*δ* values for all atoms is calculated. The resulting MAE_ΔΔ*_δ_*_ parameter is then used to determine the best comparison alignment.

In our case, for all used methods, the MAE_ΔΔ*δ*_ analysis clearly showed a better alignment of the *unlike*-configuration with the major isomer than with the minor (Table 2), which leads us to propose *unlike* as relative configuration for the major and *like* for the minor diastereomer, respectively.

## Conclusions

We succeeded in developing a protocol for the asymmetric cascade addition of isoxazolidin-5-ones **1** to *ortho*-cyanobenzaldehydes **3** by using *Takemoto*’s bifunctional catalyst **F**. This approach allows for the synthesis of the novel β^2,2^-amino acid-phthalide conjugates **7** with good enantio- and diastereoselectivities in reasonable yields after acidic hydrolysis of the primary reaction products **6**. The propensity of these compounds to undergo further ring-opening reactions was demonstrated as well, giving access to the acyclic β-AA derivatives **8** and **9** directly.

## Experimental Section

### General Information

^1^H-, ^13^C- and ^19^F-NMR spectra were recorded on a *Bruker Avance III* 300 MHz spectrometer with a broad band observe probe and a sample changer for 16 samples, on a *Bruker Avance DRX* 500 MHz spectrometer, and on a *Bruker Avance III* 700 MHz spectrometer with an *Ascend* magnet and *TCI* cryoprobe (which are property of the Austro-Czech NMR-Research Center ‘RERI-uasb’) and on a *Bruker DRX* 400 MHz spectrometer. NMR Spectra were referenced on the solvent peak and chemical shifts are given in ppm.

High resolution mass spectra were obtained using a *Thermo Fisher Scientific LTQ Orbitrap XL* with an *Ion Max API* Source. Analyses were made in the positive ionization mode if not otherwise stated. Purine (exact mass for [*M*+H]^+^ = 121.050873) and 1,2,3,4,5,6-hexakis(2,2,3,3-tetrafluoropropoxy)-1,3,5,2,4,6-triazatriphosphinane (exact mass for [*M* +H] ^+^ = 922.009798) were used for internal mass calibration.

HPLC was performed using a *Thermo Scientific Dionex Ultimate 3000* or a *Shimadzu Prominence* system with diode array detector with a *CHIRALPAK AD-H*, *OD-H*, *CHIRAL ART Amylose-SA*, *Cellulose-SB*, or *Cellulose-SZ* (250×4.6 mm, 5 μm) chiral stationary phase. Optical rotations were recorded on a *Schmidt+Haensch* polarimeter model *UniPol L1000* at 589 nm.

All chemicals were purchased from commercial suppliers and used without further purification unless otherwise stated. Isoxazolidin-5-ones **1** were synthesized as described previously.^[10,15,16]^ Dry solvents were obtained from an *MBraun-SPS-800* solvent purification system. All reactions were carried out under argon atmosphere, unless stated otherwise.

### General Cascade Cyclization Procedure to Access Products 7

The cyanobenzaldehydes **3** (1 equiv., 0.10 mmol) were added to a stirred solution of isoxazolidin-5-ones **1** (2 equiv., 0.20 mmol) and catalyst **F** (5 mol-%) in CH_2_Cl_2_ (3 mL). After stirring for 24 h at room temperature, the mixture was directly subjected to flash chromatography on silica gel with heptane/AcOEt 6 : 4 to give the intermediates **6** as mixtures of diastereoisomers. These products were then dissolved in a solution of 0.5 M HCl (1 mL) and THF (3 mL). The mixture was stirred at room temperature for 2 h and then concentrated in vacuum. The resulting residue was treated with saturated NaHCO_3_ (20 mL), extracted with CH_2_Cl_2_ (4×30 mL), and then purified by flash chromatography (heptane/AcOEt 7 : 3) to give the products **7** in the reported yields and with the reported stereoselectivities.

*Analytical Details for the Parent Product* **7a** (details for the other derivatives are given in the supplementary material): [α]_D_^23^ (*c* = 0.50, CHCl_3_) = + 47.3. ^1^H-NMR (300 MHz, CDCl_3_, 298 K): 7.77–7.75 (*m*, 1 H); 7.49–7.43 (*m*, 4 H); 7.34–7.31 (*m*, 3 H); 7.06 (*d*, *J* = 7.1, 1 H); 5.97 (*s*, 1 H); 4.70 (*d*, *J* = 12.2, 1 H); 4.17 (*d*, *J* = 12.2, 1 H); 1.27 (*s*, 9 H). ^13^C-NMR (75 MHz, CDCl_3_, 298 K): 173.0; 169.1; 155.7; 144.8; 134.1; 130.1; 129.9; 129.6; 127.4; 125.7; 124.2; 84.5; 81.0; 55.6; 54.2; 27.7. HR-ESI-MS: 418.1260 ([*M* + Na]^+^, C_22_H_21_NO_6_^+^; calc. 418.1267). HPLC (*YMC Chiral ART Amylose-SA*, eluent: hexane/^*i*^PrOH 70 : 30, 0.6 mL/min, 10 °C), retention times: *t*_minor__d1_ = 12.6 min, *t*_major__d1_ = 18.8 min, *t*_minor__d2_ = 16.4 min, *t*_major__d2_ = 21.2 min.

### *Synthesis of Compound* 8 (Reductive Cleavage of the N—O Bond)

Compound **7a** (24 mg, 0.06 mmol), HCO_2_NH_4_ (40 mg, mmol) and Pd/C (2.4 mg, 10% *w/w*) were placed in a round bottom flask and ^*t*^BuOH (2 mL) was added. The suspension was stirred vigorously at r.t. for 20 h. After completion of the reaction, the mixture was filtered through a short pad of *Celite* ® (washed with CH_2_Cl_2_). The solvent was removed *in vacuo* and then purified by flash chromatography (CHCl_3_/MeOH 9 : 1) to obtain product **8** in 54% yield (13 mg, 0.032 mmol). 1H-NMR (300 MHz, CDCl_3_, 298 K): 7.80 (*d*, *J* = 8.2, 1 H); 7.60 (*t*, *J* = 7.6, 2 H); 7.42 (*t*, *J* = 7.6, 1 H); 7.15 (*t*, *J* = 7.6, 5 H); 6.52 (*s*, 1 H); 5.88–5.73 (*m*, 2 H); 4.68 (*d*, *J* = 15.3, 1 H); 4.40 (*d*, *J* = 15.3, 1 H); 1.41 (*s*, 9 H). ^13^C-NMR (125 MHz, CDCl_3_, 298 K): 173.1; 169.2; 155.8; 144.9; 134.3; 133.9; 130.2; 130.1; 129.7; 129.5; 129.4; 129.4; 129.4; 129.1; 128.3; 128.2; 127.5; 127.5; 127.5; 126.6; 125.9; 125.5; 125.4; 124.4; 84.7; 81.2; 55.7; 54.3; 28.3; 27.8. HR-ESI-MS: 396.1450 ([*M* H]^−^, C_22_H_22_NO_6_^−^; calc. 396.1447).

### *Synthesis of Compound* 9 (Nucleophilic Ring-Opening)

Compound **7a** (24 mg, 0.06 mmol) and *p*-chlorobenzylamine (8.5 mg, 0.06 mmol) were dissolved in ^*t*^BuOH (in a pressure *Schlenk*) and stirred at 90 °C overnight. Volatiles were removed *in vacuo*, and the crude mixture was purified by column chromatography (silica gel, heptanes/AcOEt) to yield amide **9** in 51% (16 mg, 0.029 mmol). ^1^H-NMR (300 MHz, CDCl_3_, 298 K): 7.81–7.77 (*m*, 1 H); 7.62–7.55 (*m*, 2 H); 7.45–7.40 (*m*, 1 H); 7.32–7.29 (*m*, 1 H); 7.24–7.16 (*m*, 5 H); 7.07 (*d*, *J* = 8.2, 3 H); 6.55 (*s*, 1 H); 6.05 (*t*, *J* = 5.7, 1 H); 4.62 (*d*, *J* = 14.8, 1 H); 4.42 (*d*, *J* = 14.8, 2 H); 4.14 (*dd*, *J*1 = 5.1, *J*2 = 9.1, 1 H); 1.37 (*s*, 9 H). ^13^C-NMR (75 MHz, CDCl_3_, 298 K): 169.8; 156.7; 146.8; 135.9; 133.8; 133.4; 129.9; 129.5; 129.3; 128.9; 128.9; 128.8; 128.7; 128.1; 126.7; 125.3; 82.8; 59.6; 55.2; 43.4; 28.1. HR-ESI-MS: 559.1603 ([*M* + Na] ^+^, C_29_H_29_ClN_2_O_6_^+^; calc. 559.1612).

## Supplementary Material

Supporting information for this article is available on the WWW under https://doi.org/10.1002/hlca.202200110

SI

## Figures and Tables

**Table 1:** Optimization of reaction conditions.

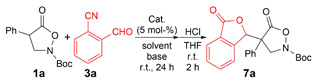							
*Entry* ^[a]^	Cat.	1a:3a	Solv.	Base	Yield [%]^[b]^	d.r.^[c]^	*e.r.* ^[d,e]^
*1*	–	1:1	CH_2_Cl_2_	K_2_CO_3_ (1 equiv.)	67	65:35	–
*2*	**A**	1:1	CH_2_Cl_2_	K_2_CO_3_ (1 equiv.)	44	80:20	rac.
*3*	**B**	1:1	CH_2_Cl_2_	K_2_CO_3_ (1 equiv.)	59	65:35	rac.
*4*	**C**	1:1	CH_2_Cl_2_	K_2_CO_3_ (1 equiv.)	26	85:15	55:45 (70:30)
*5*	**D**	1:1	CH_2_Cl_2_	K_2_CO_3_ (1 equiv.)	38	60:40	57:43 (50:50)
*6*	**D**	1:1	CH_2_Cl_2_	–	54	70:30	75:25 (72:28)
*7*	**E**	1:1	CH_2_Cl_2_	–	18	90:10	78:22 (77:23)
*8*	**F**	1:1	CH_2_Cl_2_	–	51	70:30	87:13 (77:23)
*9*	**F**	1:2	CH_2_Cl_2_	–	27	80:20	89:11 (77:23)
***10***	**F**	**2:1**	**CH_2_Cl_2_**	–	**67**	**70:30**	**87:13 (83:17)**
*11*	**G**	2:1	CH_2_Cl_2_	–	31	65:35	76:24 (69:31)
*12*	**F**	2:1	THF	–	36	70:30	80:20 (79:21)
*13*	**F**	2:1	toluene	–	51	65:35	68:32 (80:20)
*14*	**F**	2:1	CH_2_Cl_2_ (0 °C)	–	59	70:30	85:15 (75:25)
*15*	**F**	2:1	CH_2_Cl_2_ (−20 °C)	–	59	70:30	85:15 (77:23)
*16*	**F**	2:1	CH_2_Cl_2_ (0.03 M)	–	34	75:25	81:19(80:20)

[a] All reactions were run for 24 h at r.t. using 0.1 mmol of the limiting reagent in the indicated solvent (0.06 m with respect to **3a**) with 5 mol-% of the given catalyst and base unless otherwise stated.

[b] Yields of the combined isolated diastereomers.

[c] Determined by ^1^H-NMR of the crude product (the *unlike* diastereomer was assigned as the major diastereomer as discussed in the final section of this contribution).

[d] Determined by HPLC using a chiral stationary phase.

[e] Values in brackets give the *e.r.* of the minor diastereomer.

**Table 2:** Results of the MAE_ΔΔ*δ*_ assessment for the structures lowest in energy. NMR calculations were performed on optimized structures (IEFPCM(CHCl_3_)-B3LYP/6-311 + G(2d,p)). All methods below included an implicit description of CHCl_3_ (IEFPCM). *(R,S)* was used as model for *unlike, (R,R)* for *like* configuration, respectively. The column *Selected atoms* features the MAE_ΔΔ*δ*_-calculation discarding atoms that showed highly anisotropic shifts due to the non-dynamic nature of the GIAO-NMR calculation (*e.g.* the three Me groups). The calculated shifts for these atoms have been averaged in the section *All atoms.*

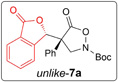				
DFT method	All atoms	MAE_ΔΔ*δ*_ (*R,S =* min)	Selected atoms	MAE_ΔΔ*δ*_ (*R,S* = min)
	MAE_ΔΔ*δ*_ (*R,S* = maj)		MAE_ΔΔ*δ*_ (*R,S* = maj)	
B3LYP/6-311 + G(2d,p)	1.06	1.25	1.17	1.46
MPW1PW91/6-311 + G(2d,p)	0.99	1.23	1.16	1.44
PBE0/6-311 + G(2d,p)	1.00	1.23	1.16	1.44
B3LYP/aug-cc-pvdz	0.99	1.11	1.16	1.34

## Data Availability

The data that support the findings of this study are available from the corresponding author upon reasonable request.
