# Supplementary material for: Stereoselective Syntheses of Masked β-Amino Acid Containing Phthalides
Source: Helv Chim Acta. Author manuscript; Available in PMC 2023 Feb 23. (PMC7614226; doi:10.1002/hlca.202200110)
Supplement: SI [file EMS167289-supplement-SI.pdf]

HELVETICA
chimica acta

Supporting Information

Stereoselective Syntheses of Masked β-Amino Acid Containing Phthalides

Lorenzo Serusi, Paul Zebrowski, Johannes Schörgenhumer, Antonio Massa, and Mario Waser*© 2022 The Authors. Helvetica Chimica Acta published by Wiley-VHCA AG. This is an open access article under the terms of the Creative Commons Attribution License, which permits use, distribution and reproduction in any medium, provided the original work is properly cited.

Stereoselective syntheses of masked β-amino acid containing phthalides

Lorenzo Serusi,[a,b] Paul Zebrowski,[a] Johannes Schörgenhumer,[c] Antonio Massa,[b] and Mario Waser*,[a]

[a] Institute of Organic Chemistry, Johannes Kepler University Linz, Altenbergerstr. 69, 4040 Linz, Austria, mario.waser@jku.at

[b] Dipartimento di Chimica e Biologia "A. Zambelli", Università degli Studi di Salerno, Via Giovanni Paolo II, 84084-Fisciano (SA), Italy

[c] Department of Chemistry, University of Zurich, Winterthurerstrasse 190, 8057 Zurich, Switzerland

List of Contents

1. General Information 2

2. Synthesis and Analytic Details of Targets 7, 8 and 9 3

2.1 General Procedure for the Cascade Reaction Forming Products 7 3

2.2 Further Transformations and Products 10

3. Computational Details 12

4. Copies of Product NMR Spectra and HPLC Chromatograms. 21

1. General Information

^{1}H-, ^{13}C- and ^{19}F-NMR spectra were recorded on a Bruker Avance III 300 MHz spectrometer with a broad band observe probe and a sample changer for 16 samples, on a Bruker Avance DRX 500 MHz spectrometer, and on a Bruker Avance III 700 MHz spectrometer spectrometer with an Ascend magnet and TCI cryoprobe (which are property of the Austro-Czech NMR-Research Center "RERI-uasb") and on a Bruker DRX 400 MHz spectrometer. NMR spectra were referenced on the solvent peak and chemical shifts are given in ppm.

High resolution mass spectra were obtained using a Thermo Fisher Scientific LTQ Orbitrap XL with an Ion Max API Source. Analyses were made in the positive ionization mode if not otherwise stated. Purine (exact mass for $[M+H]^+$ = 121.050873) and 1,2,3,4,5,6-hexakis(2,2,3,3-tetrafluoropropoxy)-1,3,5,2,4,6-triazatriphosphinane (exact mass for $[M+H]^+$ = 922.009798) were used for internal mass calibration.

HPLC was performed using a Thermo Scientific Dionex Ultimate 3000 or a Shimadzu Prominence system with diode array detector with a CHIRALPAK AD-H, OD-H, CHIRAL ART Amylose-SA, Cellulose-SB, or Cellulose-SZ (250 × 4.6 mm, 5 µm) chiral stationary phase. Optical rotations were recorded on a Schmidt + Haensch Polarimeter Model UniPol L1000 at 589 nm.

All chemicals were purchased from commercial suppliers and used without further purification unless otherwise stated. Isoxazolidin-5-ones **1**[1] were synthesized as described previously. Dry solvents were obtained from an MBraun-SPS-800 solvent purification system. All reactions were carried out under argon atmosphere, unless stated otherwise.

[1] (a) M. Nascimento de Oliveira, S. Arseniyadis, J. Cossy, *Chem. Eur. J.* **2018,** *24,* 4810–4814; (b) T. Cadart, C. Berthonneau, V. Levacher, S. Perrio, J.-F. Brière, *Chem. Eur. J.* **2016,** *22,* 15261–15264.

2. Synthesis and Analytic Details of Targets 7, 8 and 9

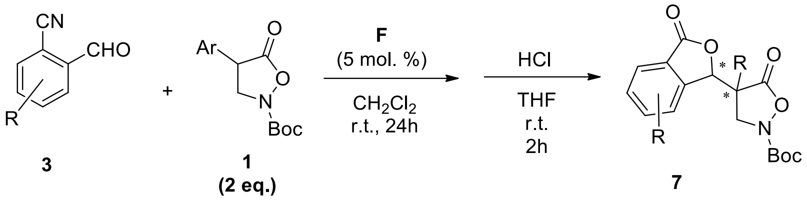

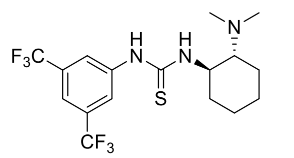

F

2.1 General Procedure for the Cascade Reaction Forming Products 7

In a round bottom-flask, 2-cyanobenzaldehydes **3** (1 eq., 0.10 mmol) were added at room temperature to a stirred solution of isoxazolidin-5-ones **1** (2 eq., 0.20 mmol) and catalyst **F** (5% mol) in DCM (3mL). After stirring for 24 h, the mixture was purified directly by flash chromatography on silica gel with heptane:ethyl acetate = 6:4 to give the intermediates **6** as mixtures of diasteroisomers. These products were then dissolved in a solution of 0.5 M HCl (1 mL) and THF (3 mL). The mixture was stirred at room temperature for 2 h and then concentrated in vacuum. The resulting residue was treated with saturated $NaHCO_3$ (20 mL), extracted with CH_2Cl_2 (4x30 mL), and then purified by flash chromatography (heptane:EtOAc= 7:3).

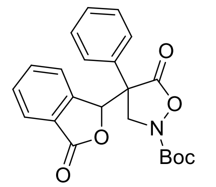

7a

Following the general procedure using 0.1 mmol of the respective 2-cyanobenzaldehyde compound **7a** was obtained in 67% yield with *e.r.= 87:13* (83:17) *d.r.:* 70/30 as an oily residue (26 mg, 0.067 mmol).

$[\alpha]_D^{23}$ (c = 0.50, $CHCl_3$) = +47.3°.^{1}H NMR (300 MHz, δ, $CDCl_3$, 298 K): 7.77-7.75 (m, 1H), 7.49-7.43 (m, 4H), 7.34-7.31 (m, 3H), 7.06 (d, *J* = 7.1 Hz, 1H), 5.97 (s, 1H), 4.70 (d, *J* = 12.2, 1H), 4.17 (d, *J* = 12.2, 1H), 1.27 (s, 9H). ^{13}C NMR (75 MHz, δ, $CDCl_3$, 298 K): 173.0, 169.1, 155.7, 144.8, 134.1, 130.1, 129.9, 129.6, 127.4, 125.7, 124.2, 84.5, 81.0, 55.6, 54.2, 27.7. HRMS (ESI): calcd *m/z* for $C_{22}H_{21}NO_6$: 418.1267 $[M+Na]^+$; found: 418.1260. HPLC (YMC Chiral ART Amylose-SA, eluent: hexane:*i*-PrOH = 70:30, 0.6 mL/min, 10 °C), retention times: $t_{minor\ d1}$=12.6 min, $t_{major\ d1}$= 18.8 min, $t_{minor\ d2}$=16.4 min, $t_{major\ d2}$= 21.2 min.

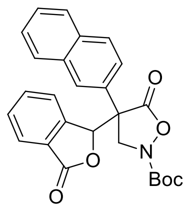

7b

Following the general procedure using 0.1 mmol of the respective 2-cyanobenzaldehyde compound **7b** was obtained in 68% yield with *e.r.= 88:12* (85:15) *d.r.:* 70/30 as oily residue (30 mg, 0.068 mmol).

$[\alpha]_D^{23}$ (c = 1.00, $CHCl_3$) = +48.9°. ^{1}H NMR (300 MHz, δ, $CDCl_3$, 298 K): 7.85-7.67 (m, 4H), 7.52-7.43 (m, 4H), 7.36-7.33 (m, 2H), 6.96-6.93 (m, 1H), 6.03 (s, 1H), 4.75 (d, *J* = 12.2 Hz, 1H), 4.15 (d, *J* = 12.2 Hz, 1H), 1.11 (s, 9H). ^{13}C NMR (75 MHz, δ, $CDCl_3$, 298 K): 173.0, 169.1, 155.7, 144.8, 134.2, 134.2, 133.2, 133.2, 133.0, 132.8, 130.2, 130.1, 129.7, 129.3, 128.4, 128.4, 127.8, 127.7, 127.6, 127.5, 127.4, 127.3, 127.0, 126.7, 126.4, 126.0, 125.8, 124.2, 124.0, 123.9, 123.4, 84.5, 81.0, 55.8, 54.1, 27.6. HRMS (ESI): calcd *m/z* for $C_{26}H_{23}NO_6$: 468.1423 $[M+Na]^+$; found: 468.1416. HPLC (YMC Chiral ART Amylose-SA, eluent: hexane:*i*-PrOH = 70:30, 0.6 mL/min, 10 °C), retention times: $t_{minor\ d1}$=15.1 min, $t_{major\ d1}$= 23.7 min, $t_{minor\ d2}$=19.9 min, $t_{major\ d2}$= 36.0 min.

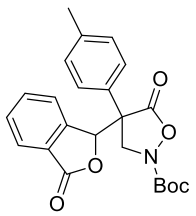

7d

Following the general procedure using 0.1 mmol of the respective 2-cyanobenzaldehyde compound **7d** was obtained in 62% yield with *e.r.= 87:13* (77:23) *d.r.:* 75/25 as an oily residue (25 mg, 0.062 mmol).

$[\alpha]_D^{23}$ (c = 1.00, $CHCl_3$) = +36.3°.^{1}H NMR (300 MHz, δ, $CDCl_3$, 298 K): 7.79-7.76 (m, 1H), 7.49-7.45 (m, 2H), 7.35.7.32 (m, 3H), 7.14 (d, *J* = 7.8 Hz, 2H), 5.96 (s, 1H), 4.64 (d, *J* = 12.2 Hz, 1H), 4.09 (d, *J* = 12.2 Hz, 1H), 2.30 (s, 3H), 1.29 (s, 9H). ^{13}C NMR (75 MHz, δ, $CDCl_3$, 298 K): 173.0, 169.1, 155.7, 144.9, 144.7, 139.8, 139.7, 134.1, 130.2, 129.9, 127.6, 127.2, 126.8, 126.7, 126.4, 125.9, 125.7, 124.2, 123.4, 84.4, 81.1, 57.5, 56.2, 55.3, 53.8, 27.7, 27.6, 21.0. HRMS (ESI): calcd *m/z* for $C_{23}H_{23}NO_6$: 432.1423 $[M+Na]^+$; found: 432.1417. HPLC (YMC Chiral ART Amylose-SA, eluent: hexane:*i*-PrOH = 70:30, 0.6 mL/min, 10 °C), retention times: $t_{minor\ d1}$=12.3 min, $t_{major\ d1}$= 18.5 min, $t_{minor\ d2}$=14.8 min, $t_{major\ d2}$= 23.5 min.

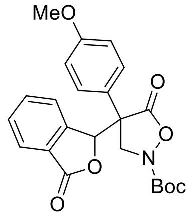

7e

Following the general procedure with additional 5 mol% K_2CO_3 using 0.1 mmol of the respective 2-cyanobenzaldehyde compound **7e** was obtained in 36% yield with *e.r.= 77:23* (71:29) *d.r.:* 75/25 as an oily residue (15 mg, 0.035 mmol).

$[\alpha]_D^{23}$ (c = 0.30, $CHCl_3$) = +30.6°. ^{1}H NMR (300 MHz, δ, $CDCl_3$, 298 K): 7.81-7.78 (m, 1H), 7.52-7.47 (m, 2H), 7.40-7.37 (m, 2H), 7.07-7.02 (m, 1H), 6.86 (d, *J* = 9.3 Hz, 2H), 5.96 (s, 1H), 4.64 (d, *J* = 12.1 Hz, 1H), 4.11 (d, *J* = 12.1 Hz, 1H), 3.78 (s, 3H), 1.32 (s, 9H). ^{13}C NMR (75 MHz, δ, $CDCl_3$, 298 K): 173.1, 169.1, 144.9, 134.1, 130.0, 129.0, 128.7, 126.5, 125.9, 124.7, 124.2, 123.4, 121.5, 114.8, 114.5, 84.4, 81.1, 55.4, 54.9, 53.9, 27.7. HRMS (ESI): calcd *m/z* for $C_{23}H_{23}NO_7$: 448.1372 $[M+Na]^+$; found: 448.1367. HPLC (YMC Chiral ART Amylose-SA, eluent: hexane:*i*-PrOH = 70:30, 1 mL/min, 10 °C), retention times: $t_{minor\ d1}$=9.5 min, $t_{major\ d1}$= 16.7 min, $t_{minor\ d2}$=11.7 min, $t_{major\ d2}$= 21.5 min.

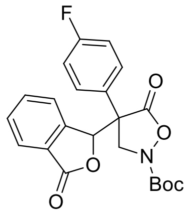

7f

Following the general procedure using 0.1 mmol of the respective 2-cyanobenzaldehyde compound **7f** was obtained in 49% yield with *e.r.= 91:9* (85:15) *d.r.:* 80/20 as an oily residue (20 mg, 0.049 mmol).

$[\alpha]_D^{23}$ (c = 1.00, $CHCl_3$) = +58.4°. ^{1}H NMR (300 MHz, δ, $CDCl_3$, 298 K): 7.76 (d, *J* = 7.0 Hz, 1H), 7.56-7.39 (m, 5H), 7.23-7.18 (m, 1H), 7.03-6.97 (m, 2H), 5.93 (s, 1H), 4.68 (d, *J* = 12.2 Hz, 1H), 4.25 (d, *J* = 12.2 Hz, 1H), 1.30 (s, 9H). ^{13}C NMR (75 MHz, δ, $CDCl_3$, 298 K): 172.8, 168.9, 155.6, 144.6, 134.5, 134.2, 130.4, 130.2, 129.8, 129.7, 129.5, 129.4, 126.4, 125.8, 125.8, 124.2, 123.3, 116.7, 116.4, 116.1, 84.7, 80.8, 56.7, 56.0, 55.2, 55.0, 27.7. HRMS (ESI): calcd *m/z* for $C_{22}H_{20}FNO_6$: 436.1172 $[M+Na]^+$; found: 436.1169. HPLC (YMC Chiral ART Amylose-SA, eluent: hexane:*i*-PrOH = 70:30, 0.6 mL/min, 10 °C), retention times: $t_{minor\ d1}$=14.0 min, $t_{major\ d1}$= 19.5 min, $t_{minor\ d2}$=16.2 min, $t_{major\ d2}$= 28.3 min.

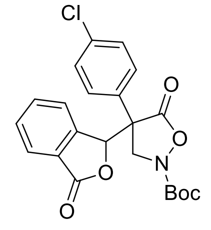

7g

Following the general procedure using 0.1 mmol of the respective 2-cyanobenzaldehyde compound **7g** was obtained in 48% yield with *e.r.= 89:11* (87:13*) d.r.:* 80/20 as an oily residue (21 mg, 0.048 mmol).

$[\alpha]_D^{23}$ (c = 1.00, $CHCl_3$) = +28.2°. ^{1}H NMR (300 MHz, δ, $CDCl_3$, 298 K): 7.78 (d, *J* = 7.3 Hz, 1H), 7.58-7.49 (m, 3H), 7.39-7.28 (m, 4H), 5.94 (s, 1H), 4.67 (d, *J* = 12.2 Hz, 1H), 4.26 (d, *J* = 12.2 Hz, 1H), 1.32 (s, 9H). ^{13}C NMR (75 MHz, δ, $CDCl_3$, 298 K): 172.6, 168.9, 155.6, 144.5, 135.9, 134.3, 130.3, 129.7, 129.3, 129.1, 128.9, 128.6, 126.4, 125.9, 124.2, 84.8, 80.7, 55.3, 27.7. HRMS (ESI): calcd *m/z* for $C_{22}H_{20}ClNO_6$: 452.0877 $[M+Na]^+$; found: 452.0873. HPLC (YMC Chiral ART Amylose-SA, eluent: hexane:*i*-PrOH = 70:30, 0.6 mL/min, 10 °C), retention times: $t_{minor\ d1}$=14.8 min, $t_{major\ d1}$= 20.3 min, $t_{minor\ d2}$=17.5 min, $t_{major\ d2}$= 30.8 min.

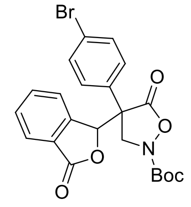

7h

Following the general procedure using 0.1 mmol of the respective 2-cyanobenzaldehyde compound **7h** was obtained in 68% yield with *e.r.= 90:10* (87:13) *d.r.:* 80/20 as an oily residue (32 mg, 0.067 mmol).

$[\alpha]_D^{23}$ (c = 1.00, $CHCl_3$) = +28.1°.^{1}H NMR (500 MHz, δ, $CDCl_3$, 298 K): 7.77 (d, *J* = 7.3 Hz, 1H), 7.55-7.42 (m, 4H), 7.30 (d, *J* = 8.2 Hz, 2H), 7.24 (d, *J* = 7.3 Hz, 1H), 5.93 (s, 1H), 4.65 (d, *J* = 12.3 Hz, 1H), 4.24 (d, *J* = 12.3 Hz, 1H), 1.31 (s, 9H). ^{13}C NMR (125 MHz, δ, $CDCl_3$, 298 K): 172.9, 169.2, 155.9, 144.8, 134.9, 134.7, 133.0, 132.7, 130.8, 130.6, 129.7, 129.5, 126.7, 126.5, 126.2, 124.5, 124.4, 123.6, 85.2, 81.5, 80.9, 56.8, 55.7, 55.1, 28.0. HRMS (ESI): calcd *m/z* for $C_{22}H_{20}BrNO_6$: 496.0372 $[M+Na]^+$; found: 496.0365. HPLC (YMC Chiral ART Amylose-SA, eluent: hexane:*i*-PrOH = 70:30, 0.6 mL/min, 10 °C), retention times: $t_{minor\ d1}$=11.9 min, $t_{major\ d1}$= 16.3 min, $t_{minor\ d2}$=14.6 min, $t_{major\ d2}$= 25.3 min.

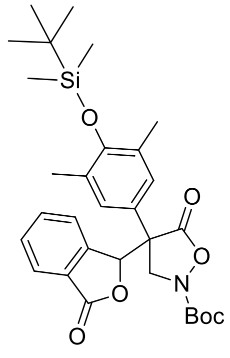

7i

Following the general procedure using 0.1 mmol of the respective 2-cyanobenzaldehyde compound **7i** was obtained in 58% yield with *e.r.= 85:15* (65:35) *d.r.:* 75/25 as an oily residue (32 mg, 0.058 mmol).

$[\alpha]_D^{23}$ (c = 1.00, $CHCl_3$) = +123.4°. ^{1}H NMR (300 MHz, δ, $CDCl_3$, 298 K): 7.79-7.76 (m, 1H), 7.46-7.43 (m, 2H), 7.03 (m, 2H), 6.88-6.86 (m, 1H), 5.94 (s, 1H), 4.57 (d, *J* = 12.2 Hz, 1H), 3.98 (d, *J* = 12.2 Hz, 1H), 2.14 (s, 6H), 1.30 (s, 9H), 0.97 (s, 9H), 0.14 (s, 3H), 0.12 (s, 3H). ^{13}C NMR (350 MHz, δ, $CDCl_3$, 298 K): 173.3, 169.3, 156.0, 153.5, 145.2, 134.0, 130.1, 129.8, 128.0, 127.8, 126.6, 125.8, 124.4, 122.0, 84.3, 81.2, 54.9, 53.1, 32.0, 29.2, 27.9, 26.2, 22.8, 18.9, 18.1, 14.3. HRMS (ESI): calcd *m/z* for $C_{30}H_{39}NO_7Si$: 576.2393 $[M+Na]^+$; found: 576.2388. HPLC (YMC Chiral ART Amylose-SA, eluent: hexane:*i*-PrOH = 90:10, 0.6 mL/min, 10 °C), retention times: $t_{minor\ d1}$=15.7 min, $t_{major\ d1}$= 21.7 min, $t_{minor\ d2}$=19.2 min, $t_{major\ d2}$= 33.8 min.

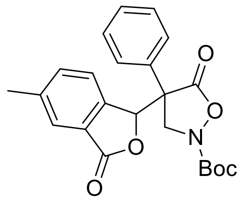

7j

Following the general procedure using 0.1 mmol of the respective 2-cyanobenzaldehyde compound **7j** was obtained in 51% yield with *e.r.= 91:9* (88:12) *d.r.:* 75/25 as an oily residue (21 mg, 0.051 mmol).

$[\alpha]_D^{23}$ (c = 0.80, $CHCl_3$) = +39.5°. ^{1}H NMR (300 MHz, δ, $CDCl_3$, 298 K): 7.56 (s, 1H), 7.49-7.44 (m, 3H), 7.35-7.33 (m, 3H), 7.85 (d, *J* = 7.8 Hz, 1H), 5.93 (s, 1H), 4.67 (d, *J* = 11.9 Hz, 1H), 4.12 (d, *J* = 11.9 Hz, 1H), 2.37 (s, 3H), 1.28 (s, 9H). ^{13}C NMR (75 MHz, δ, $CDCl_3$, 298 K): 173.0. 169.2, 155.7, 142.1, 140.5, 135.4, 135.3, 130.1, 129.6, 129.5, 129.5, 129.2, 127.7, 127.4, 126.6, 126.0, 125.7, 123.8, 123.0, 84.5, 81.0, 55.6, 53.8, 27.7, 21.2. HRMS (ESI): calcd *m/z* for $C_{23}H_{23}NO_6$: 432.1423 $[M+Na]^+$; found: 432.1418. HPLC (YMC Chiral ART Amylose-SA, eluent: hexane:*i*-PrOH = 70:30, 0.6 mL/min, 10 °C), retention times: $t_{minor\ d1}$=11.8 min, $t_{major\ d1}$= 16.9 min, $t_{minor\ d2}$=15.0 min, $t_{major\ d2}$= 18.7 min

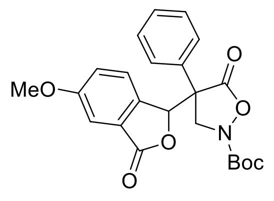

7k

Following the general procedure using 0.1 mmol of the respective 2-cyanobenzaldehyde compound **7k** was obtained in 36% yield with *e.r.= 91:9* (91:9) *d.r.:* 85/15 as an oily residue (15 mg, 0.036 mmol).

$[\alpha]_D^{23}$ (c = 0.30, $CHCl_3$) = +138.0°. ^{1}H NMR (300 MHz, δ, $CDCl_3$, 298 K): 7.49-7.45 (m, 3H), 7.35 (t, *J* = 3.2 Hz, 3H), 7.19 (d, *J = 2.5 Hz*, 1H), 6.90 (d, *J* = 8.5 Hz, 1H), 5.92 (s, 1H), 4.70 (d, *J* = 12.4 Hz, 1H), 4.15 (d, *J* = 12.4 Hz, 1H), 3.81 (s, 3H), 1.29 (s, 9H). ^{13}C NMR (75 MHz, δ, $CDCl_3$, 298 K): 173.0, 169.1, 161.2, 155.7, 137.0, 136.7, 133.3, 130.1, 129.6, 129.5, 129.2, 128.2, 128.0, 127.9, 127.6, 127.4, 125.1, 124.2, 122.9, 122.9, 107.5, 84.5, 81.0, 55.8, 55.7, 55.6, 53.9, 27.7. HRMS (ESI): calcd *m/z* for $C_{23}H_{23}NO_7$: 448.1372 $[M+Na]^+$; found: 448.1369. HPLC (YMC Chiral ART Amylose-SA, eluent: hexane:*i*-PrOH = 70:30, 0.6 mL/min, 10 °C), retention times: $t_{minor\ d1}$=13.6 min, $t_{major\ d1}$= 19.2 min, $t_{minor\ d2}$=15.9 min, $t_{major\ d2}$= 22.2 min.

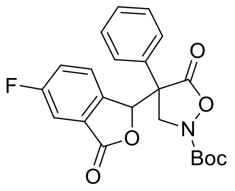

7l

Following the general procedure using 0.1 mmol of the respective 2-cyanobenzaldehyde compound **7l** was obtained in 66% yield with *e.r.= 85:15* (75:25) *d.r.:* 70/30 as an oily residue (27 mg, 0.065 mmol).

$[\alpha]_D^{23}$ (c = 1.00, $CHCl_3$) = +71.7°.^{1}H NMR (300 MHz, δ, $CDCl_3$, 298 K): 7.51-7.49 (m, 2H), 7.42-7.37 (m, 3H), 7.33-7.32 (m, 3H), 7.24-7.21 (m, 1H), 5.94 (s, 1H), 4.79 (d, *J* = 12.3 Hz, 1H), 4.26 (d, *J* = 12.3 Hz, 1H), 1.27 (s, 9H). ^{13}C NMR (75 MHz, δ, $CDCl_3$, 298 K): 173.1, 167.8 (*J* = 3.9 Hz), 165.1, 161.8, 155.6, 140.4 (*J* = 2.3 Hz), 129.7 (*J* = 9.2 Hz), 129.3, 128.6 (*J* = 9.2 Hz), 127.3, 126.3 (*J* = 8.2 Hz), 122.0 *(J* = 23.3 Hz*)*, 112.0 (*J* = 23.3 Hz), 84.6, 81.0, 55.7, 55.2, 27.6. HRMS (ESI): calcd *m/z* for $C_{22}H_{20}FNO_6$: 436.1172 $[M+Na]^+$; found: 436.1169. HPLC (YMC Chiral ART Amylose-SA, eluent: hexane:*i*-PrOH = 90:10, 1 mL/min, 10 °C), retention times: $t_{minor\ d1}$=13.8 min, $t_{major\ d1}$= 25.5 min, $t_{minor\ d2}$=18.2 min, $t_{major\ d2}$= 33.6 min

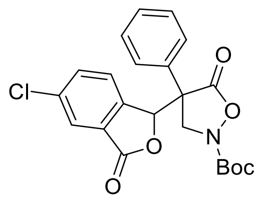

7m

Following the general procedure using 0.1 mmol of the respective 2-cyanobenzaldehyde compound **7m** was obtained in 69% yield with *e.r.= 85:15* (77:23) *d.r.:* 70/30 as an oily residue (29 mg, 0.069 mmol).

$[\alpha]_D^{23}$ (c = 1.00, $CHCl_3$) = +31.8°.^{1}H NMR (300 MHz, δ, $CDCl_3$, 298 K): 7.70 (d, *J* = 1.7 Hz, 1H), 7.49-7.39 (m, 3H), 7.34-7.32 (m, 3H), 7.19 (d, *J* = 8.1 Hz, 1H), 5.93 (s, 1H), 4.79 (d, *J* = 12.5 Hz, 1H), 4.26 (d, *J* = 12.5 Hz, 1H), 1.27 (s, 9H). ^{13}C NMR (75 MHz, δ, $CDCl_3$, 298 K): 173.1, 167.6, 155.6, 143.0, 136.5, 134.4, 129.7, 129.7, 129.3, 128.2, 127.3, 125.7, 125.5, 84.6, 81.0, 55.6, 55.2, 27.6. HRMS (ESI): calcd *m/z* for $C_{22}H_{20}ClNO_6$: 452.0877 $[M+Na]^+$; found: 452.0874. HPLC (YMC Chiral ART Amylose-SA, eluent: hexane:*i*-PrOH = 90:10, 1 mL/min, 10 °C), retention times: $t_{minor\ d1}$=13.6 min, $t_{major\ d1}$= 25.3 min, $t_{minor\ d2}$=17.1 min, $t_{major\ d2}$= 32.1 min.

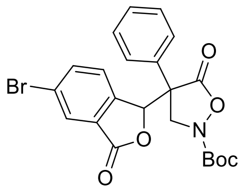

7n

Following the general procedure with additional 5 mol K_2CO_3 using 0.1 mmol of the respective 2-cyanobenzaldehyde compound **7n** was obtained in 30% yield with *e.r.= 90:10* (78:22) *d.r.:* 75/25 as an oily residue (14 mg, 0.03 mmol).

$[\alpha]_D^{23}$ (c = 0.80, $CHCl_3$) = +41.6°.^{1}H NMR (400 MHz, δ, $CDCl_3$, 298 K): 7.86 (d, *J* = 1.7 Hz, 1H), 7.61 (dd, J^1 = 1.7 Hz, J^2 = 6.4 Hz, 1H), 7.39-7.38 (m, 2H), 7.33-7.31 (m, 3H), 7.11 (d, *J* = 8.1 Hz, 1H), 5.90 (s, 1H), 4.78 (d, *J* = 12.5 Hz, 1H), 4.24 (d, *J* = 12.5 Hz, 1H), 1.26 (s, 9H). ^{13}C NMR (100 MHz, δ, $CDCl_3$, 298 K): 173.2, 167.6, 155.7, 143.7, 137.4, 129.9, 129.8, 129.5, 128.7, 128.6, 127.5, 126.1, 124.4, 84.8, 81.2, 55.7, 55.3, 27.8. HRMS (ESI): calcd *m/z* for $C_{22}H_{20}BrNO_6$: 496.0372 $[M+Na]^+$; found: 496.0366. HPLC (YMC Chiral ART Amylose-SA, eluent: hexane:*i*-PrOH = 90:10, 1 mL/min, 10 °C), retention times: $t_{minor\ d1}$=14.5 min, $t_{major\ d1}$= 27.5 min, $t_{minor\ d2}$=18.5 min, $t_{major\ d2}$= 35.4 min.

2.2 Further Transformations and Products

2.2.1. Reductive Cleavage of the Isoxazolidin-5-one N-O Bond.

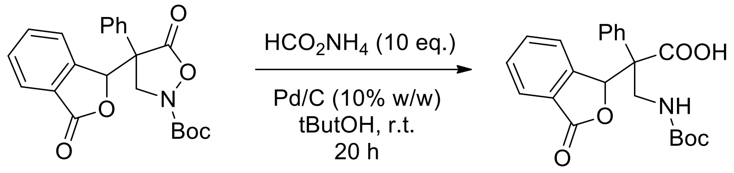

7a **8**

Under an Argon atmosphere, product **7a** (0.06 mmol), HCO_2NH_4 (0.6 mmol) and Pd/C (10% w/w) were placed in a round bottom flask and tBuOH (2 mL) was added. The suspension was stirred vigorously at r.t. for 20 h. After completion of the reaction, the mixture was filtered through a short pad of Celite® (washed with DCM). The solvent was removed in vacuo and then purified by flash chromatography (Chloroform:Methanol= 9:1) to obtain product **8** in 54% isolated yield (13 mg, 0.032 mmol).

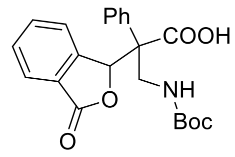

8

^{1}H NMR (300 MHz, δ, $CDCl_3$, 298 K): 7.80 (d, *J* = 8.2 Hz, 1H), 7.60 (t, *J* = 7.6 Hz, 2H), 7.42 (t, *J*= 7.6 Hz, 1H), 7.15 (t, *J* = 7.6 Hz, 5H), 6.52 (s, 1H), 5.88-5.73 (m, 2H), 4.68 (d, *J*= 15.3 Hz, 1H), 4.40 (d, *J*= 15.3 Hz, 1H), 1.41 (s, 9H). ^{13}C NMR (125 MHz, δ, $CDCl_3$, 298 K): 173.1, 169.2, 155.8, 144.9, 134.3, 133.9, 130.2, 130.1, 129.7, 129.5, 129.4, 129.4, 129.4, 129.1, 128.3, 128.2, 127.5, 127.5, 127.5, 126.6, 125.9, 125.5, 125.4, 124.4, 84.7, 81.2, 55.7, 54.3, 28.3, 27.8. HRMS (ESI): calcd *m/z* for $C_{22}H_{22}NO_6$: 396.1447 $[M-H]^-$; found: 396.1450.

2.2.2 Nucleophilic Ring-Opening of the Isoxazolidin-5-one.

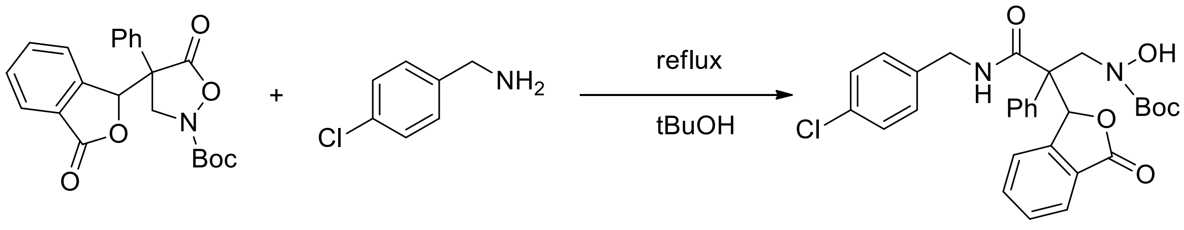

7a **9**

In a pressure Schlenk-tube, product **7a** (0.06 mmol) and p-chloro benzylamine (0.06 mmol) were dissolved in tBuOH and stirred at 90 °C overnight. Volatiles were removed in vacuo and the crude mixture was purified by column chromatography (silica gel, heptanes/EtOAc) to yield amide **9** (16 mg, 0.029 mmol).

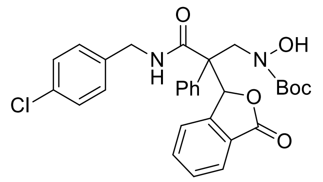

9

^{1}H NMR (300 MHz, δ, $CDCl_3$, 298 K): 7.81-7.77 (m, 1H), 7.62-7.55 (m, 2H), 7.45-7.40 (m, 1H), 7.32-7.29 (m, 1H), 7.24-7.16 (m, 5H), 7.07 (d, *J* = 8.2 Hz, 3H), 6.55 (s, 1H), 6.05 (t, *J*= 5.7 Hz, 1H), 4.62 (d, *J*= 14.8 Hz, 1H), 4.42 (d, *J*= 14.8 Hz, 2H), 4.14 (dd, J_1= 5.1 Hz, J_2 = 9.1 Hz, 1H), 1.37 (s, 9H). ^{13}C NMR (75 MHz, δ, $CDCl_3$, 298 K): 169.8, 156.7, 146.8, 135.9, 133.8, 133.4, 129.9, 129.5, 129.3, 128.9, 128.9, 128.8, 128.7, 128.1, 126.7, 125.3, 82.8, 59.6, 55.2, 43.4, 28.1. HRMS (ESI): calcd *m/z* for $C_{29}H_{29}ClN_2O_6$: 559.1612 $[M+Na]^+$; found: 559.1603.

3. Computational Details

To assess the relative configuration, DFT calculations were performed using Gaussian 16.[2] To this end, the structure of compound **7a** was optimized in both *R*,*R*- and *R*,*S*-configuration using (keeping the stereocenter of the isoxazolidine configured as *R*) using B3LYP/6-31G(d). Applying this method, possible conformations were investigated and the structures lowest in energy were reoptimized using B3LYP/6-311+G(2d,p). For all optimizations performed, the implicit solvent description IEFPCM for chloroform as polarized continuum was used as implemented in Gaussian 16.

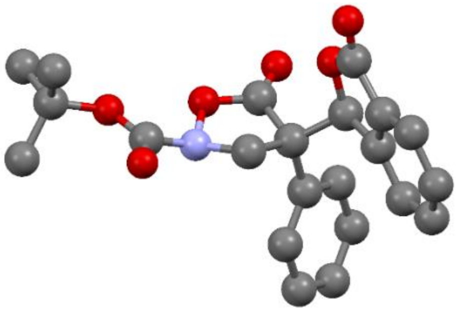

R,*S*-**7a**

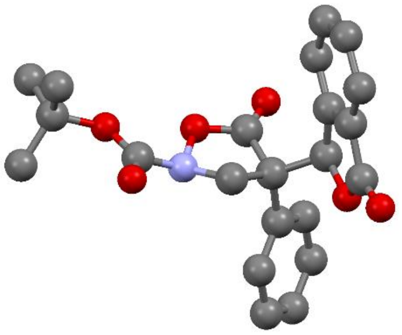

R,*R*-**7a**
ΔE = +0.6 kJ mol^{-1}

Figure S1. The two optimized structures for the two diastereomers of **7a**, hydrogen atoms omitted for clarity.

For each diastereomeric structure lowest in energy, isotropic chemical shifts for ^{13}C were computed by GIAO-NMR calculateions, applying different methods and scaling parameters as reported by Pierens[3] (Table S1). The methods were chosen according to their performance for ^{13}C-NMR predictions in chloroform and their accuracy for a compound of similar complexity and size (Mexicalin).

Table S1. Methods and scaling parameters used for the ^{13}C-NMR calculations to deliver the corresponding shifts relative to TMS as reference.[3] In all cases, the IEFPCM description for chloroform as solvent was added.

Abbreviation	Method/Basis set	Slope	Intercept
M1	B3LYP/6-311+G(2d,p)	-1.0427	181.7173
M2	mPW1PW91/6-311+G(2d,p)	-1.0420	186.3567
M3	PBE0/6-311+G(2d,p)	-1.0423	187.1937
M4	B3LYP/aug-cc-pvdz	-0.9974	190.9642

[2] Gaussian 16, Revision A.03, M. J. Frisch, G. W. Trucks, H. B. Schlegel, G. E. Scuseria, M. A. Robb, J. R. Cheeseman, G. Scalmani, V. Barone, G. A. Petersson, H. Nakatsuji, X. Li, M. Caricato, A. V. Marenich, J. Bloino, B. G. Janesko, R. Gomperts, B. Mennucci, H. P. Hratchian, J. V. Ortiz, A. F. Izmaylov, J. L. Sonnenberg, D. Williams-Young, F. Ding, F. Lipparini, F. Egidi, J. Goings, B. Peng, A. Petrone, T. Henderson, D. Ranasinghe, V. G. Zakrzewski, J. Gao, N. Rega, G. Zheng, W. Liang, M. Hada, M. Ehara, K. Toyota, R. Fukuda, J. Hasegawa, M. Ishida, T. Nakajima, Y. Honda, O. Kitao, H. Nakai, T. Vreven, K. Throssell, J. A. Montgomery, Jr., J. E. Peralta, F. Ogliaro, M. J. Bearpark, J. J. Heyd, E. N. Brothers, K. N. Kudin, V. N. Staroverov, T. A. Keith, R. Kobayashi, J. Normand, K. Raghavachari, A. P. Rendell, J. C. Burant, S. S. Iyengar, J. Tomasi, M. Cossi, J. M. Millam, M. Klene, C. Adamo, R. Cammi, J. W. Ochterski, R. L. Martin, K. Morokuma, O. Farkas, J. B. Foresman, and D. J. Fox, Gaussian, Inc., Wallingford CT, **2016**.
[3] G. K. Pierens, *J. Comp. Chem.* **2014**, *35*, 1388–1394.

Calculated shifts (relative to tetramethylsilane (TMS)) are given in Table S2 and Table S3, respectively. The atom numbering in the Tables correspond to the atom numbers in the coordinate lists. The assigned numbers do not change between the two diastereomers and are shown for *R*,*R*-**7a** in Figure S2.

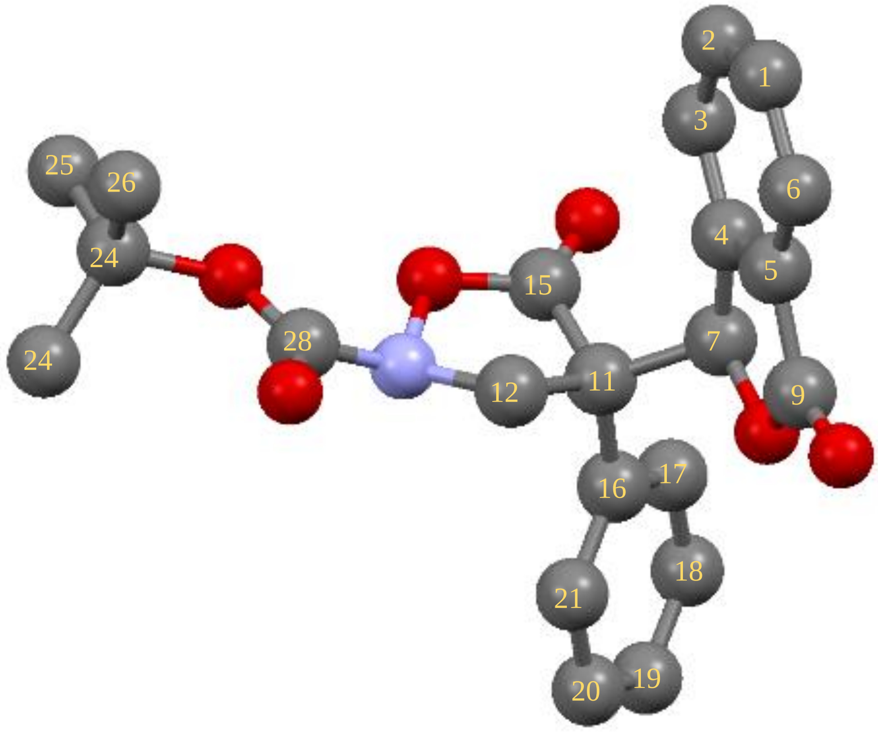

Figure S2. Carbon atom numbering demonstrated for the *R*,*R*-structure. The other diastereomer's atom assignment is identical.

For equivalent carbons 17&20 (*ortho*-C of the phenyl group), 18&21 (*meta*-C of the phenyl group) and 25-27 (methyl groups in *t*-Bu group), due to the non-dynamic nature of the calculations, different shifts with high anisotropy were obtained from the calculations. Unweighted averages were calculated for these atoms and are represented as such in the Tables S2-S6. Further analyses ($MAE_{\Delta\delta}$, $MAE_{\Delta\Delta\delta}$), were carried out accounting for all atoms (indicated by the index: *all*) and, as well as without taking those averaged signals into account (indicated by the index: *sel*).

Table S2. Experimentally obtained ^{13}C-NMR shifts compared to the computationally obtained shifts for the *unlike*-configuration (*R*,*S*). Experimental shifts were referenced to the solvent signal, calculated values are relative to TMS.

**Atom-Nr.**	**Experimental shifts: δ_{exp} / ppm**		**Computational shifts: δ_{RS} / ppm**			
	**major**	**minor**	**M1**	**M2**	**M3**	**M4**
C-1	130.1	130.3	132.0	132.1	132.1	127.4
C-2	134.3	134.4	136.5	136.7	136.7	131.2
C-3	124.4	123.5	124.5	124.7	124.7	120.5
C-4	144.9	144.7	150.4	149.4	149.4	145.5
C-5	126.6	126.8	128.7	128.3	128.2	126.0
C-6	125.9	126.2	127.6	128.1	128.1	122.6
C-7	81.2	81.3	81.0	79.7	79.7	83.7
C-9	169.2	169.0	175.5	174.6	174.5	170.1
C-11	55.7	57.5	56.0	55.0	54.9	60.9
C-12	54.3	56.7	44.0	43.2	43.2	47.7
C-15	173.1	172.0	178.0	176.8	176.7	172.3
C-16	129.7	129.8	135.0	133.7	133.7	133.0
C-17/21	127.5	127.8	129.2	129.2	129.2	125.1
C-18/20	129.4	129.7	131.3	131.4	131.5	126.3
C-19	130.2	133.3	131.5	131.7	131.7	126.3
C-24	84.7	84.5	87.2	85.6	85.5	89.6
C-25-27	27.8	27.8	19.7	19.6	19.7	25.7
C-28	155.8	156.0	161.0	160.2	160.1	156.6
C-17			130.6	130.6	130.6	126.5
C-18			131.6	131.7	131.8	126.7
C-20			130.9	131.1	131.1	125.9
C-21			127.7	127.8	127.7	123.7
C-25			23.4	23.1	23.2	29.7
C-26			18.1	18.1	18.2	24.2
C-27			17.5	17.6	17.7	23.4

Table S3. Experimentally obtained ^{13}C-NMR shifts compared to the computationally obtained shifts for the *like*-configuration (*R*,*R*). Experimental shifts were referenced to the solvent signal, calculated values are relative to TMS.

Atom-Nr.	Experimental shifts: δ_{exp} / ppm		Computational shifts: δ_{RS} / ppm			
	major	minor	M1	M2	M3	M4
C-1	130.1	130.3	132.2	132.3	132.4	127.3
C-2	134.3	134.4	137.4	137.5	137.6	131.7
C-3	124.4	123.5	124.9	125.1	125.0	121.3
C-4	144.9	144.7	150.5	149.3	149.3	145.4
C-5	126.6	126.8	129.1	128.6	128.6	125.9
C-6	125.9	126.2	128.0	128.4	128.4	123.4
C-7	81.2	81.3	85.5	84.0	84.1	87.4
C-9	169.2	169.0	175.0	174.2	174.1	169.5
C-11	55.7	57.5	57.6	56.5	56.4	62.1
C-12	54.3	56.7	45.8	45.0	45.0	49.4
C-15	173.1	172.0	178.6	177.5	177.4	172.8
C-16	129.7	129.8	137.1	135.8	135.8	134.7
C-17/21	127.5	127.8	128.3	128.4	128.3	124.2
C-18/20	129.4	129.7	130.9	131.0	131.1	125.9
C-19	130.2	133.3	130.9	131.1	131.1	126.2
C-24	84.7	84.5	87.4	85.7	85.6	89.8
C-25-27	27.8	27.8	19.6	19.5	19.6	25.7
C-28	155.8	156.0	160.6	159.8	159.7	156.4
C-17			129.4	129.4	129.4	125.0
C-18			131.1	131.2	131.3	126.0
C-20			130.6	130.7	130.8	125.8
C-21			127.2	127.3	127.2	123.4
C-25			23.3	23.0	23.1	29.8
C-26			18.1	18.1	18.2	24.1
C-27			17.4	17.5	17.5	23.3

$MAE_{\Delta\delta}$ analysis

A mean absolute error (MAE) analysis was performed. For each method, the absolute differences in chemical shift $\Delta\delta = | \delta_{exp} - \delta_{calc} |$ were calculated between the experimental shifts for major and minor diastereomer and the calculated shifts for the *R*,*S*-structure (Table S4) and the *R*,*R*-configuration (Table S5), respectively. The obtained $MAE_{\Delta\delta}$ values were found to be generally quite large, even when omitting the averaged shifts

mentioned above. Additionally, in the results for all methods, both for the *like*- and *unlike*-isomer, lower MAE values for the major product were obtained. Hence, this method was disregarded.

Table S4. Absolute deviations in chemical shift $\Delta\delta = |\ \delta_{exp} - \delta_{calc}\ |$ for the *unlike*-configuration (*R*,*S*). Mean absolute errors calculated for all carbons ($MAE_{\Delta\delta,all}$) and for a selection ($MAE_{\Delta\delta,sel}$) excluding highly anisotropic shifts due to the static nature of the calculations (*t*-Bu-group, carbons in *o*- and *m*-position of Ph).

**Atom-**	**M1:Δ*δ***		**M2: Δ*δ***		**M3: Δ*δ***		**M4: Δ*δ***	
**Nr.**	**maj-RS**	**min-RS**	**maj-RS**	**min-RS**	**maj-RS**	**min-RS**	**maj-RS**	**min-RS**
C-1	1.88	1.67	1.98	1.77	1.99	1.78	2.70	2.91
C-2	2.28	2.19	2.46	2.36	2.44	2.35	3.01	3.11
C-3	0.18	1.08	0.36	1.26	0.34	1.24	3.83	2.93
C-4	5.54	5.75	4.49	4.70	4.50	4.71	0.57	0.78
C-5	2.17	1.90	1.70	1.43	1.69	1.42	0.60	0.87
C-6	1.74	1.45	2.22	1.93	2.20	1.91	3.24	3.53
C-7	0.23	0.30	1.50	1.57	1.47	1.54	2.53	2.46
C-9	6.24	6.42	5.38	5.56	5.30	5.48	0.84	1.02
C-11	0.25	1.46	0.79	2.50	0.86	2.57	5.15	3.44
C-12	10.26	12.62	11.09	13.45	11.14	13.50	6.59	8.95
C-15	4.82	5.95	3.66	4.79	3.58	4.71	0.84	0.29
C-16	5.23	5.13	3.94	3.84	3.98	3.88	3.25	3.15
C-17/21	1.66	1.34	1.68	1.36	1.65	1.33	2.40	2.72
C-18/20	1.91	1.59	2.05	1.73	2.11	1.79	3.07	3.39
C-19	1.29	1.81	1.48	1.61	1.52	1.57	3.85	6.94
C-24	2.58	2.70	0.91	1.03	0.84	0.96	4.95	5.07
C-25-27	8.14	8.09	8.20	8.15	8.12	8.07	2.06	2.01
C-28	5.13	5.00	4.38	4.25	4.26	4.13	0.79	0.66
**$MAE_{\Delta\delta,all}$**	**3.42**	**3.69**	**3.24**	**3.52**	**3.22**	**3.50**	**2.79**	**3.01**
**$MAE_{\Delta\delta,sel}$**	**3.61**	**3.84**	**3.41**	**3.65**	**3.39**	**3.63**	**2.73**	**3.02**

Table S5. Absolute deviations in chemical shift $\Delta\delta = |\ \delta_{exp} - \delta_{calc}\ |$ for the *like*-configuration (*R*,*R*). Mean absolute errors calculated for all carbons ($MAE_{\Delta\delta,all}$) and for a selection ($MAE_{\Delta\delta,sel}$) excluding highly anisotropic shifts due to the static nature of the calculations (*t*-Bu-group, carbons in *o*- and *m*-position of Ph).

**Atom-Nr.**	**M1: Δδ** maj-RR	**M1: Δδ** min-RR	**M2: Δδ** maj-RR	**M2: Δδ** min-RR	**M3: Δδ** maj-RR	**M3: Δδ** min-RR	**M4: Δδ** maj-RR	**M4: Δδ** min-RR
C-1	2.13	1.92	2.25	2.04	2.30	2.09	2.79	3.00
C-2	3.16	3.06	3.28	3.18	3.30	3.20	2.51	2.60
C-3	0.50	1.40	0.70	1.60	0.69	1.59	3.02	2.12
C-4	5.55	5.76	4.39	4.60	4.41	4.62	0.46	0.67
C-5	2.54	2.27	2.09	1.82	2.07	1.80	0.67	0.94
C-6	2.09	1.80	2.56	2.27	2.51	2.22	2.45	2.74
C-7	4.29	4.22	2.85	2.78	2.88	2.81	6.24	6.17
C-9	5.80	5.98	4.96	5.14	4.89	5.07	0.29	0.47
C-11	1.90	0.19	0.74	0.97	0.68	1.03	6.35	4.64
C-12	8.51	10.87	9.28	11.64	9.34	11.70	4.92	7.28
C-15	5.43	6.56	4.31	5.44	4.22	5.35	0.31	0.82
C-16	7.35	7.25	6.05	5.95	6.09	5.99	4.93	4.83
C-17/21	0.77	0.45	0.83	0.51	0.79	0.47	3.35	3.67
C-18/20	1.48	1.16	1.62	1.30	1.69	1.37	3.45	3.77
C-19	0.69	2.41	0.88	2.21	0.93	2.17	3.98	7.08
C-24	2.70	2.82	1.03	1.15	0.97	1.09	5.15	5.27
C-25-27	8.23	8.18	8.29	8.24	8.21	8.16	2.09	2.04
C-28	4.71	4.58	3.95	3.82	3.83	3.70	0.54	0.41
**$MAE_{\Delta\delta,all}$**	**3.77**	**3.94**	**3.34**	**3.59**	**3.32**	**3.58**	**2.97**	**3.25**
**$MAE_{\Delta\delta,sel}$**	**3.96**	**4.09**	**3.49**	**3.71**	**3.48**	**3.70**	**2.97**	**3.32**

$MAE_{\Delta\Delta\delta}$ analysis

Since simple MAE (mean absolute error) analyses remained inconclusive and generally showed quite high deviations, an $MAE_{\Delta\Delta\delta}$ approach as reported by the group of Bifulco was undertaken.[4]

The $MAE_{\Delta\Delta\delta}$ is defined as:

$$MAE_{\Delta\Delta\delta} = \frac{\sum(\Delta\Delta\delta)}{n_{\Delta\Delta\delta}} = \frac{\sum(|\Delta\delta_{calc} - \Delta\delta_{exp}|)}{n_{\Delta\Delta\delta}}$$

Herein, the term $\Delta\Delta\delta = |\ \Delta\delta_{calc} - \Delta\delta_{exp}\ |$ is calculated as the absolute difference in chemical shift $\Delta\delta_{calc}$ between the calculated diastereomers (*R*,*S* and *R*,*R*) and the difference in chemical shift $\Delta\delta_{exp}$ obtained experimentally. Two separate values for $\Delta\Delta\delta$ are then calculated for each atom with both $\Delta\delta_{exp} = \delta_{major} - \delta_{minor}$ as well as

[4] G. Lauro, P. Das, R. Riccio, D. S. Reddy, G. Bifulco, *J. Org. Chem.* **2020**, *85*, 3297–3306.

$\Delta\delta_{exp} = \delta_{minor} - \delta_{major}$. In this context, the calculation with the difference $\Delta\delta_{exp} = \delta_{major} - \delta_{minor}$ corresponds to the correlation between the *R*,*S*-isomer and the major product (*vice versa*), which is ultimately accounted for all atoms as the $MAE_{\Delta\Delta\delta}$.

As can be seen from the results in Table S6, with all methods, a clearly better correlation between the *unlike*-isomer and the experimentally observed major diastereomer was obtained as compared to the minor one. The selected atom set (discarded *o*- and *m*-carbons of the Ph group as well as Me-signals from the *t*-Bu group), yielded even higher differences in the $MAE_{\Delta\Delta\delta}$. This leads us to assume an *unlike*-configuration for the major diastereoisomer.

Table S6. Results of the $MAE_{\Delta\Delta\delta}$ assessment. Mean absolute errors calculated for all carbons ($MAE_{\Delta\Delta\delta,all}$) and for a selection ($MAE_{\Delta\Delta\delta,sel}$) excluding highly anisotropic shifts due to the static nature of the calculations (*t*-Bu-group, aromatic carbons in ortho- and meta-position).

	**M1: ΔΔ*δ***		**M2: ΔΔ*δ***		**M3: ΔΔ*δ***		**M4: ΔΔ*δ***	
**Atom-Nr.**	**RS-RR, maj-min**	**RS-RR, min-maj**	**RS-RR, maj-min**	**RS-RR, min-maj**	**RS-RR, maj-min**	**RS-RR, min-maj**	**RS-RR, maj-min**	**RS-RR, min-maj**
C-1	0.04	0.46	0.06	0.48	0.10	0.52	0.30	0.12
C-2	0.88	0.88	0.82	0.82	0.86	0.86	0.50	0.50
C-3	1.21	0.59	1.23	0.57	1.25	0.55	1.71	0.09
C-4	0.23	0.20	0.11	0.31	0.12	0.31	0.10	0.32
C-5	0.09	0.63	0.12	0.66	0.11	0.65	0.34	0.20
C-6	0.06	0.64	0.05	0.63	0.03	0.61	0.50	1.08
C-7	4.44	4.58	4.27	4.41	4.28	4.42	3.64	3.78
C-9	0.26	0.62	0.24	0.60	0.24	0.60	0.37	0.73
C-11	0.06	3.36	0.18	3.24	0.17	3.25	0.52	2.90
C-12	0.61	4.11	0.54	4.18	0.56	4.16	0.69	4.03
C-15	1.73	0.53	1.79	0.47	1.77	0.49	1.66	0.60
C-16	2.03	2.23	2.01	2.21	2.00	2.20	1.58	1.78
C-17/21	1.21	0.57	1.17	0.53	1.17	0.53	1.27	0.63
C-18/20	0.75	0.11	0.76	0.12	0.74	0.10	0.70	0.06
C-19	3.69	2.50	3.70	2.50	3.69	2.50	3.23	2.96
C-24	0.24	0.00	0.25	0.01	0.25	0.01	0.31	0.07
C-25-27	0.04	0.14	0.04	0.14	0.04	0.14	0.02	0.08
C-28	0.55	0.29	0.56	0.30	0.56	0.30	0.38	0.12
**$MAE_{\Delta\Delta\delta,all}$**	**1.06**	**1.25**	**0.99**	**1.23**	**1.00**	**1.23**	**0.99**	**1.11**
**$MAE_{\Delta\Delta\delta,sel}$**	**1.17**	**1.46**	**1.16**	**1.44**	**1.16**	**1.44**	**1.16**	**1.34**

Coordinates of the optimized geometries 7a

***R*,*S*-7a**

E (IEFPCM($CHCl_3$)-B3LYP/6-311+G(2d,p)) = -1357.492694

C	4.45403	-2.33810	-1.73917	C	-5.11023	-1.94299	-0.47075
C	4.47118	-0.93932	-1.74754	C	-5.47581	0.27886	-1.65365
C	3.66595	-0.19568	-0.88728	C	-2.65627	-0.19718	-0.69913
C	2.83228	-0.88721	-0.01744	O	-2.59204	-0.76895	-1.76761
C	2.82752	-2.27563	-0.00726	H	5.09418	-2.88444	-2.42033
C	3.62881	-3.02617	-0.85979	H	5.12802	-0.42185	-2.43613
C	1.87296	-0.40561	1.04355	H	3.70004	0.88503	-0.90368
O	1.33921	-1.62488	1.61513	H	3.60687	-4.10814	-0.82996
C	1.88727	-2.73741	1.02622	H	2.39245	0.11167	1.85064
O	1.58494	-3.84973	1.36962	H	0.06299	-0.03171	-1.49702
C	0.70615	0.49091	0.56068	H	-0.22752	-1.28181	-0.28120
C	-0.19921	-0.20475	-0.45955	H	2.05602	2.29701	2.09150
N	-1.50635	0.40011	-0.17360	H	2.95832	4.48718	1.47767
O	-1.55788	0.64108	1.24966	H	2.63103	5.37975	-0.81680
C	-0.28511	0.70933	1.71695	H	1.37809	4.03181	-2.48132
C	1.22926	1.87878	0.14291	H	0.49051	1.84377	-1.88782
C	1.92094	2.65631	1.07950	H	-5.87671	1.13981	0.91796
C	2.42516	3.90464	0.73626	H	-6.98306	-0.21283	0.62846
C	2.23999	4.40590	-0.54843	H	-5.61941	-0.40829	1.74180
C	1.54023	3.64995	-1.48071	H	-4.50072	-2.29589	-1.30013
C	1.03666	2.39804	-1.13791	H	-6.14021	-2.26624	-0.63502
O	-0.05976	0.93164	2.87189	H	-4.75479	-2.40190	0.45378
O	-3.71732	0.02505	0.05673	H	-6.52085	0.05250	-1.87497
C	-5.08133	-0.42174	-0.35697	H	-5.37881	1.36116	-1.54818
C	-5.94103	0.05616	0.80847	H	-4.86571	-0.05352	-2.49120

***R*,*R*-7a**

E (IEFPCM($CHCl_3$)-B3LYP/6-311+G(2d,p)) = -1357.491234

C	2.20865	-4.45548	0.36091	C	-4.87790	-1.85054	-0.61014
C	1.60480	-3.83890	1.46150	C	-5.43988	0.56402	-1.17884
C	1.54635	-2.45138	1.57848	C	-2.51579	0.03326	-0.69664
C	2.10103	-1.68952	0.55768	O	-2.56830	-0.27903	-1.86757
C	2.71038	-2.30805	-0.52557	H	2.23853	-5.53625	0.30350
C	2.77671	-3.69068	-0.64953	H	1.17734	-4.45325	2.24458
C	2.23940	-0.19468	0.38632	H	1.08844	-1.99378	2.44497
O	2.95298	-0.05023	-0.86600	H	3.26031	-4.14710	-1.50390
C	3.24636	-1.26994	-1.42061	H	2.86996	0.22715	1.17040
O	3.84526	-1.36525	-2.45969	H	0.06865	0.53833	-1.74634
C	0.94912	0.66024	0.28779	H	-0.00522	-0.95920	-0.81233
C	-0.04761	0.13143	-0.74938	H	2.15068	2.28063	2.11746
N	-1.32963	0.56964	-0.18469	H	2.82448	4.62651	1.91569
O	-1.20492	0.47500	1.25180	H	2.37194	5.88396	-0.17811
C	0.11336	0.52641	1.56619	H	1.23731	4.73982	-2.06488
C	1.31803	2.15211	0.13293	H	0.57427	2.40651	-1.88066
C	1.95494	2.80759	1.19212	H	-5.54159	0.77739	1.55188
C	2.33292	4.14005	1.08192	H	-6.64206	-0.52675	1.07745
C	2.07852	4.84496	-0.09009	H	-5.14704	-0.90447	1.94862
C	1.44255	4.20418	-1.14599	H	-4.36588	-1.96668	-1.56312
C	1.06390	2.86911	-1.03565	H	-5.91191	-2.18037	-0.72981
O	0.47813	0.47019	2.70808	H	-4.40162	-2.49403	0.13216
O	-3.47822	0.01073	0.20926	H	-6.49844	0.34134	-1.32763
C	-4.87224	-0.39881	-0.14050	H	-5.35663	1.59505	-0.82984
C	-5.59258	-0.25257	1.19563	H	-4.92962	0.46955	-2.13530

4. Copies of Product NMR Spectra and HPLC Chromatograms.

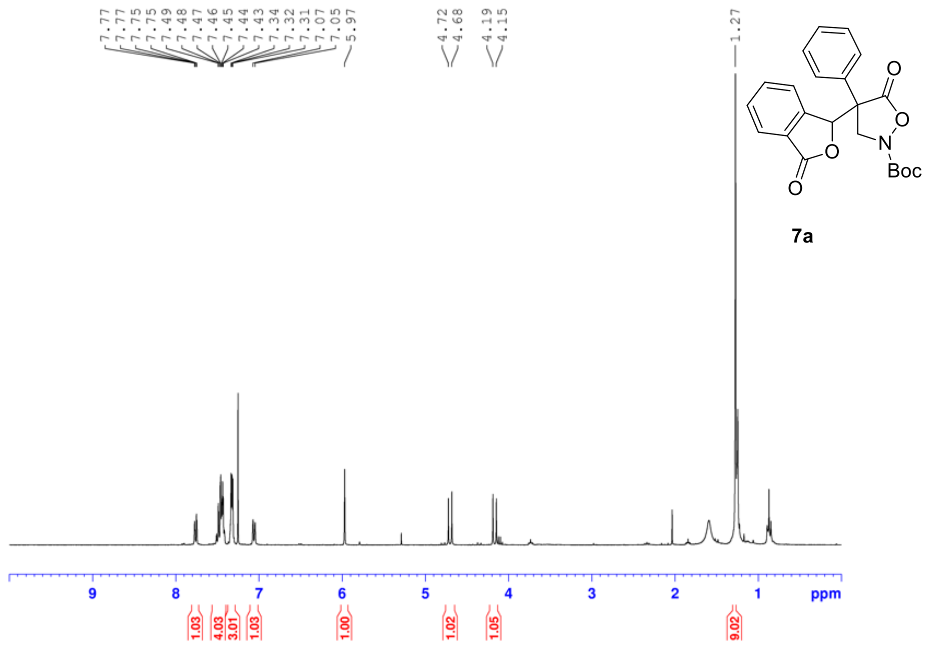

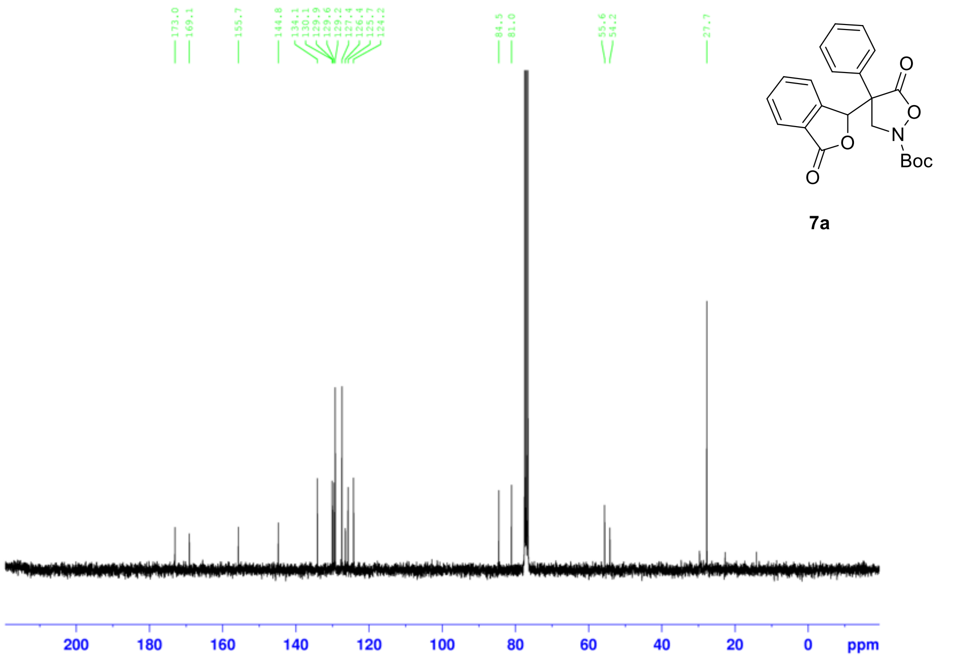

Chromatogram

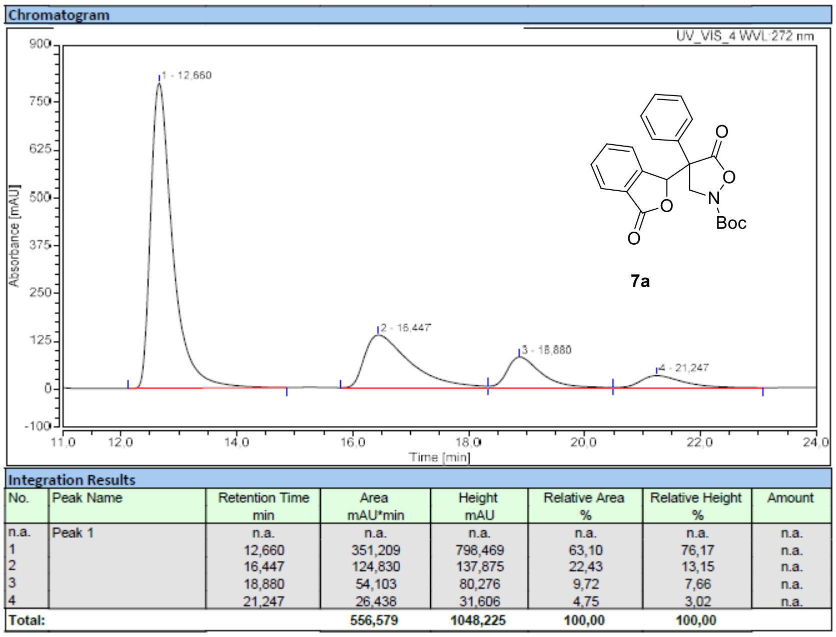

Integration Results

No.	Peak Name	Retention Time min	Area mAU*min	Height mAU	Relative Area %	Relative Height %	Amount
n.a.	Peak 1	n.a.	n.a.	n.a.	n.a.	n.a.	n.a.
1		12,660	351,209	798,469	63,10	76,17	n.a.
2		16,447	124,830	137,875	22,43	13,15	n.a.
3		18,880	54,103	80,276	9,72	7,66	n.a.
4		21,247	26,438	31,606	4,75	3,02	n.a.
Total:			556,579	1048,225	100,00	100,00	

Chromatogram

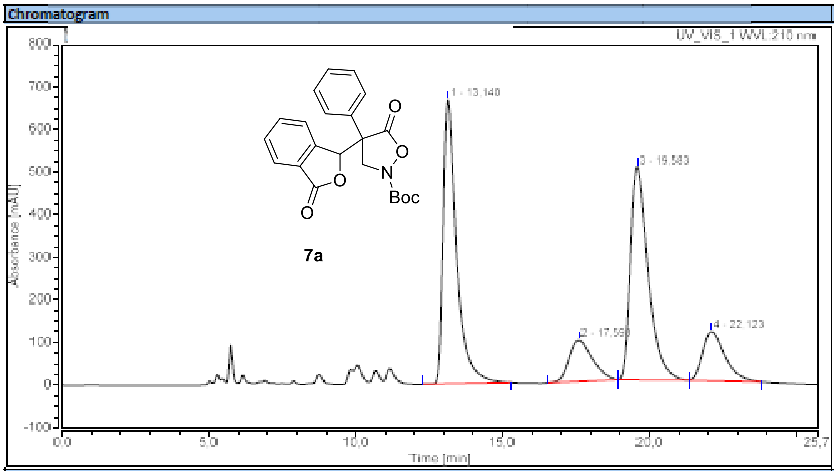

Integration Results

No.	Peak Name	Retention Time min	Area mAU*min	Height mAU	Relative Area %	Relative Height %	Amount
n.a.	Peak 1	n.a.	n.a.	n.a.	n.a.	n.a.	n.a.
1		13,140	354,992	668,051	39,65	48,49	n.a.
2		17,590	89,074	96,285	9,95	6,99	n.a.
3		19,583	349,916	499,069	39,08	36,23	n.a.
4		22,123	101,317	114,189	11,32	8,29	n.a.
Total:			895,299	1377,593	100,00	100,00	

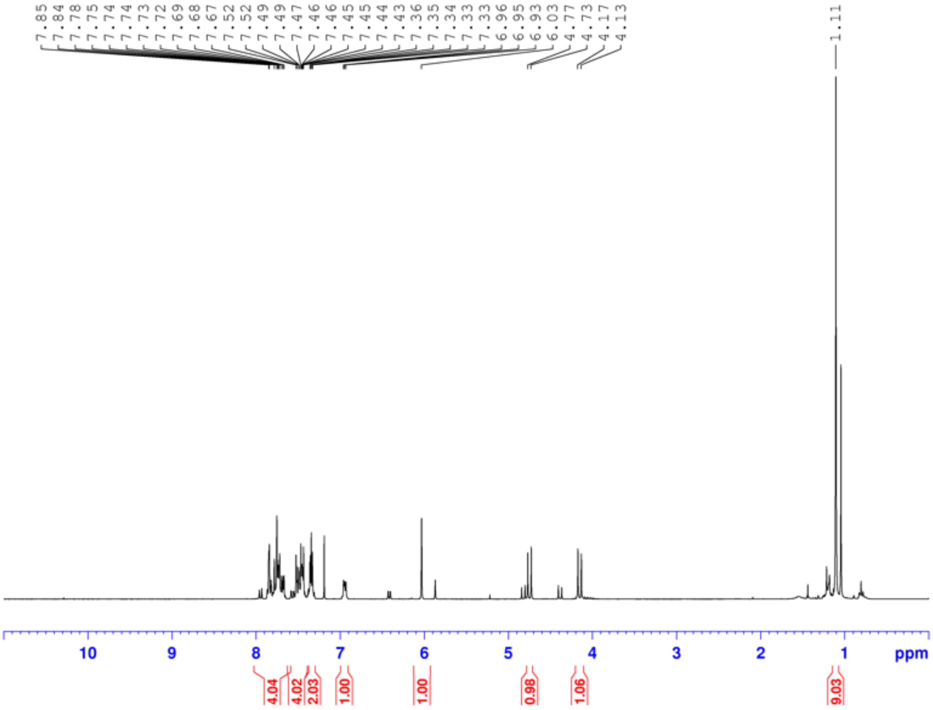
7.85
7.84
7.78
7.75
7.74
7.74
7.73
7.72
7.69
7.68
7.67
7.52
7.52
7.49
7.49
7.47
7.46
7.46
7.45
7.45
7.44
7.43
7.36
7.35
7.34
7.33
7.33
6.96
6.95
6.93
6.03
4.77
4.73
4.17
4.13
1.11
10
9
8
7
6
5
4
3
2
1
ppm
4.04
4.02
2.03
1.00
1.00
0.98
1.06
9.03

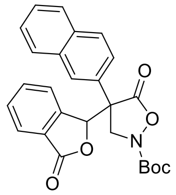
O
O
N
Boc
O
O

7b

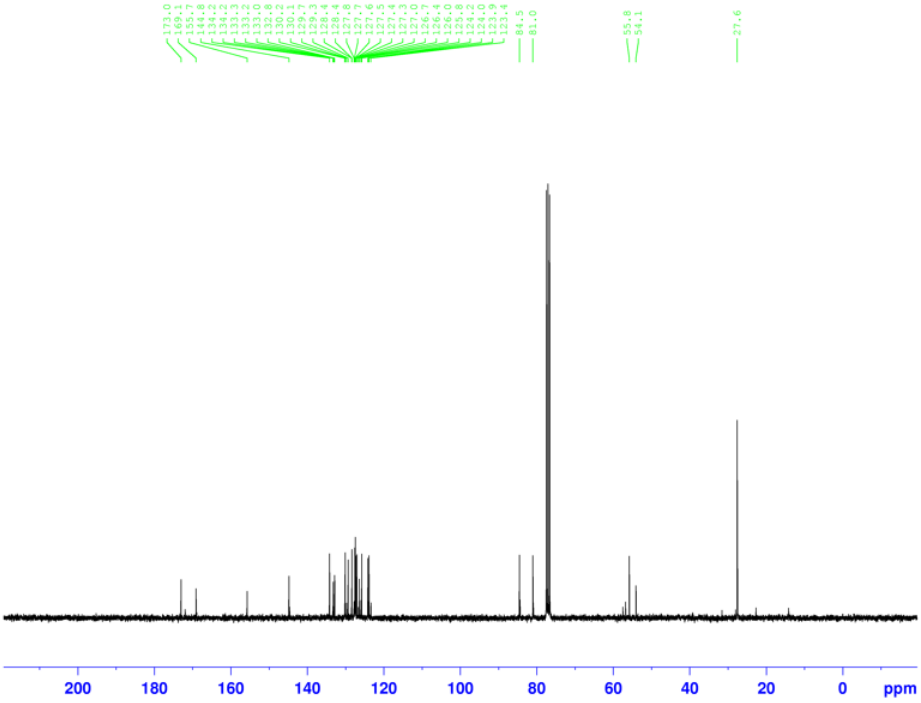
173.0
169.1
155.7
144.8
134.2
133.3
133.2
133.0
132.8
130.2
130.1
129.7
129.3
128.4
128.4
127.8
127.7
127.6
127.5
127.4
127.3
127.0
126.7
126.4
126.0
125.8
124.2
124.0
123.9
123.4
84.5
81.0
55.8
54.1
27.6
200
180
160
140
120
100
80
60
40
20
0
ppm

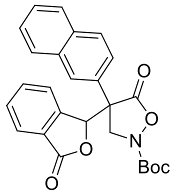
O
O
N
Boc
O
O

7b

Chromatogram

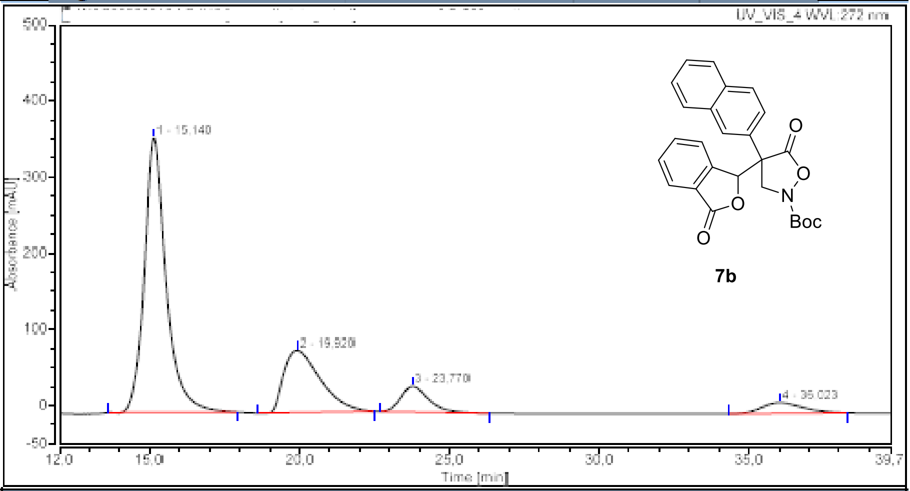

Integration Results

No.	Peak Name	Retention Time min	Area mAU*min	Height mAU	Relative Area %	Relative Height %	Amount
n.a.	Peak 1	n.a.	n.a.	n.a.	n.a.	n.a.	n.a.
1		15,140	304,063	361,082	63,55	73,66	n.a.
2		19,920	115,500	81,310	24,14	16,59	n.a.
3		23,770	36,759	33,848	7,68	6,90	n.a.
4		36,023	22,135	13,981	4,63	2,85	n.a.
**Total:**			**478,456**	**490,221**	**100,00**	**100,00**	

Chromatogram

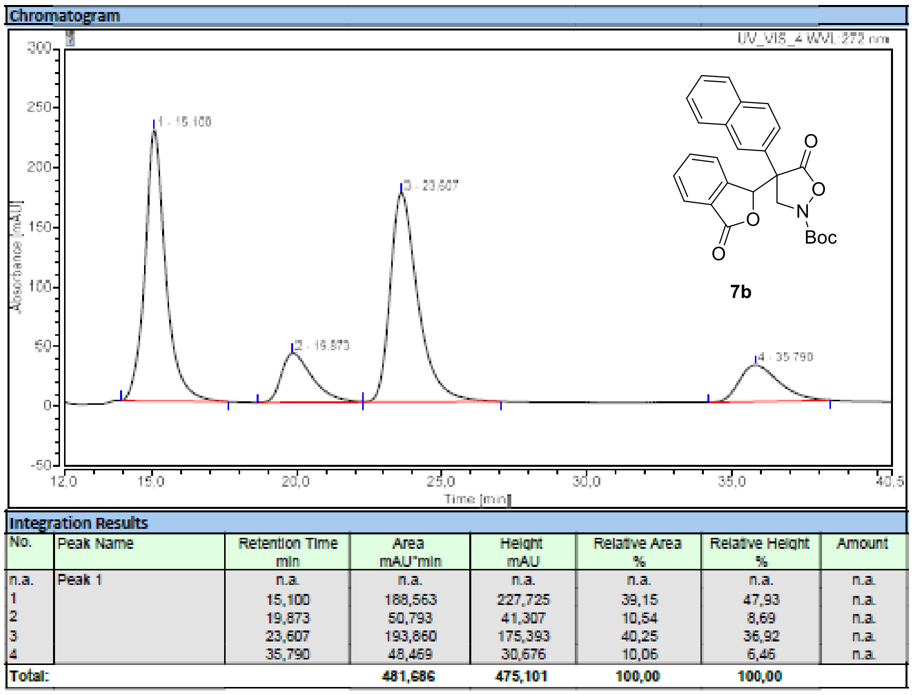

Integration Results

No.	Peak Name	Retention Time min	Area mAU*min	Height mAU	Relative Area %	Relative Height %	Amount
n.a.	Peak 1	n.a.	n.a.	n.a.	n.a.	n.a.	n.a.
1		15,100	188,563	227,725	39,15	47,93	n.a.
2		19,873	50,793	41,307	10,54	8,69	n.a.
3		23,607	193,860	175,393	40,25	36,92	n.a.
4		35,790	48,469	30,676	10,06	6,46	n.a.
**Total:**			**481,686**	**475,101**	**100,00**	**100,00**	

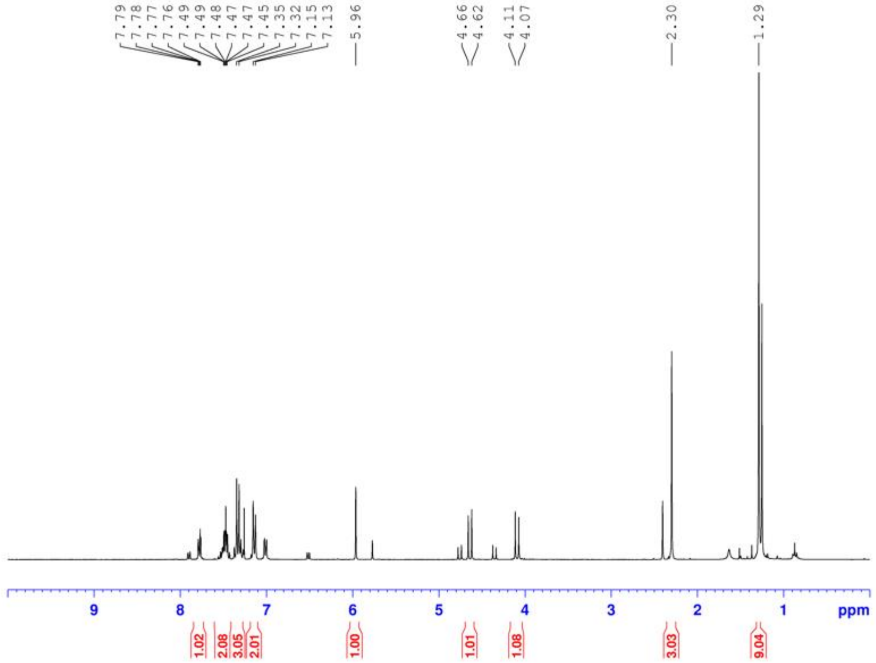

7.79
7.78
7.77
7.76
7.49
7.49
7.48
7.47
7.47
7.45
7.35
7.32
7.15
7.13
5.96
4.66
4.62
4.11
4.07
2.30
1.29
9
8
7
6
5
4
3
2
1
ppm
1.02
2.08
3.05
2.01
1.00
1.01
1.08
3.03
9.04

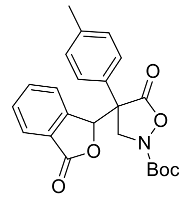

O
O
O
N
O
Boc

7d

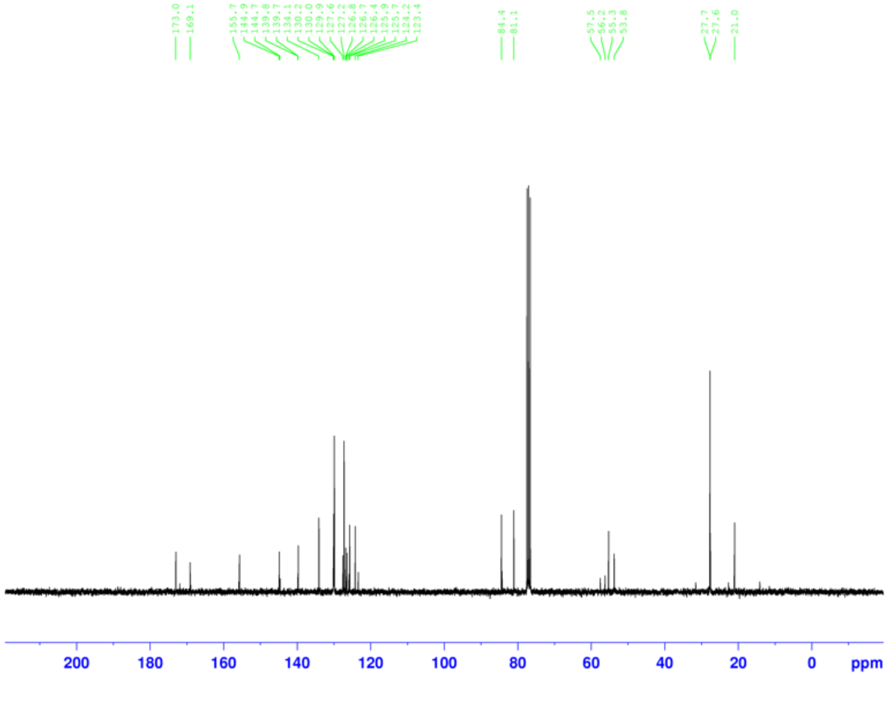

173.0
169.1
155.7
144.9
144.7
139.8
139.7
134.1
130.2
130.0
129.9
127.6
127.2
126.8
126.7
126.4
125.9
125.7
124.2
123.4
84.4
81.1
57.5
56.2
55.3
53.8
27.7
21.6
21.0
200
180
160
140
120
100
80
60
40
20
0
ppm

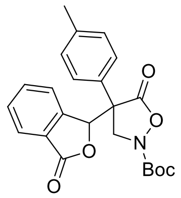

O
O
O
N
O
Boc

7d

Chromatogram

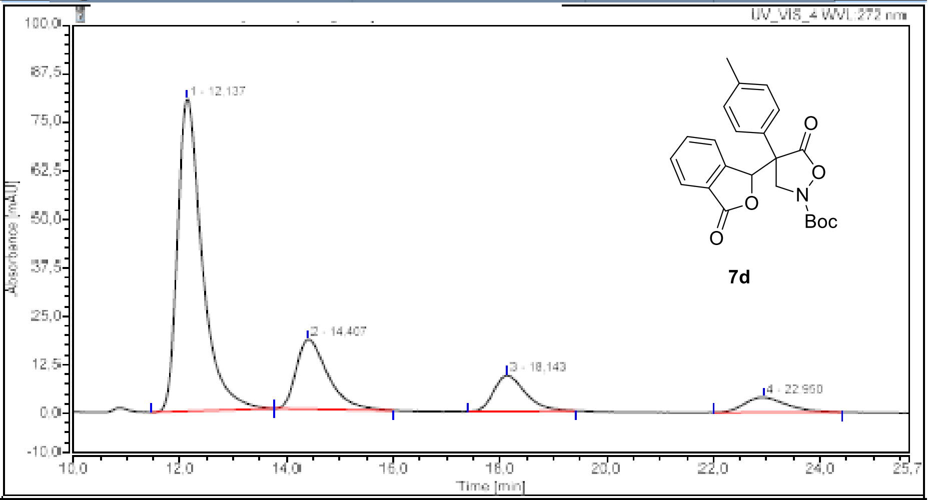

Integration Results

No.	Peak Name	Retention Time min	Area mAU*min	Height mAU	Relative Area %	Relative Height %	Amount
n.a.	Peak 1	n.a.	n.a.	n.a.	n.a.	n.a.	n.a.
1		12,137	43,096	80,468	66,00	72,19	n.a.
2		14,407	12,333	17,932	18,89	16,09	n.a.
3		18,143	6,339	9,203	9,71	8,26	n.a.
4		22,950	3,524	3,863	5,40	3,47	n.a.
Total:			65,292	111,466	100,00	100,00	

Chromatogram

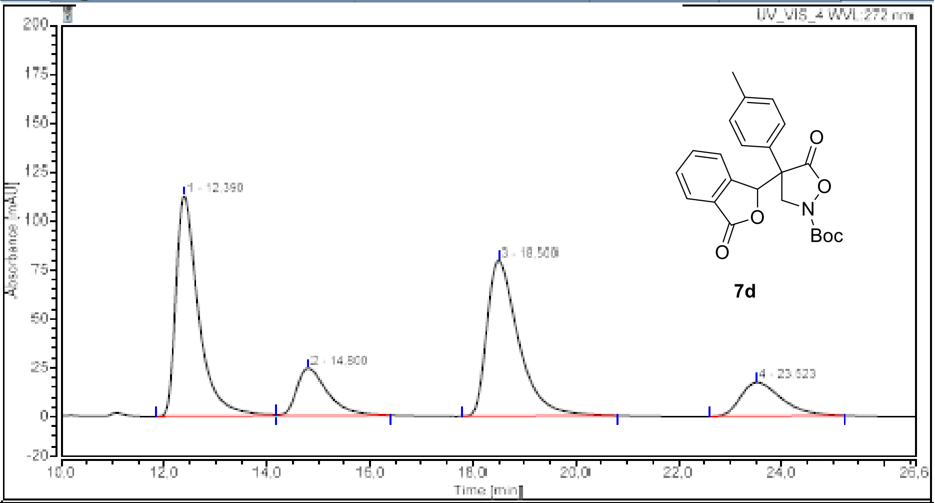

Integration Results

No.	Peak Name	Retention Time min	Area mAU*min	Height mAU	Relative Area %	Relative Height %	Amount
n.a.	Peak 1	n.a.	n.a.	n.a.	n.a.	n.a.	n.a.
1		12,390	57,495	112,528	39,01	48,19	n.a.
2		14,800	16,937	24,276	11,49	10,40	n.a.
3		18,500	56,886	79,402	38,60	34,01	n.a.
4		23,523	16,064	17,281	10,90	7,40	n.a.
Total:			147,382	233,488	100,00	100,00	

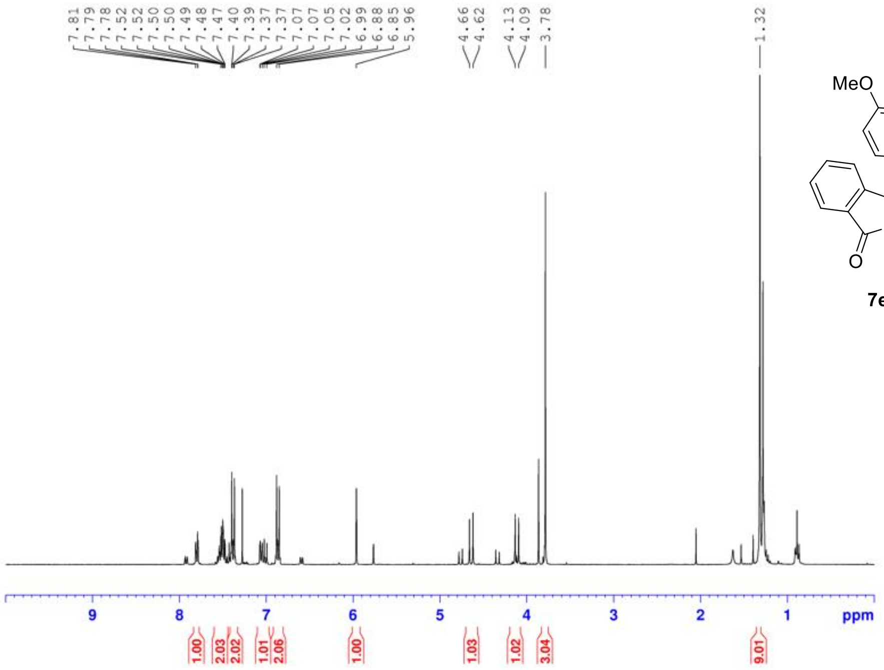
7.81
7.79
7.78
7.52
7.52
7.50
7.49
7.48
7.47
7.40
7.39
7.37
7.37
7.07
7.07
7.05
7.02
6.99
6.88
6.85
5.96
4.66
4.62
4.13
4.09
3.78
1.32
9
8
7
6
5
4
3
2
1
ppm
1.00
2.03
2.02
1.01
2.06
1.00
1.03
1.02
3.04
9.01

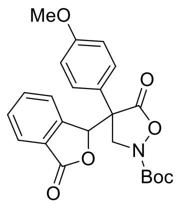
MeO
O
O
N
Boc
O
O

7e

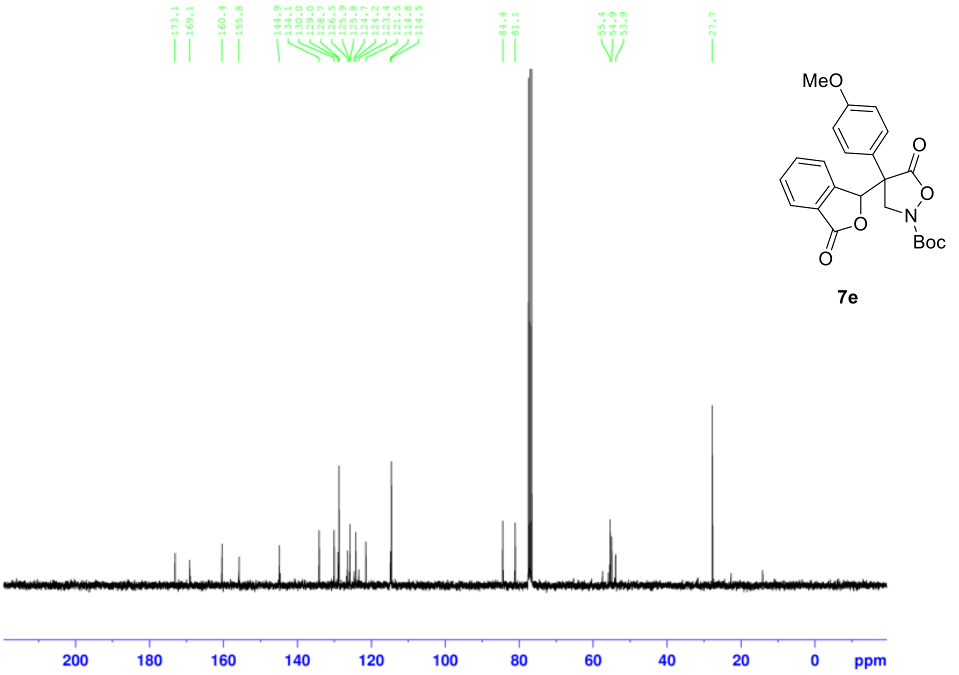
173.1
169.1
160.4
155.8
144.9
134.1
130.0
129.0
128.7
126.5
125.9
125.8
124.7
124.2
123.4
121.5
114.8
114.5
84.4
81.1
55.4
54.9
53.9
27.7
200
180
160
140
120
100
80
60
40
20
0
ppm
MeO
O
O
N
Boc
O
O

7e

Chromatogram

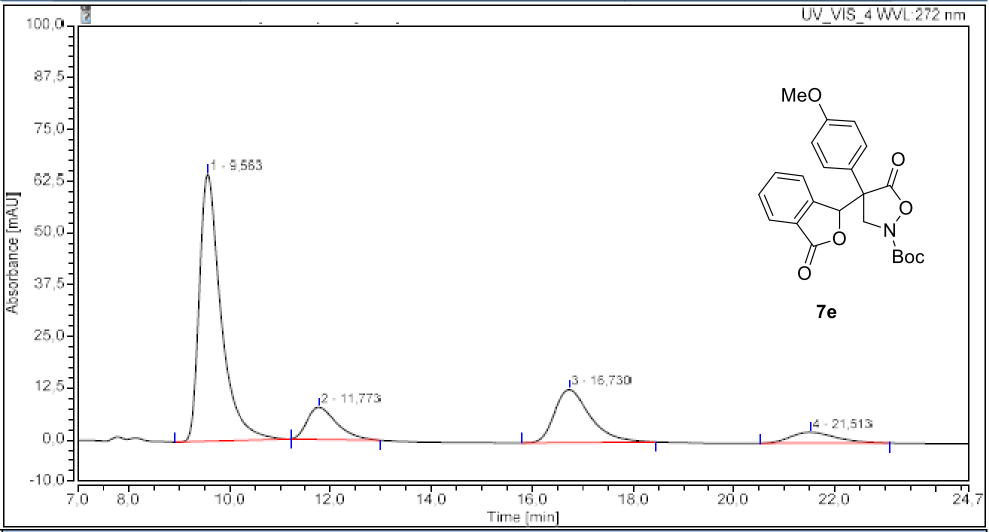

Integration Results

No.	Peak Name	Retention Time min	Area mAU*min	Height mAU	Relative Area %	Relative Height %	Amount
n.a.	Peak 1	n.a.	n.a.	n.a.	n.a.	n.a.	n.a.
1		9,563	31,811	64,396	63,35	73,67	n.a.
2		11,773	5,261	7,711	10,48	8,82	n.a.
3		16,730	10,433	12,707	20,78	14,54	n.a.
4		21,513	2,710	2,597	5,40	2,97	n.a.
**Total:**			**50,216**	**87,410**	**100,00**	**100,00**	

Chromatogram

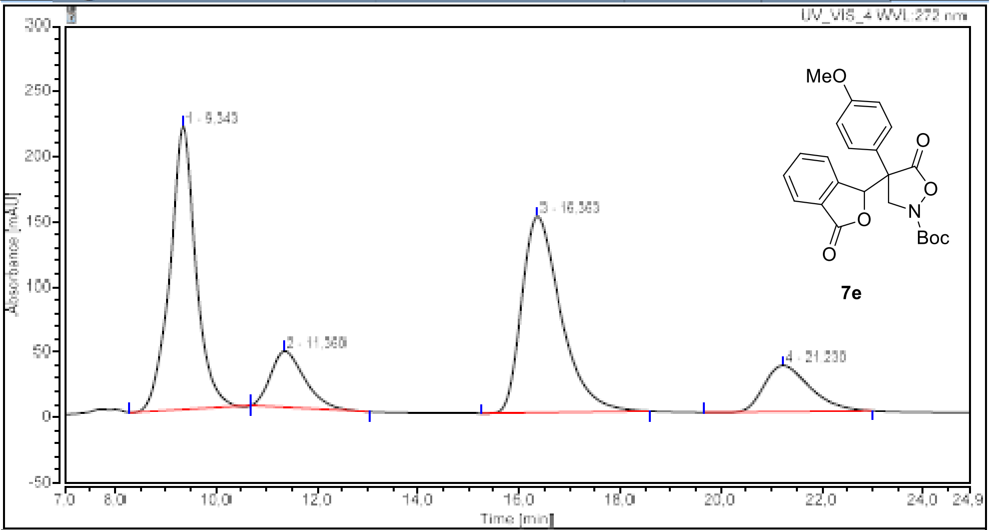

Integration Results

No.	Peak Name	Retention Time min	Area mAU*min	Height mAU	Relative Area %	Relative Height %	Amount
n.a.	Peak 1	n.a.	n.a.	n.a.	n.a.	n.a.	n.a.
1		9,343	130,458	217,121	38,72	48,70	n.a.
2		11,360	33,326	42,760	9,89	9,59	n.a.
3		16,363	135,861	150,582	40,33	33,78	n.a.
4		21,230	37,267	35,343	11,06	7,93	n.a.
**Total:**			**336,911**	**445,807**	**100,00**	**100,00**	

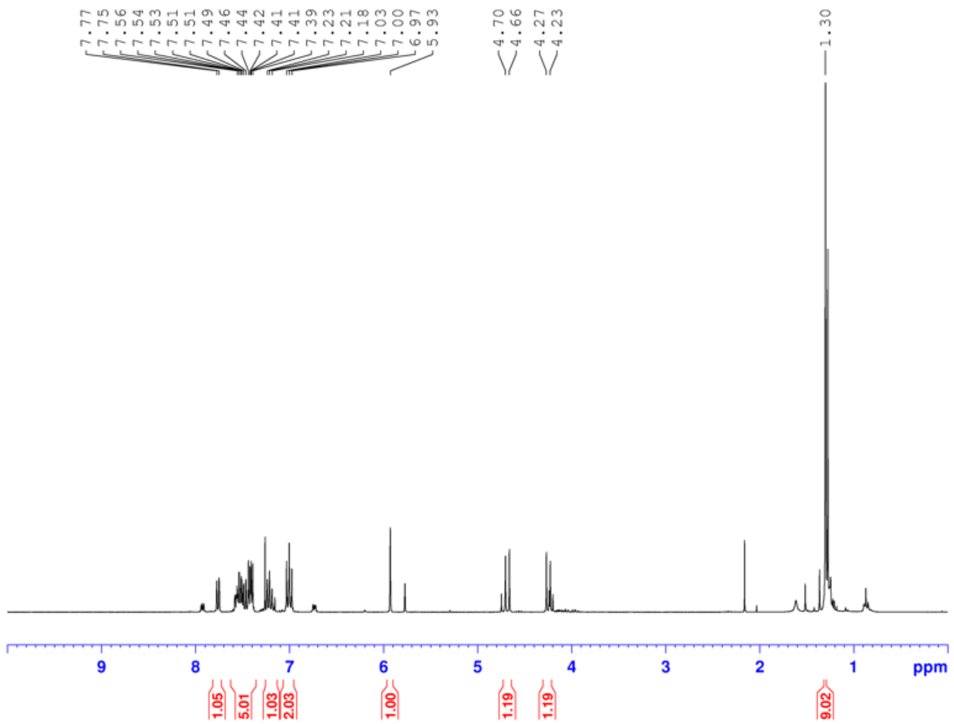
7.77
7.75
7.56
7.54
7.53
7.51
7.51
7.49
7.46
7.44
7.42
7.41
7.41
7.39
7.23
7.21
7.18
7.03
7.00
6.97
5.93
4.70
4.66
4.27
4.23
1.30
9
8
7
6
5
4
3
2
1
ppm
1.05
5.01
1.03
2.03
1.00
1.19
1.19
9.02

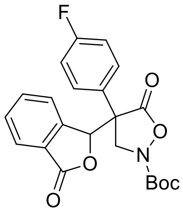
F
O
O
N
Boc
O
O

7f

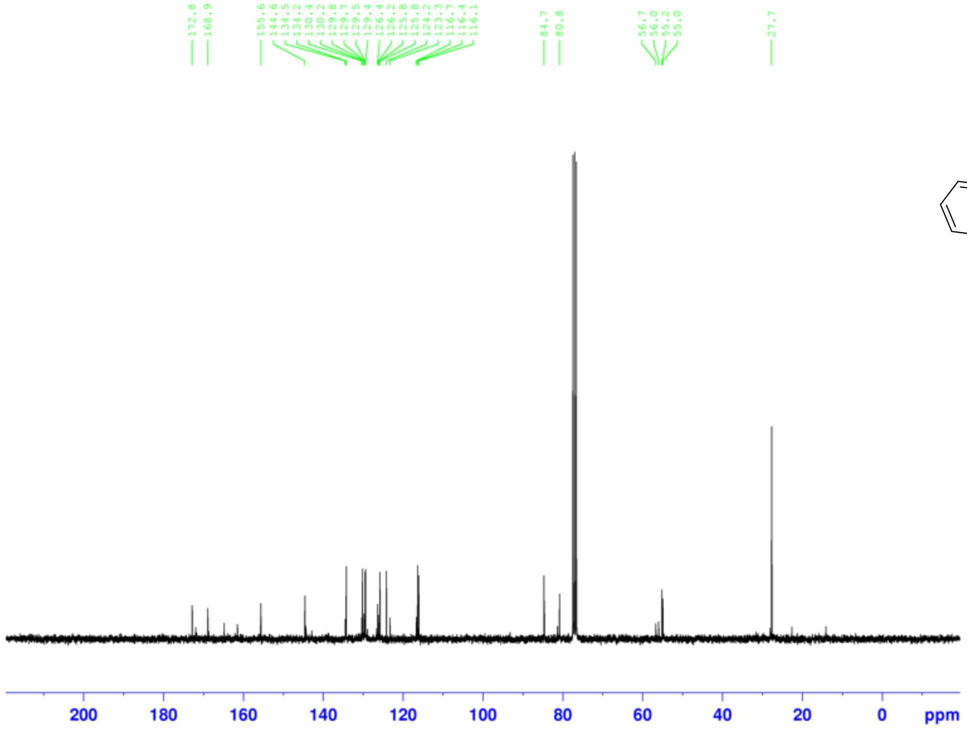
172.8
168.9
155.6
144.6
134.5
134.2
130.4
130.2
129.8
129.7
129.5
129.4
126.4
126.2
125.8
124.8
124.2
123.3
116.7
116.4
116.1
84.7
80.8
56.7
56.0
55.2
55.0
27.7
200
180
160
140
120
100
80
60
40
20
0
ppm

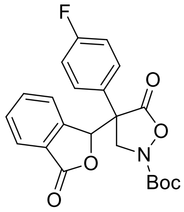
F
O
O
N
Boc
O
O

7f

Chromatogram

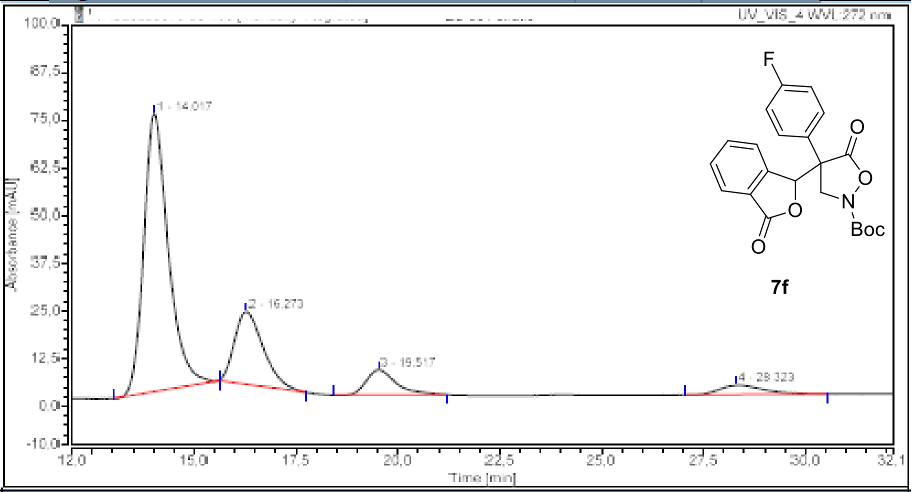

Integration Results

No.	Peak Name	Retention Time min	Area mAU*min	Height mAU	Relative Area %	Relative Height %	Amount
n.a.	Peak 1	n.a.	n.a.	n.a.	n.a.	n.a.	n.a.
1		14,017	50,553	72,827	67,69	72,24	n.a.
2		16,273	15,522	19,005	20,78	18,85	n.a.
3		19,517	5,581	6,527	7,47	6,47	n.a.
4		28,323	3,030	2,453	4,06	2,43	n.a.
**Total:**			**74,686**	**100,812**	**100,00**	**100,00**	

Chromatogram

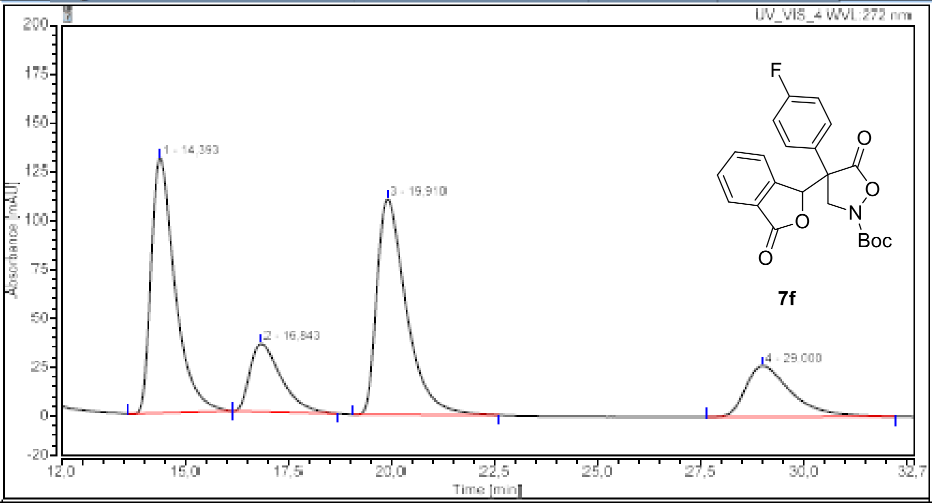

Integration Results

No.	Peak Name	Retention Time min	Area mAU*min	Height mAU	Relative Area %	Relative Height %	Amount
n.a.	Peak 1	n.a.	n.a.	n.a.	n.a.	n.a.	n.a.
1		14,393	87,719	130,249	36,13	43,25	n.a.
2		16,843	30,463	34,765	12,55	11,54	n.a.
3		19,910	91,316	110,154	37,61	36,57	n.a.
4		29,000	33,269	26,012	13,70	8,64	n.a.
**Total:**			**242,767**	**301,180**	**100,00**	**100,00**	

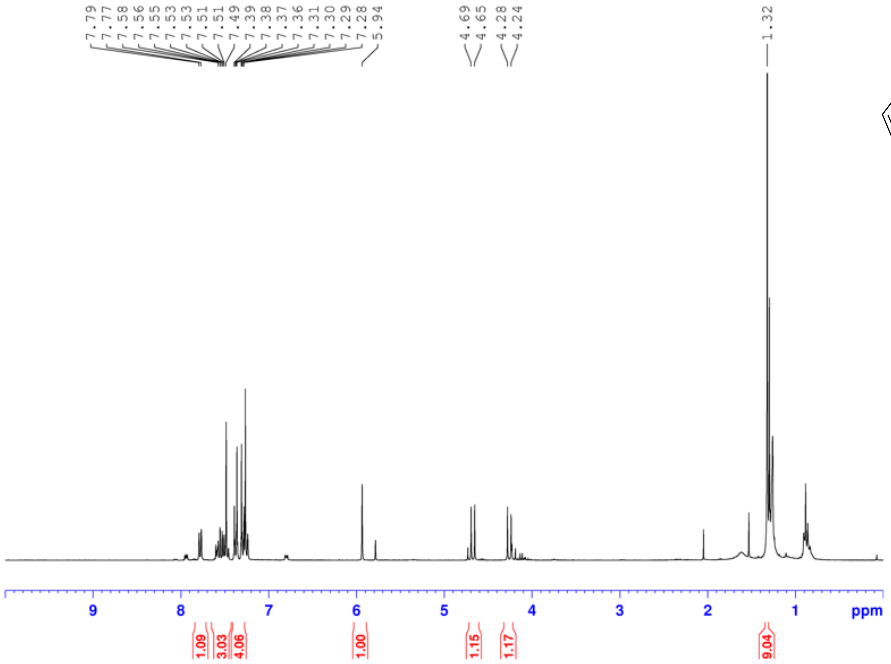
7.79 7.77 7.58 7.56 7.55 7.53 7.53 7.51 7.51 7.49 7.39 7.38 7.37 7.36 7.31 7.30 7.29 7.28 5.94
4.69 4.65 4.28 4.24
1.32
9 8 7 6 5 4 3 2 1 ppm
1.09 3.03 4.06 1.00 1.15 1.17 9.04

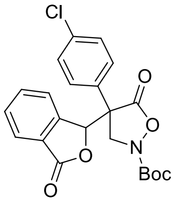
Cl
O
O
N
Boc
O
O

7g

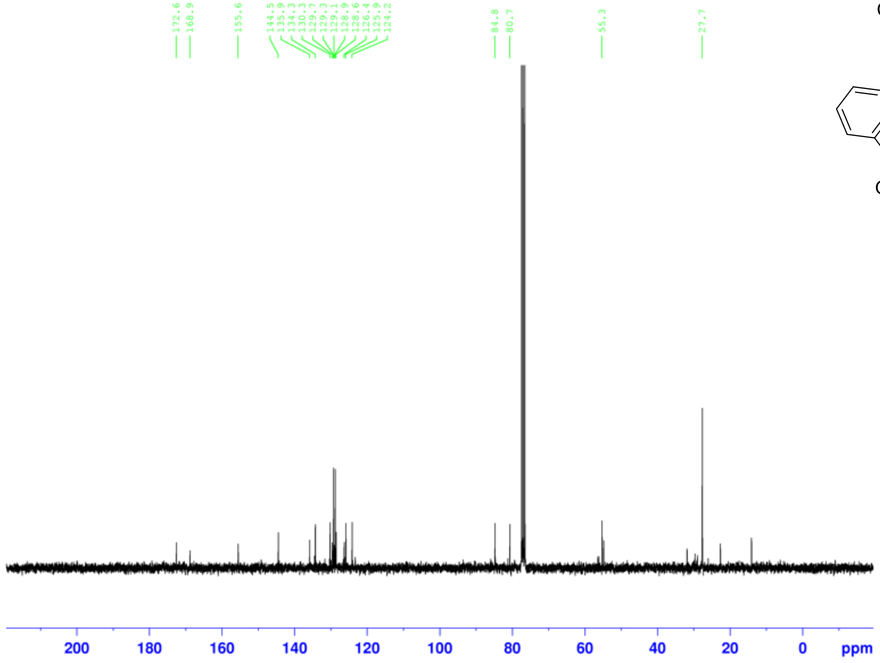
172.6 168.9 155.6 144.5 135.9 134.3 130.3 129.7 129.3 129.1 128.9 128.6 126.4 125.9 124.2 84.8 80.7 55.3 27.7
200 180 160 140 120 100 80 60 40 20 0 ppm

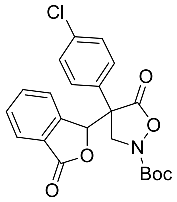
Cl
O
O
N
Boc
O
O

7g

Chromatogram

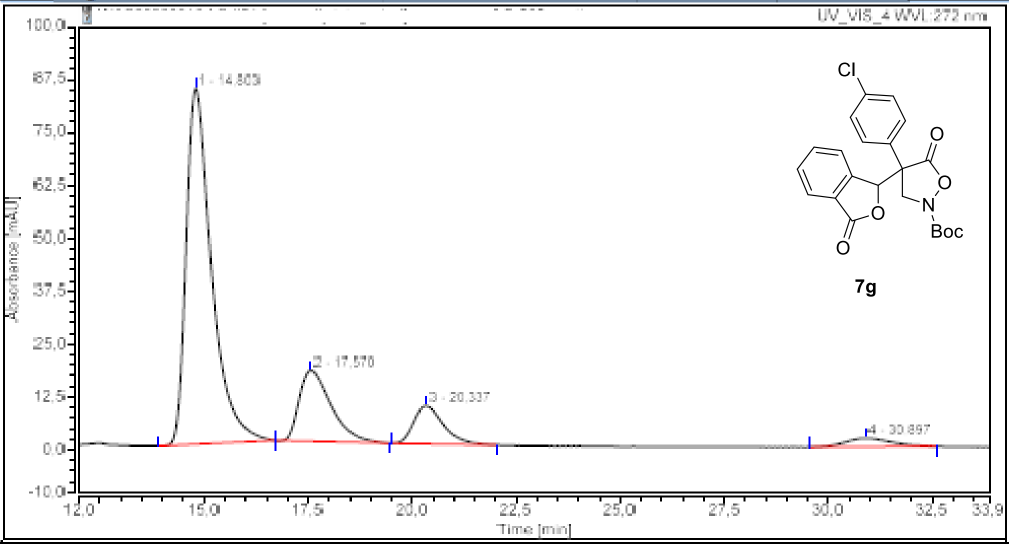

Integration Results

No.	Peak Name	Retention Time<br>min	Area<br>mAU*min	Height<br>mAU	Relative Area<br>%	Relative Height<br>%	Amount
n.a.	Peak 1	n.a.	n.a.	n.a.	n.a.	n.a.	n.a.
1		14,803	59,511	84,043	70,72	75,24	n.a.
2		17,570	14,807	16,699	17,60	14,95	n.a.
3		20,337	7,395	9,005	8,79	8,06	n.a.
4		30,897	2,435	1,950	2,89	1,75	n.a.
**Total:**			**84,147**	**111,697**	**100,00**	**100,00**	

Chromatogram

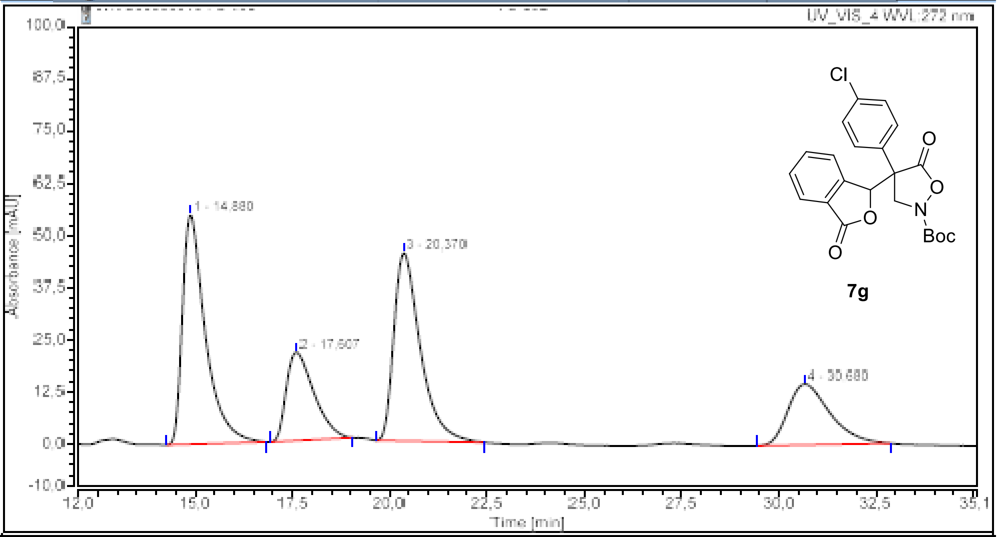

Integration Results

No.	Peak Name	Retention Time<br>min	Area<br>mAU*min	Height<br>mAU	Relative Area<br>%	Relative Height<br>%	Amount
n.a.	Peak 1	n.a.	n.a.	n.a.	n.a.	n.a.	n.a.
1		14,880	37,630	55,001	34,48	40,50	n.a.
2		17,607	17,426	21,171	15,96	15,59	n.a.
3		20,370	35,678	45,028	32,69	33,16	n.a.
4		30,680	18,418	14,608	16,87	10,76	n.a.
**Total:**			**109,152**	**135,808**	**100,00**	**100,00**	

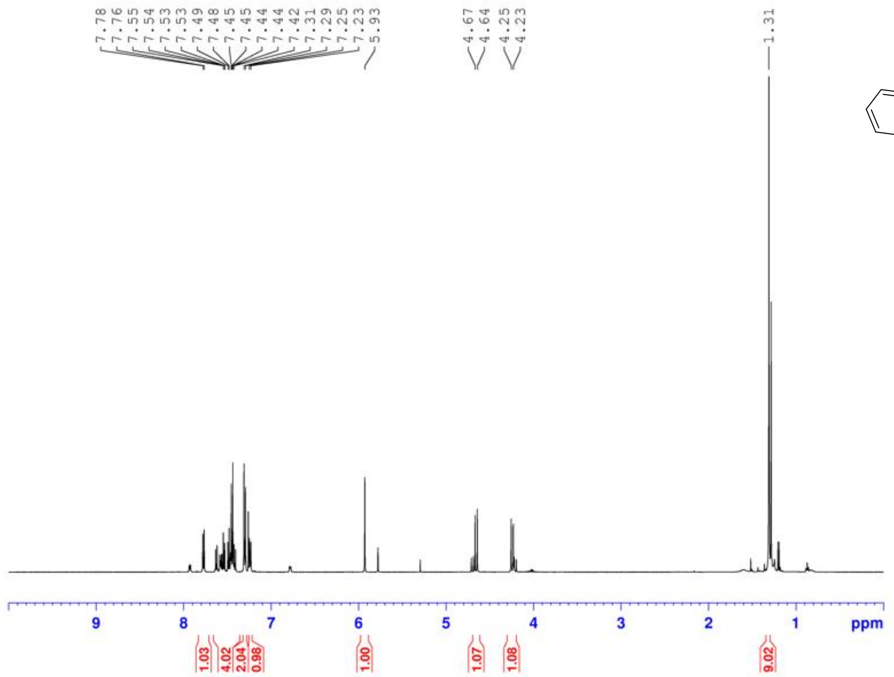
7.78 7.76 7.55 7.54 7.53 7.53 7.49 7.48 7.45 7.45 7.44 7.44 7.42 7.31 7.29 7.25 7.23 5.93
4.67 4.64 4.25 4.23
1.31
9 8 7 6 5 4 3 2 1 ppm
1.03 4.02 2.04 0.98 1.00 1.07 1.08 9.02

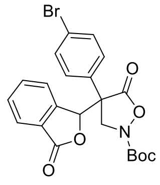
Br
O
O
N
Boc
O
O

7h

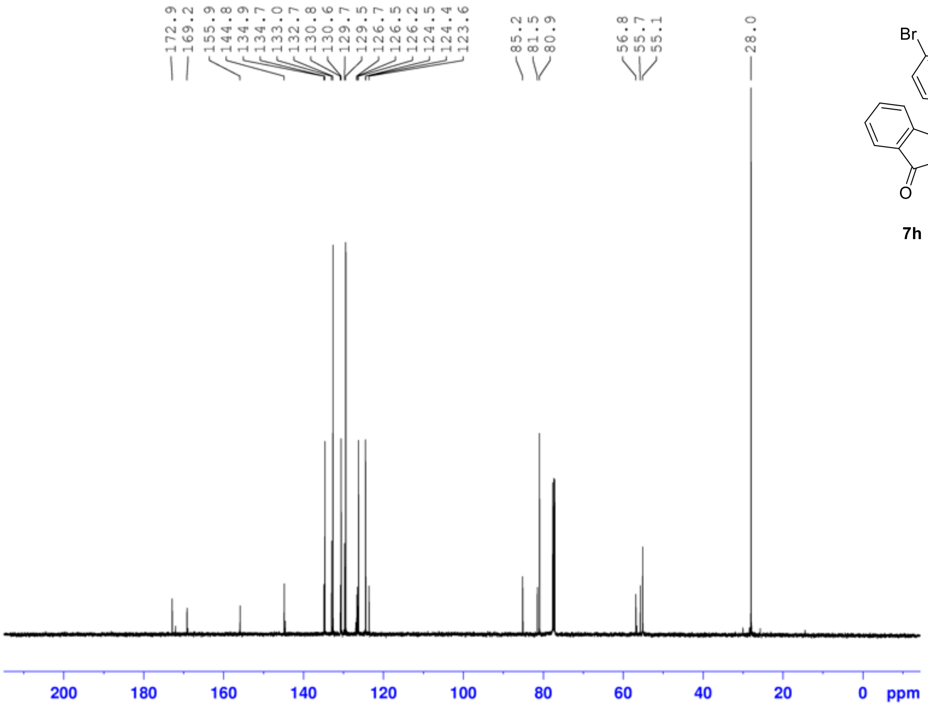
172.9 169.2 155.9 144.8 134.9 134.7 133.0 132.7 130.8 130.6 129.7 129.5 126.7 126.5 126.2 124.5 124.4 123.6
85.2 81.5 80.9
56.8 55.7 55.1
28.0
200 180 160 140 120 100 80 60 40 20 0 ppm

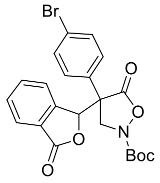
Br
O
O
N
Boc
O
O

7h

Chromatogram

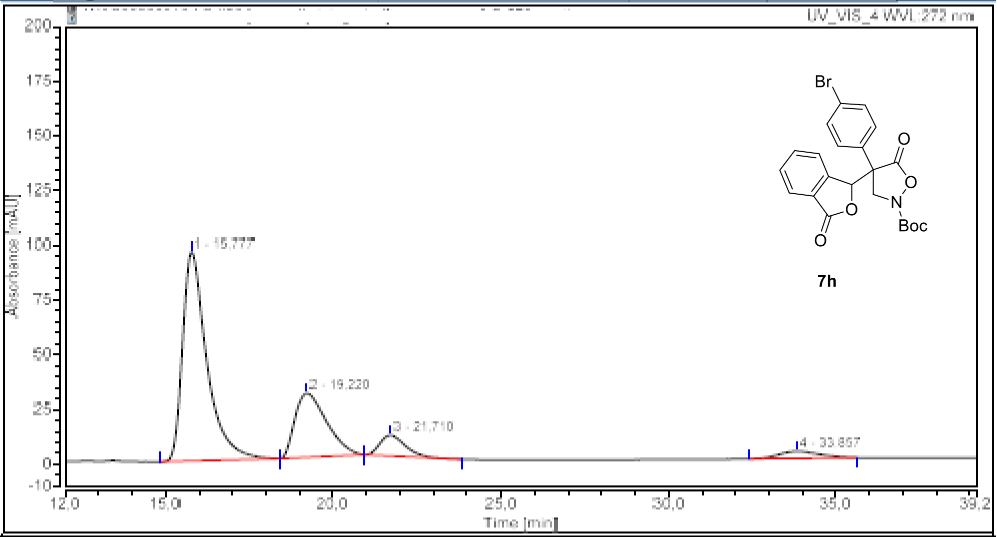

Integration Results

No.	Peak Name	Retention Time min	Area mAU*min	Height mAU	Relative Area %	Relative Height %	Amount
n.a.	Peak 1	n.a.	n.a.	n.a.	n.a.	n.a.	n.a.
1		15,777	80,247	95,444	64,27	69,51	n.a.
2		19,220	31,999	29,250	25,63	21,30	n.a.
3		21,710	8,171	9,431	6,54	6,87	n.a.
4		33,857	4,448	3,189	3,56	2,32	n.a.
Total:			124,865	137,314	100,00	100,00	

Chromatogram

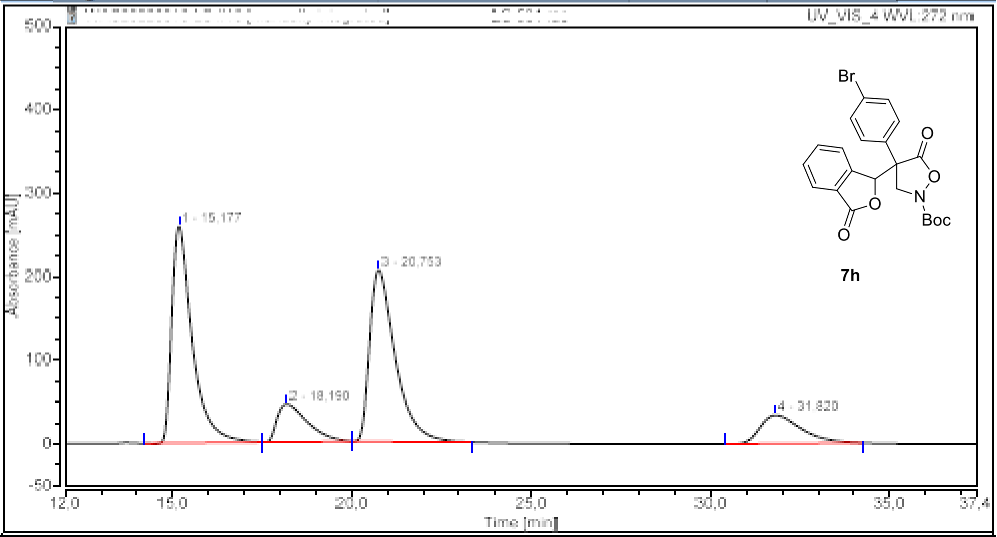

Integration Results

No.	Peak Name	Retention Time min	Area mAU*min	Height mAU	Relative Area %	Relative Height %	Amount
n.a.	Peak 1	n.a.	n.a.	n.a.	n.a.	n.a.	n.a.
1		15,177	171,926	259,330	39,79	47,72	n.a.
2		18,190	44,129	45,152	10,21	8,31	n.a.
3		20,753	170,788	204,861	39,52	37,70	n.a.
4		31,820	45,263	34,075	10,47	6,27	n.a.
Total:			432,106	543,418	100,00	100,00	

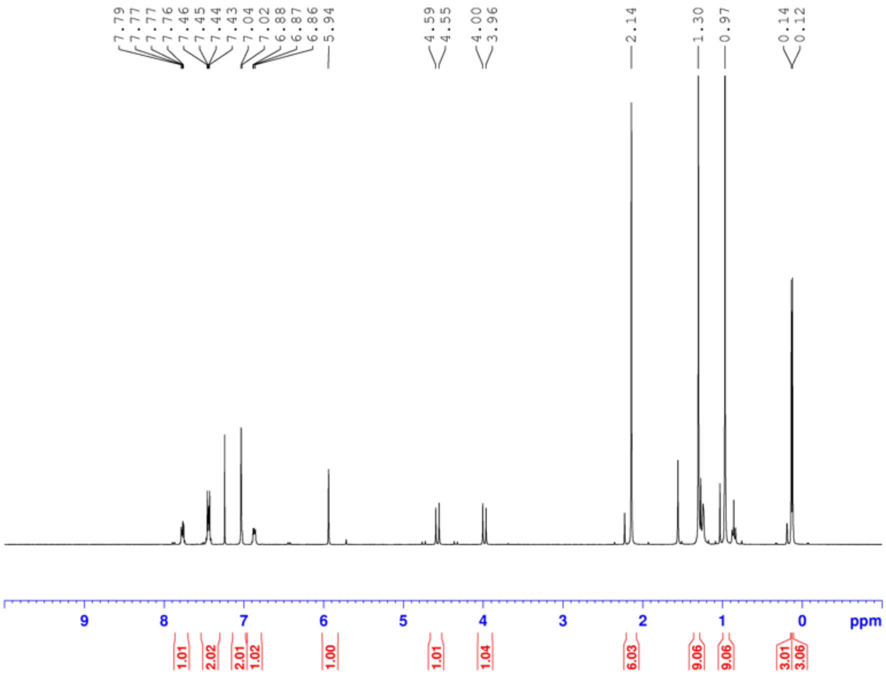
7.79 7.77 7.77 7.76 7.46 7.45 7.44 7.43 7.04 7.02 6.88 6.87 6.86 5.94 4.59 4.55 4.00 3.96 2.14 1.30 0.97 0.14 0.12
9 8 7 6 5 4 3 2 1 0 ppm
1.01 2.02 2.01 1.02 1.00 1.01 1.04 6.03 9.06 9.06 3.01 3.06

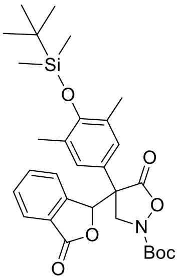
Si
O
O
O
O
N
O
O
Boc

7i

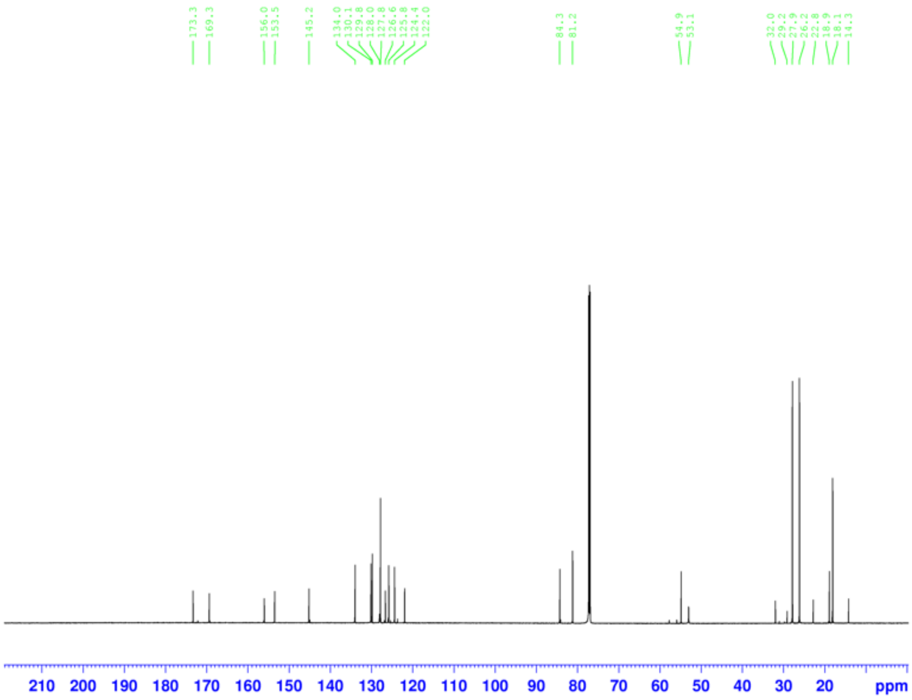
173.3 169.3 156.0 153.5 145.2 134.0 130.1 129.8 128.0 127.8 126.6 125.8 124.4 122.0 84.3 81.2 54.9 53.1 32.0 29.2 27.9 26.2 22.8 18.9 18.1 14.3
210 200 190 180 170 160 150 140 130 120 110 100 90 80 70 60 50 40 30 20 ppm

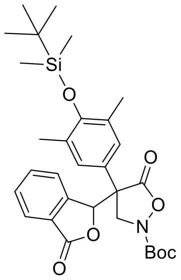
Si
O
O
O
O
N
O
O
Boc

7i

Chromatogram

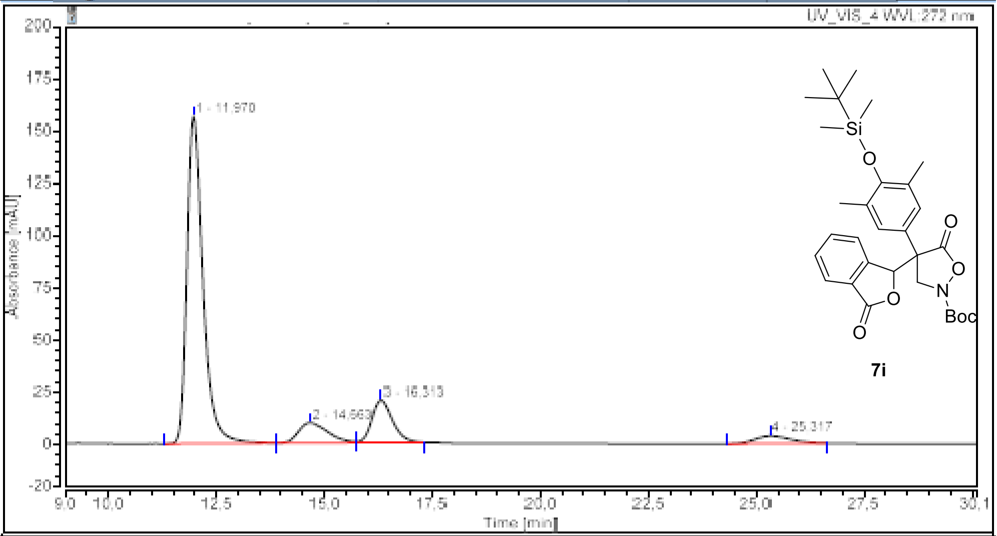

Integration Results

No.	Peak Name	Retention Time min	Area mAU*min	Height mAU	Relative Area %	Relative Height %	Amount
n.a.	Peak 1	n.a.	n.a.	n.a.	n.a.	n.a.	n.a.
1		11,970	67,578	156,621	75,25	82,40	n.a.
2		14,663	7,558	9,643	8,42	5,07	n.a.
3		16,313	11,204	20,200	12,48	10,63	n.a.
4		25,317	3,463	3,601	3,86	1,89	n.a.
**Total:**			**89,804**	**190,065**	**100,00**	**100,00**	

Chromatogram

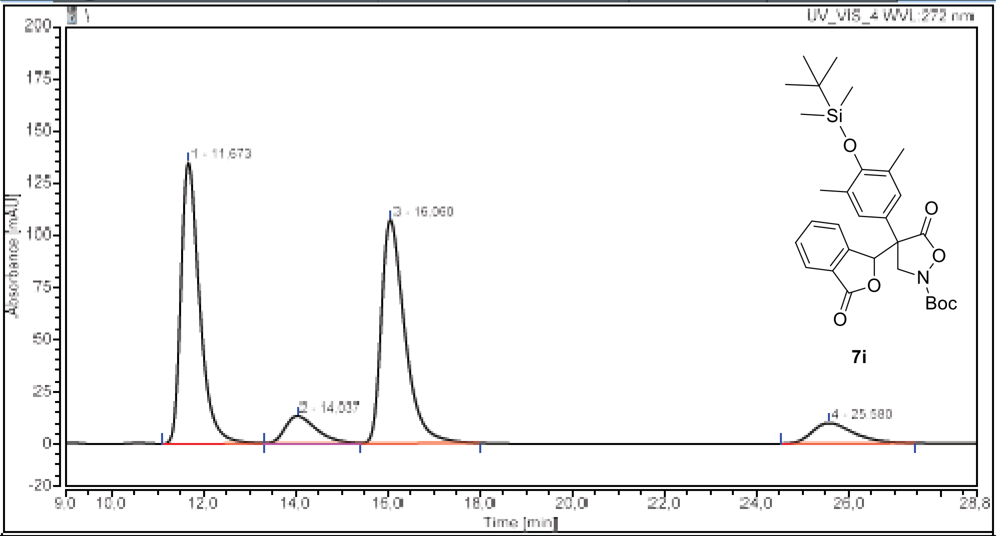

Integration Results

No.	Peak Name	Retention Time min	Area mAU*min	Height mAU	Relative Area %	Relative Height %	Amount
n.a.	Peak 1	n.a.	n.a.	n.a.	n.a.	n.a.	n.a.
1		11,673	63,594	134,742	43,10	50,95	n.a.
2		14,037	10,063	12,965	6,82	4,90	n.a.
3		16,060	63,595	106,952	43,10	40,44	n.a.
4		25,580	10,300	9,783	6,98	3,70	n.a.
**Total:**			**147,551**	**264,442**	**100,00**	**100,00**	

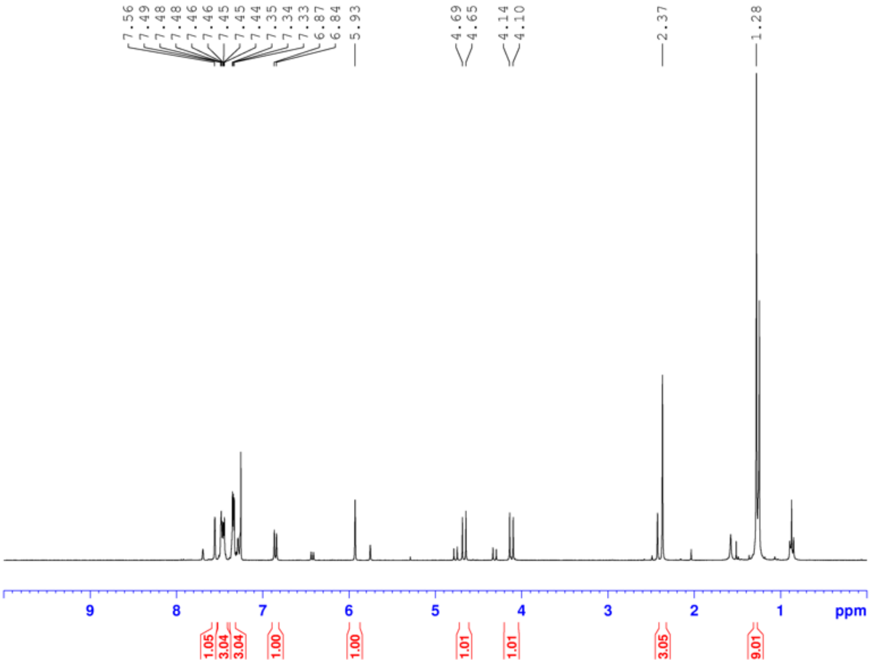
7.56
7.49
7.48
7.48
7.46
7.46
7.45
7.45
7.44
7.35
7.34
7.33
6.87
6.84
5.93
4.69
4.65
4.14
4.10
2.37
1.28
9
8
7
6
5
4
3
2
1
ppm
1.05
3.04
3.04
1.00
1.00
1.01
1.01
3.05
9.01

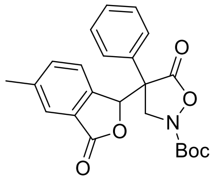
O
O
N
Boc
O
O

7j

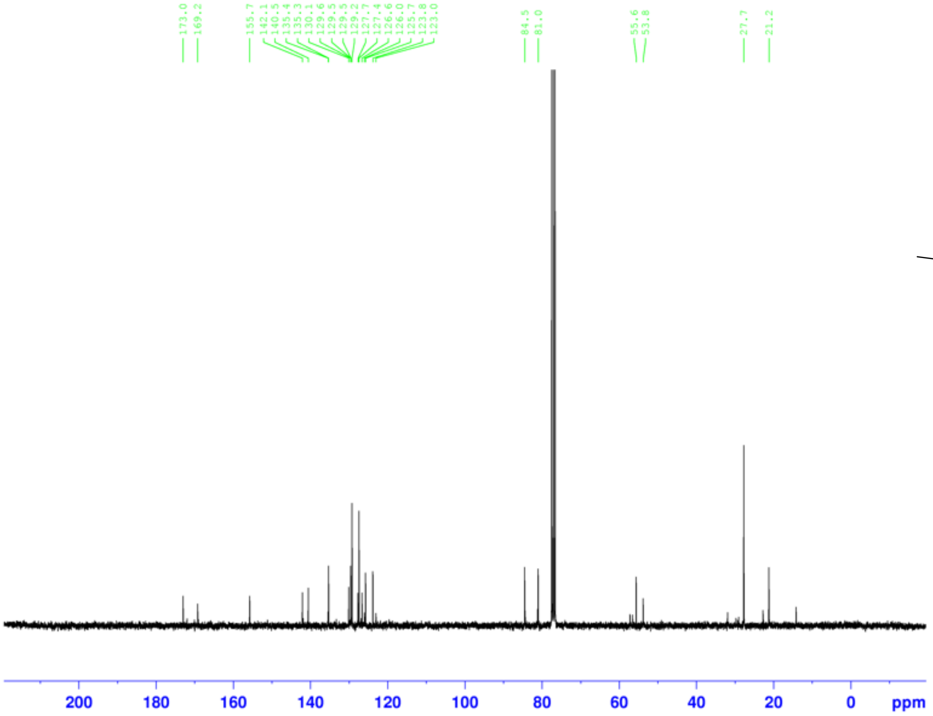
173.0
169.2
155.7
142.1
140.5
135.4
135.3
130.1
129.6
129.5
129.5
129.2
127.7
127.4
126.6
126.0
125.7
123.8
123.0
84.5
81.0
55.6
53.8
27.7
21.2
200
180
160
140
120
100
80
60
40
20
0
ppm

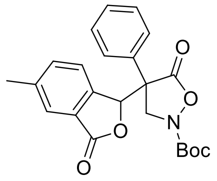
O
O
N
Boc
O
O

7j

Chromatogram

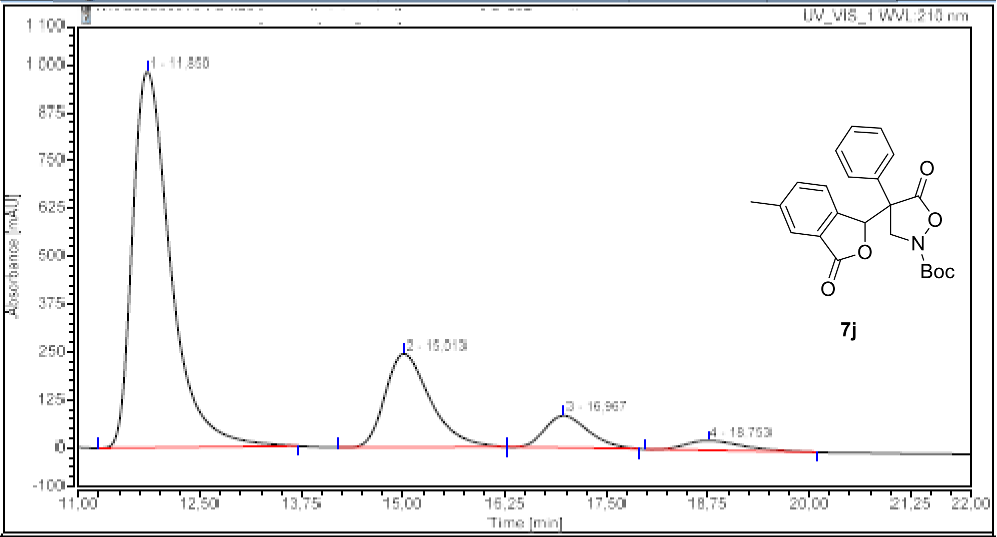

Integration Results

No.	Peak Name	Retention Time min	Area mAU*min	Height mAU	Relative Area %	Relative Height %	Amount
n.a.	Peak 1	n.a.	n.a.	n.a.	n.a.	n.a.	n.a.
1		11,850	534,859	981,978	70,02	73,59	n.a.
2		15,013	156,921	243,695	20,54	18,26	n.a.
3		16,967	51,637	83,255	6,76	6,24	n.a.
4		18,753	20,476	25,402	2,68	1,90	n.a.
Total:			763,892	1334,330	100,00	100,00	

Chromatogram

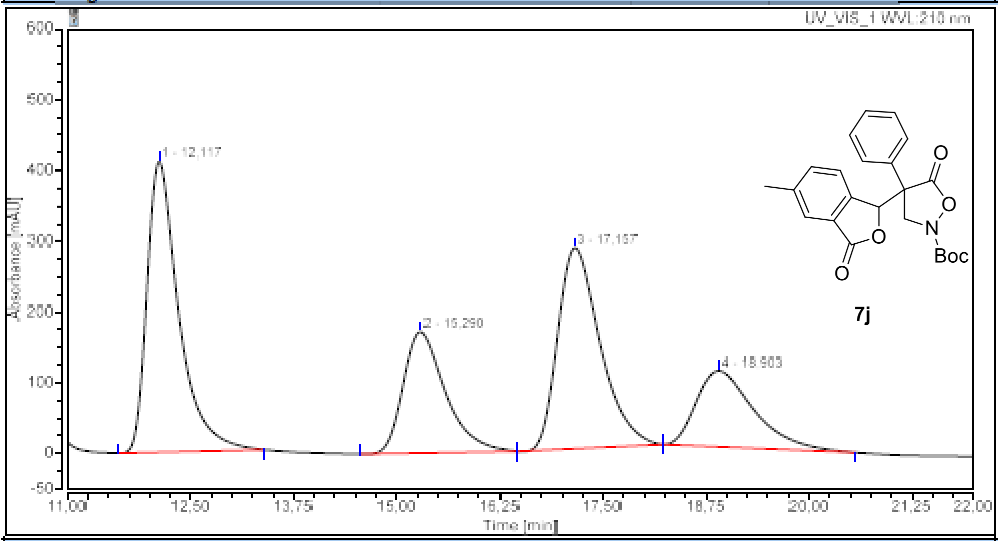

Integration Results

No.	Peak Name	Retention Time min	Area mAU*min	Height mAU	Relative Area %	Relative Height %	Amount
n.a.	Peak 1	n.a.	n.a.	n.a.	n.a.	n.a.	n.a.
1		12,117	184,138	410,287	33,94	42,16	n.a.
2		15,290	101,936	171,401	18,79	17,61	n.a.
3		17,157	169,405	284,173	31,22	29,20	n.a.
4		18,903	87,055	107,395	16,05	11,03	n.a.
Total:			542,534	973,255	100,00	100,00	

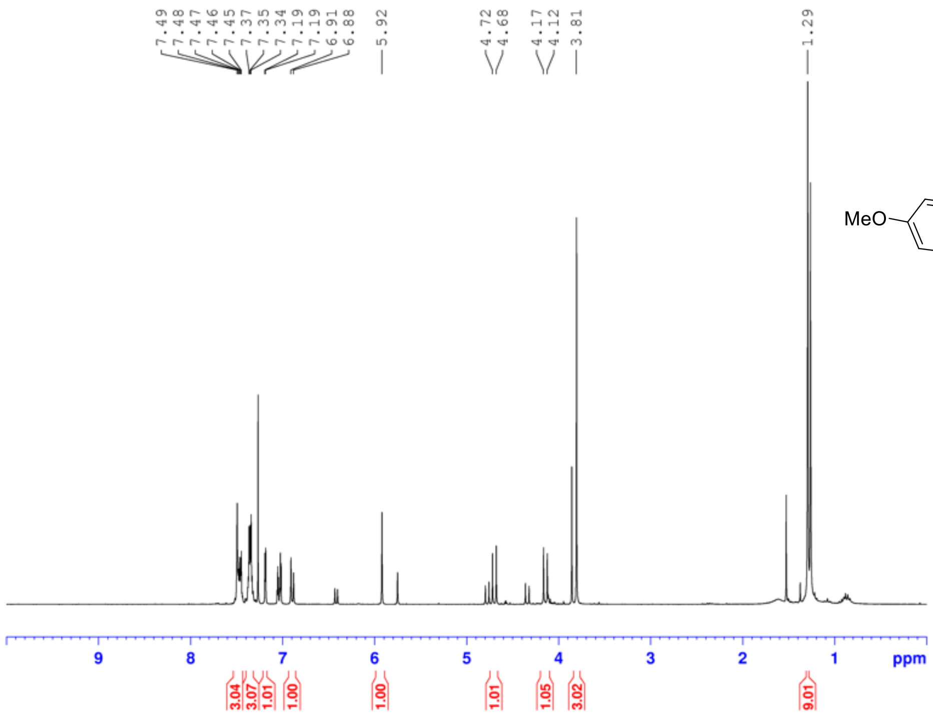
7.49
7.48
7.47
7.46
7.45
7.37
7.35
7.34
7.19
7.19
6.91
6.88
5.92
4.72
4.68
4.17
4.12
3.81
1.29
9
8
7
6
5
4
3
2
1
ppm
3.04
3.07
1.01
1.00
1.00
1.01
1.05
3.02
9.01

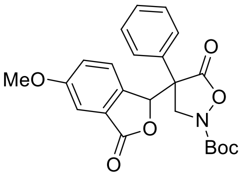
MeO
O
O
N
Boc
O
O

7k

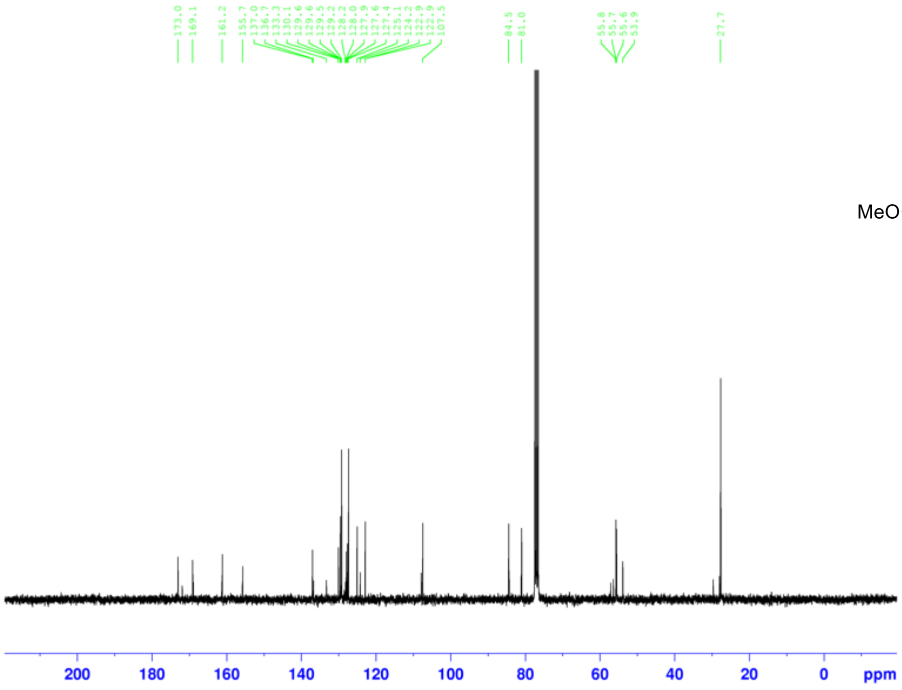
200
180
160
140
120
100
80
60
40
20
0
ppm

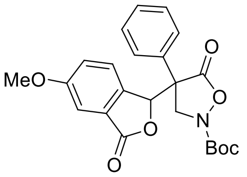
MeO
O
O
N
Boc
O
O

7k

Chromatogram

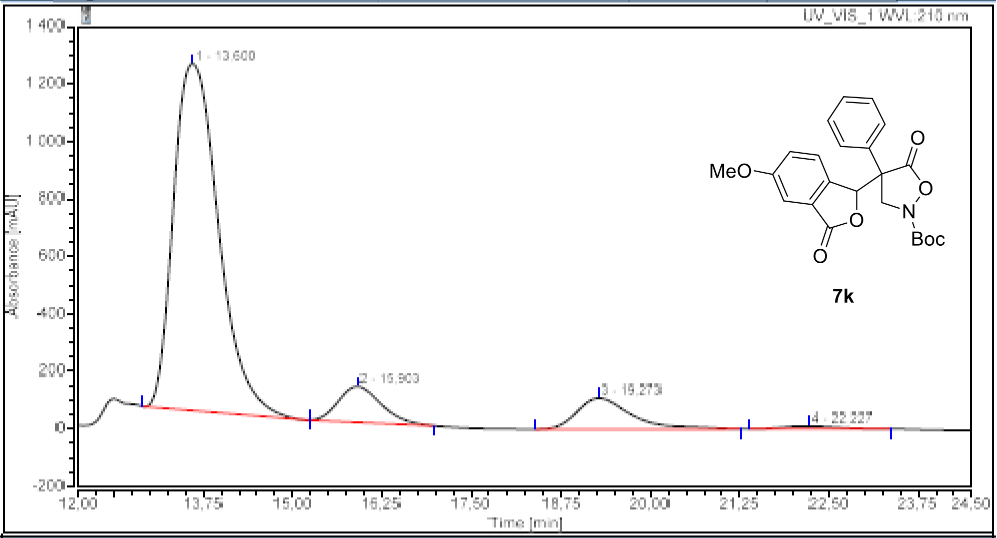

Integration Results

No.	Peak Name	Retention Time min	Area mAU*min	Height mAU	Relative Area %	Relative Height %	Amount
n.a.	Peak 1	n.a.	n.a.	n.a.	n.a.	n.a.	n.a.
1		13,600	885,900	1207,210	82,36	83,30	n.a.
2		15,903	86,310	124,459	8,02	8,59	n.a.
3		19,273	94,947	108,003	8,83	7,45	n.a.
4		22,227	8,431	9,612	0,78	0,66	n.a.
**Total:**			**1075,589**	**1449,284**	**100,00**	**100,00**	

Chromatogram

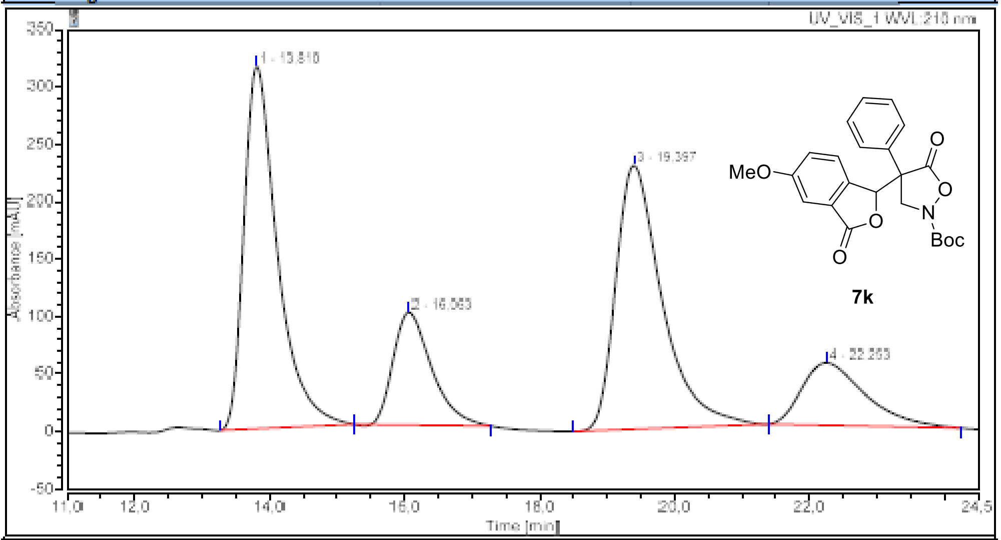

Integration Results

No.	Peak Name	Retention Time min	Area mAU*min	Height mAU	Relative Area %	Relative Height %	Amount
n.a.	Peak 1	n.a.	n.a.	n.a.	n.a.	n.a.	n.a.
1		13,810	181,057	315,095	37,25	45,27	n.a.
2		16,063	64,229	97,664	13,21	14,03	n.a.
3		19,397	182,187	229,261	37,48	32,94	n.a.
4		22,253	58,579	53,970	12,05	7,75	n.a.
**Total:**			**486,051**	**695,989**	**100,00**	**100,00**	

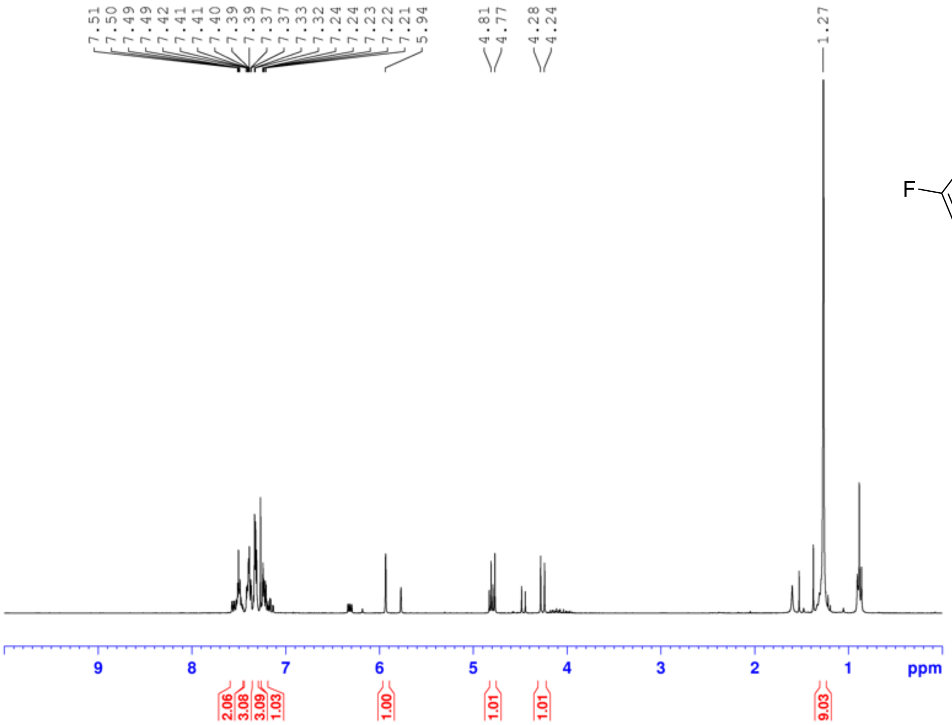
7.51
7.50
7.49
7.49
7.42
7.41
7.41
7.40
7.39
7.39
7.37
7.37
7.33
7.32
7.24
7.24
7.23
7.22
7.21
5.94
4.81
4.77
4.28
4.24
1.27
9
8
7
6
5
4
3
2
1
ppm
2.06
3.08
3.09
1.03
1.00
1.01
1.01
9.03

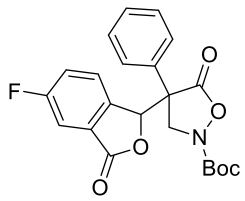
F
O
O
N
O
Boc
O

7l

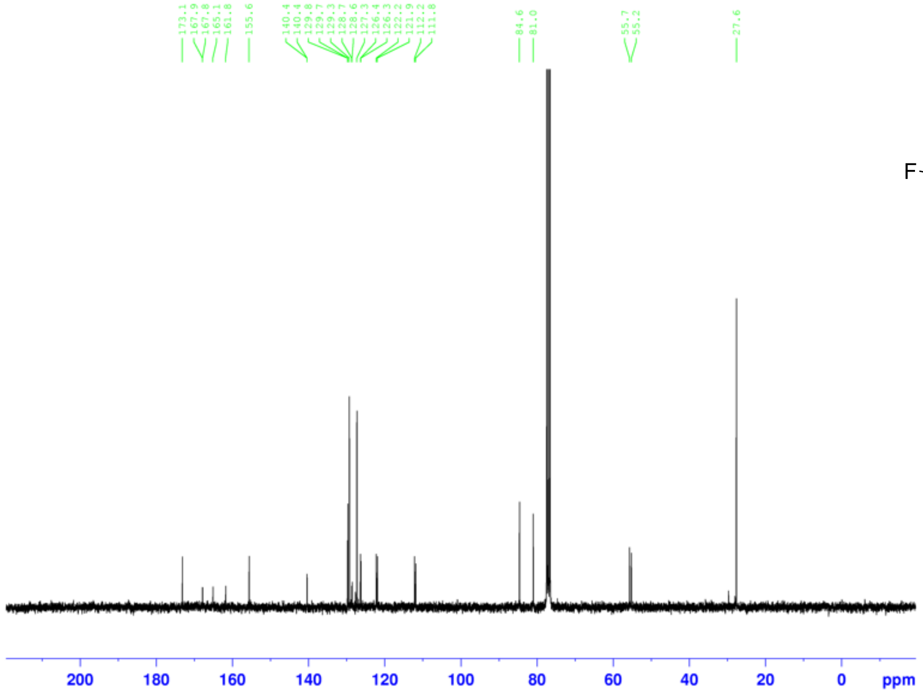
173.1
167.9
167.8
165.1
161.8
155.6
140.4
140.4
129.8
129.7
129.3
128.7
128.6
127.3
126.4
126.3
122.2
121.9
112.2
111.8
84.6
81.0
55.7
55.2
27.6
200
180
160
140
120
100
80
60
40
20
0
ppm

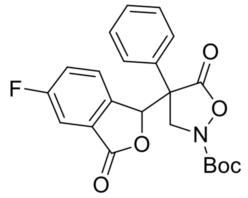
F
O
O
N
O
Boc
O

7l

Chromatogram

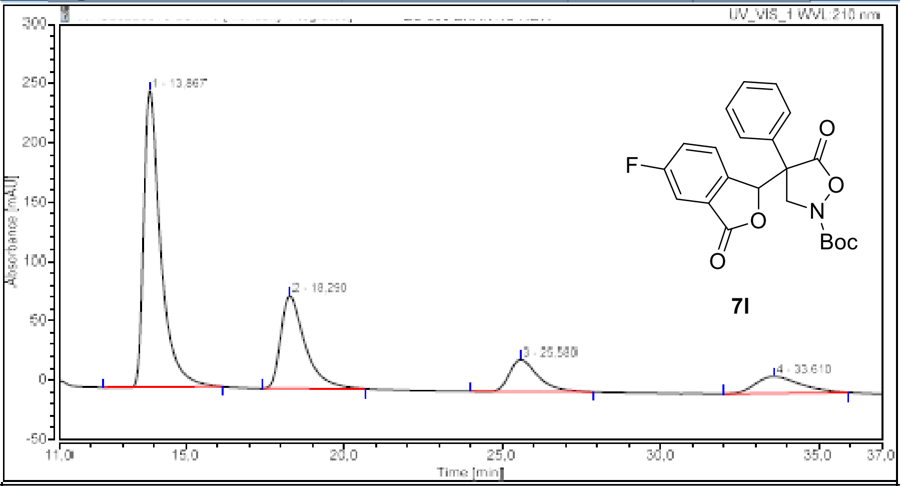

Integration Results

No.	Peak Name	Retention Time min	Area mAU*min	Height mAU	Relative Area %	Relative Height %	Amount
n.a.	Peak 1	n.a.	n.a.	n.a.	n.a.	n.a.	n.a.
1		13,867	155,351	250,154	56,24	67,73	n.a.
2		18,290	69,972	78,228	25,33	21,18	n.a.
3		25,580	27,745	26,806	10,04	7,26	n.a.
4		33,610	23,140	14,157	8,38	3,83	n.a.
**Total:**			**276,208**	**369,346**	**100,00**	**100,00**	

Chromatogram

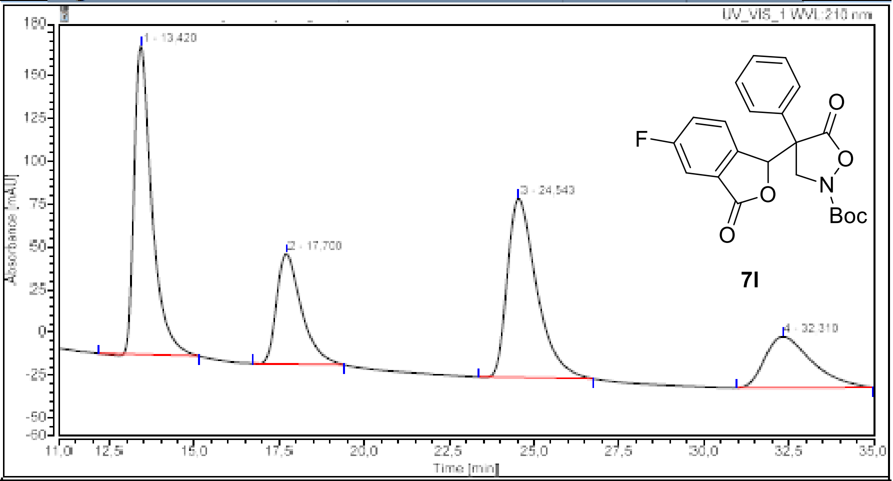

Integration Results

No.	Peak Name	Retention Time min	Area mAU*min	Height mAU	Relative Area %	Relative Height %	Amount
n.a.	Peak 1	n.a.	n.a.	n.a.	n.a.	n.a.	n.a.
1		13,420	108,824	180,108	34,51	47,58	n.a.
2		17,700	54,540	64,309	17,29	16,99	n.a.
3		24,543	104,562	104,469	33,16	27,60	n.a.
4		32,310	47,439	29,685	15,04	7,84	n.a.
**Total:**			**315,364**	**378,572**	**100,00**	**100,00**	

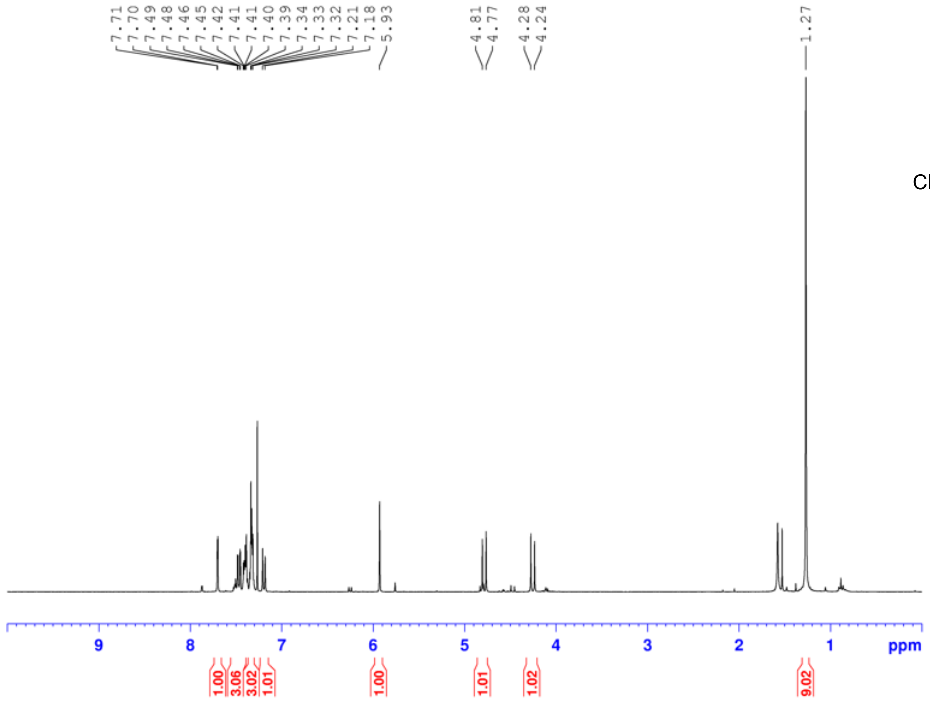
7.71 7.70 7.49 7.48 7.46 7.45 7.42 7.41 7.41 7.40 7.39 7.34 7.33 7.32 7.21 7.18 5.93
4.81 4.77
4.28 4.24
1.27
9 8 7 6 5 4 3 2 1 ppm
1.00 3.06 3.02 1.01
1.00
1.01
1.02
9.02

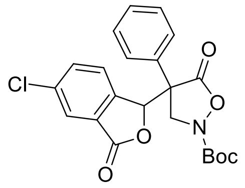
Cl
O
O
N
O
Boc
O

7m

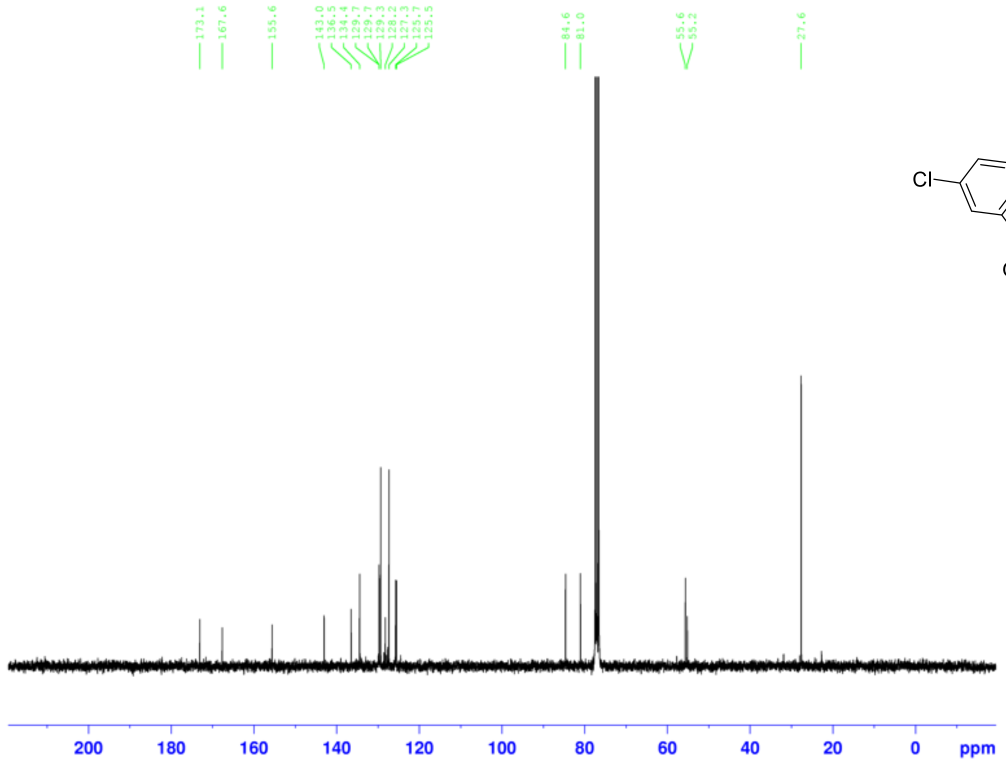
173.1 167.6 155.6 143.0 136.5 134.4 129.7 129.7 129.3 128.2 127.3 125.7 125.5 84.6 81.0 55.6 55.2 27.6
200 180 160 140 120 100 80 60 40 20 0 ppm

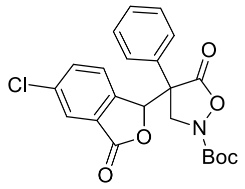
Cl
O
O
N
O
Boc
O

7m

Chromatogram

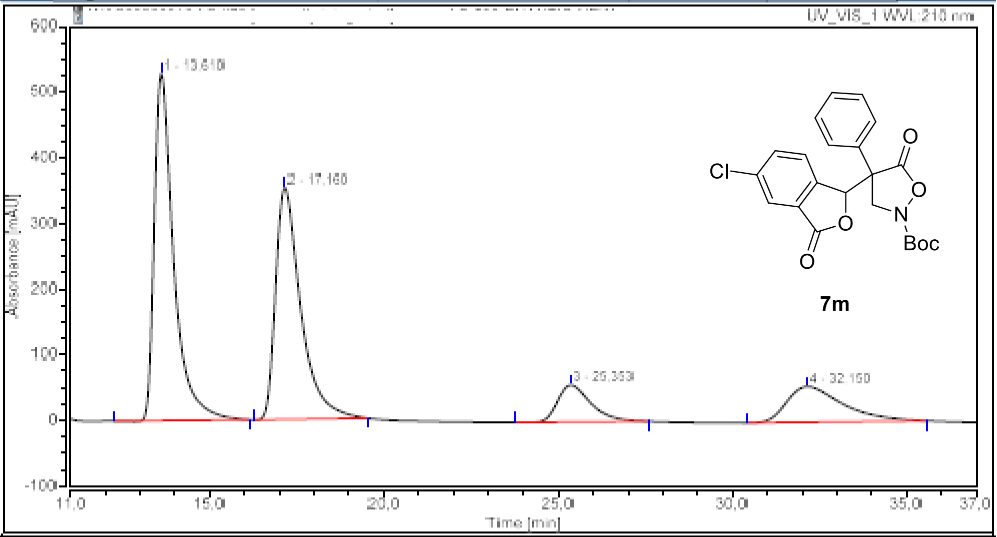

Integration Results

No.	Peak Name	Retention Time min	Area mAU*min	Height mAU	Relative Area %	Relative Height %	Amount
n.a.	Peak 1	n.a.	n.a.	n.a.	n.a.	n.a.	n.a.
1		13,610	341,404	528,606	42,65	53,30	n.a.
2		17,160	295,864	352,668	36,96	35,56	n.a.
3		25,353	62,123	56,114	7,76	5,66	n.a.
4		32,150	101,093	54,311	12,63	5,48	n.a.
Total:			800,483	991,699	100,00	100,00	

Chromatogram

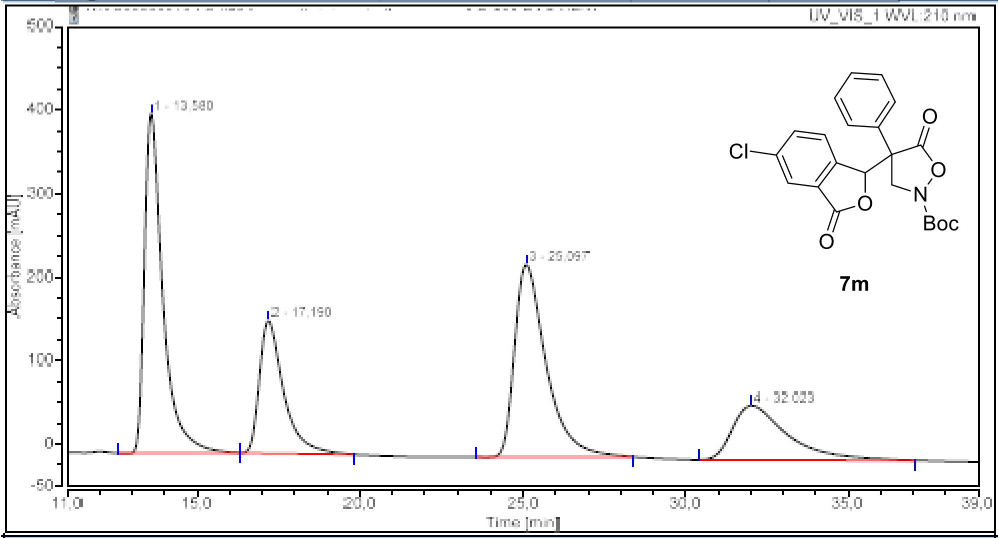

Integration Results

No.	Peak Name	Retention Time min	Area mAU*min	Height mAU	Relative Area %	Relative Height %	Amount
n.a.	Peak 1	n.a.	n.a.	n.a.	n.a.	n.a.	n.a.
1		13,580	267,797	406,178	33,79	47,18	n.a.
2		17,190	137,391	158,879	17,33	18,46	n.a.
3		25,097	259,549	230,731	32,74	26,80	n.a.
4		32,023	127,900	65,045	16,14	7,56	n.a.
Total:			792,636	860,833	100,00	100,00	

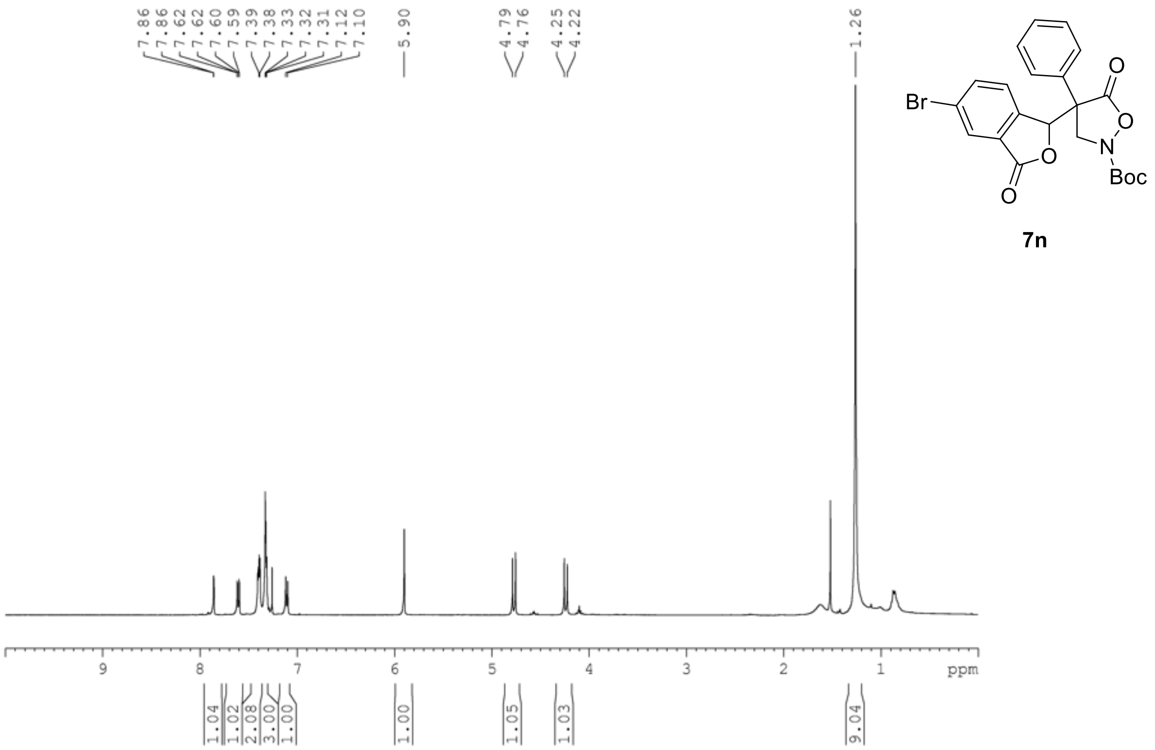
7.86
7.86
7.62
7.62
7.60
7.59
7.39
7.38
7.33
7.32
7.31
7.12
7.10
5.90
4.79
4.76
4.25
4.22
1.26
Br
O
O
N
O
Boc
O
O
7n
9
8
7
6
5
4
3
2
1
ppm
1.04
1.02
2.08
3.00
1.00
1.00
1.05
1.03
9.04

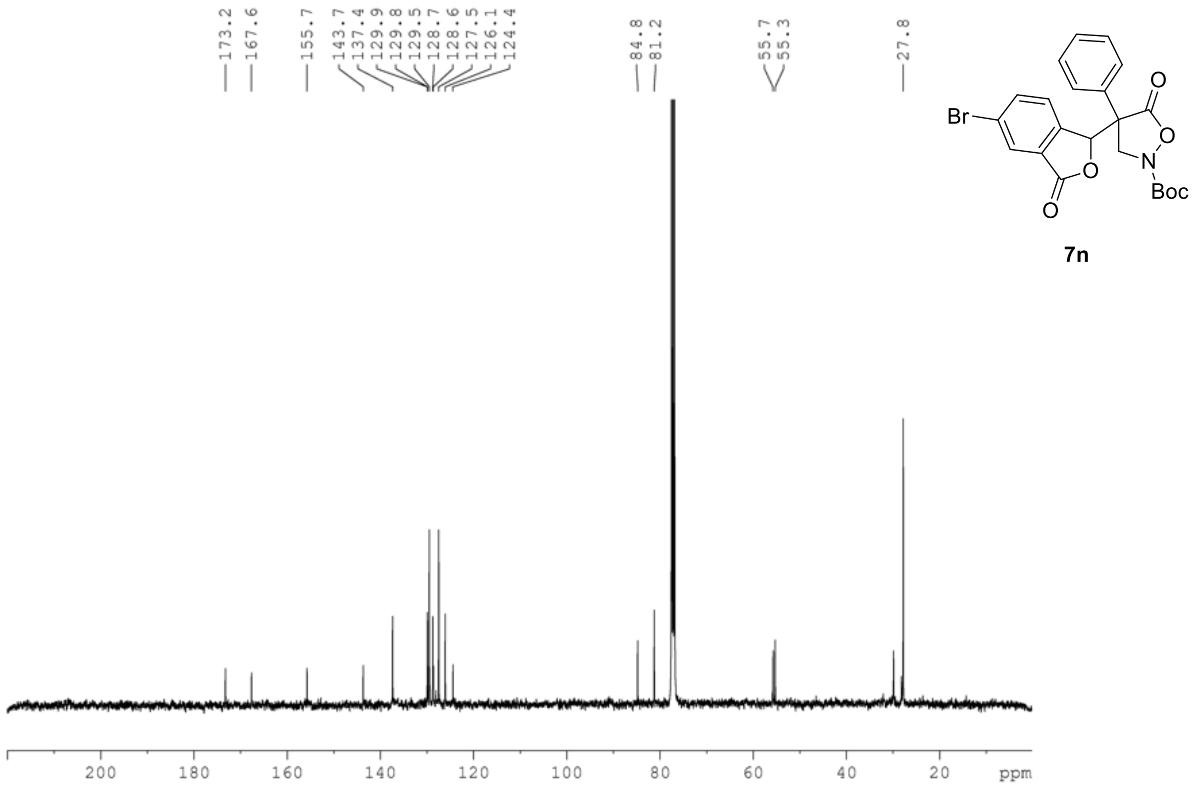
173.2
167.6
155.7
143.7
137.4
129.9
129.8
129.5
128.7
128.6
127.5
126.1
124.4
84.8
81.2
55.7
55.3
27.8
Br
O
O
N
O
Boc
O
O
7n
200
180
160
140
120
100
80
60
40
20
ppm

Chromatogram

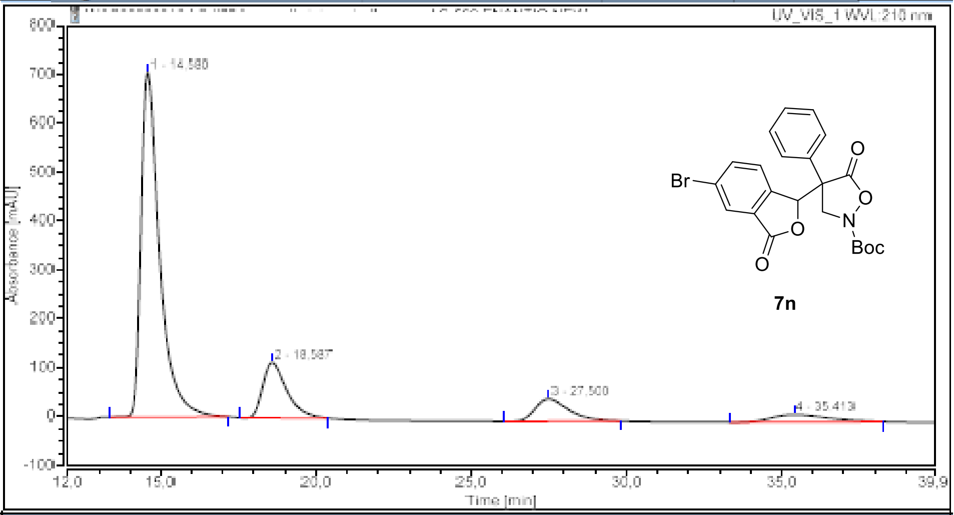

Integration Results

No.	Peak Name	Retention Time min	Area mAU*min	Height mAU	Relative Area %	Relative Height %	Amount
n.a.	Peak 1	n.a.	n.a.	n.a.	n.a.	n.a.	n.a.
1		14,580	491,249	704,819	72,14	80,22	n.a.
2		18,587	100,869	112,908	14,81	12,85	n.a.
3		27,500	57,507	45,692	8,45	5,20	n.a.
4		35,413	31,326	15,144	4,60	1,72	n.a.
Total:			680,951	878,563	100,00	100,00	

Chromatogram

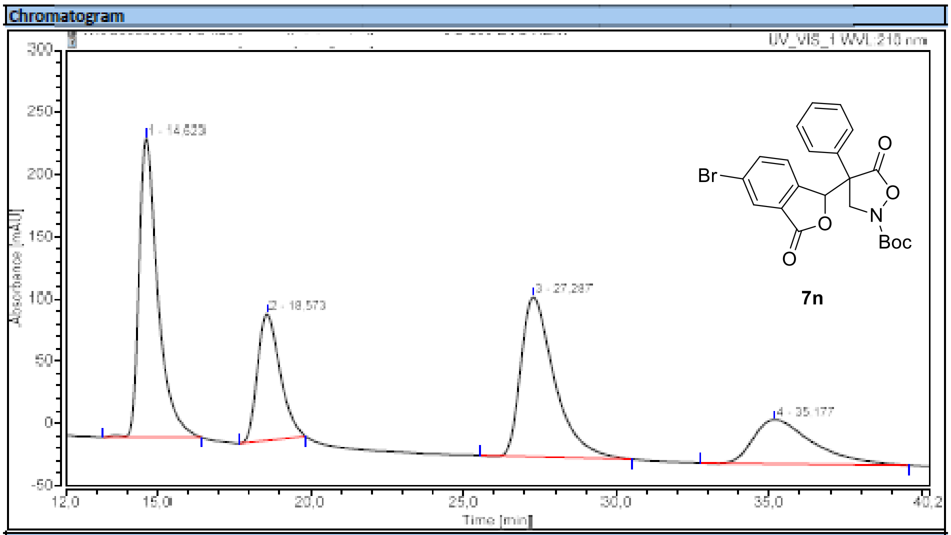

Integration Results

No.	Peak Name	Retention Time min	Area mAU*min	Height mAU	Relative Area %	Relative Height %	Amount
n.a.	Peak 1	n.a.	n.a.	n.a.	n.a.	n.a.	n.a.
1		14,623	172,290	240,707	34,27	47,53	n.a.
2		18,573	87,379	101,739	17,38	20,09	n.a.
3		27,287	163,459	128,415	32,51	25,36	n.a.
4		35,177	79,624	35,538	15,84	7,02	n.a.
Total:			502,752	506,398	100,00	100,00	

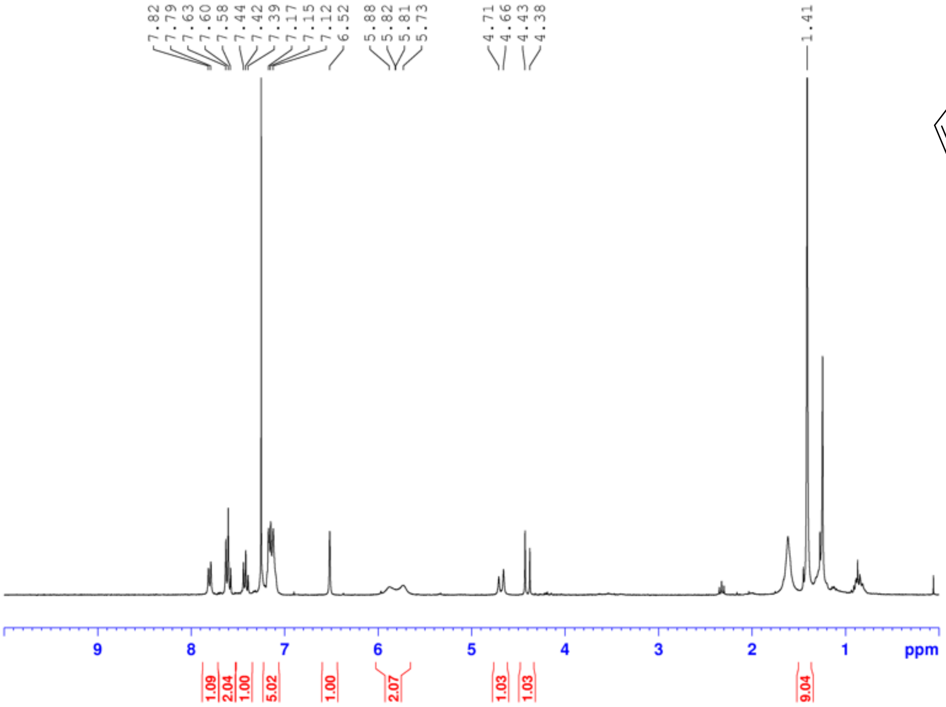
7.82 7.79 7.63 7.60 7.58 7.44 7.42 7.39 7.17 7.15 7.12 6.52 5.88 5.82 5.81 5.73 4.71 4.66 4.43 4.38 1.41
9 8 7 6 5 4 3 2 1 ppm
1.09 2.04 1.00 5.02 1.00 2.07 1.03 1.03 9.04

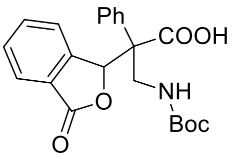
Ph
COOH
O
NH
Boc
O

8

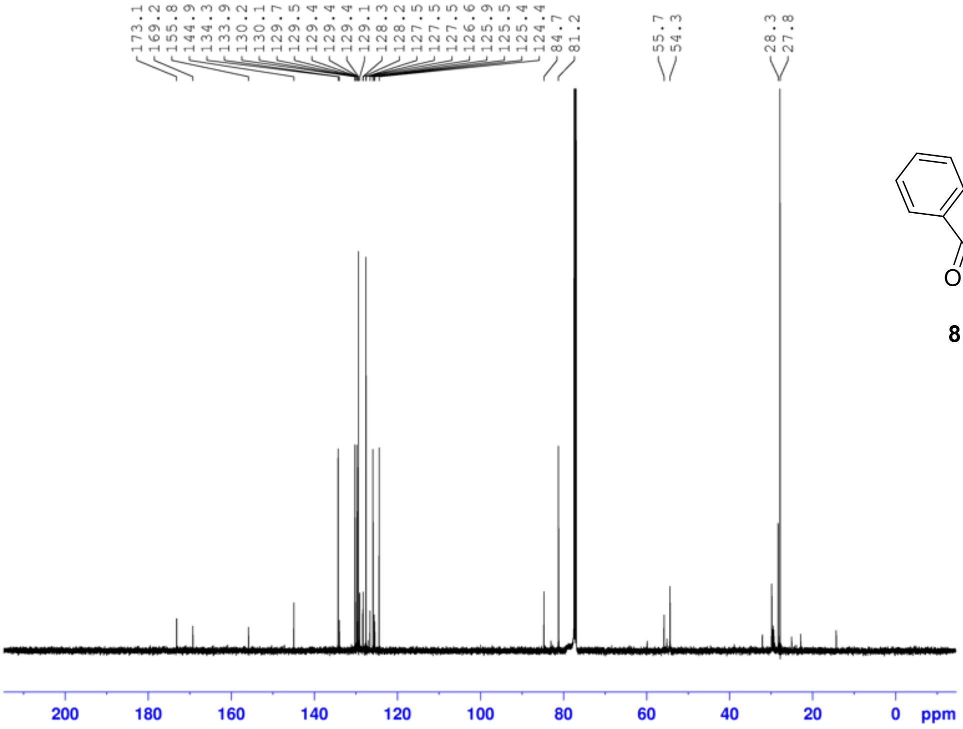
173.1 169.2 155.8 144.9 134.3 133.9 130.2 130.1 129.7 129.5 129.4 129.4 129.4 129.1 128.3 128.2 127.5 127.5 126.6 125.9 125.5 125.4 124.4 84.7 81.2 55.7 54.3 28.3 27.8
200 180 160 140 120 100 80 60 40 20 0 ppm

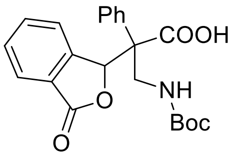
Ph
COOH
O
NH
Boc
O

8

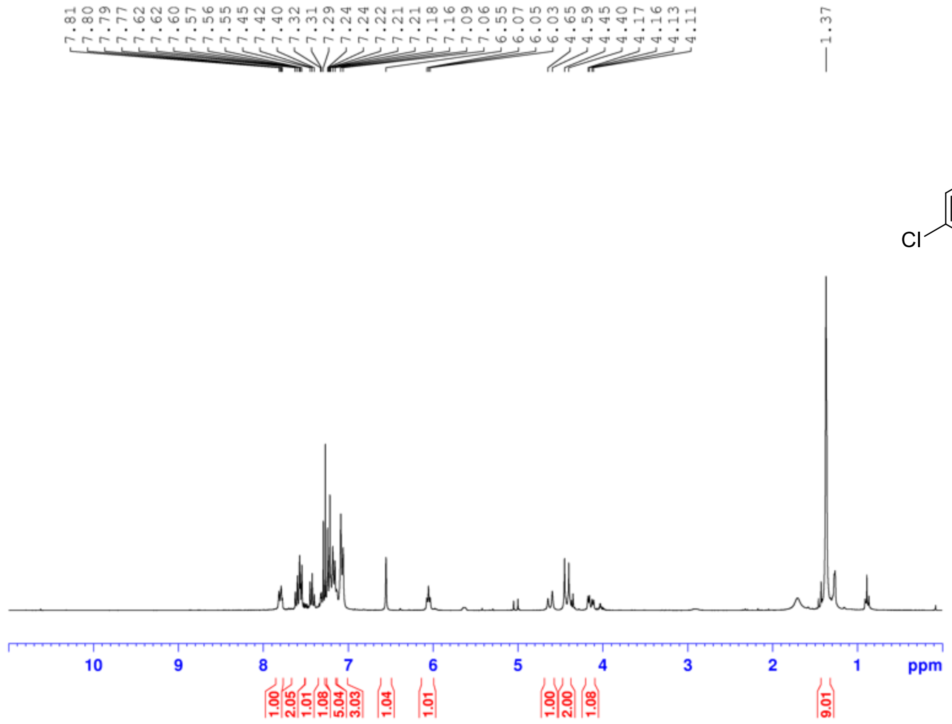

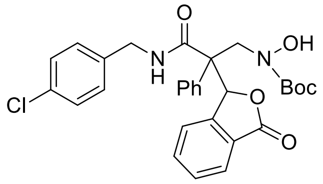

9

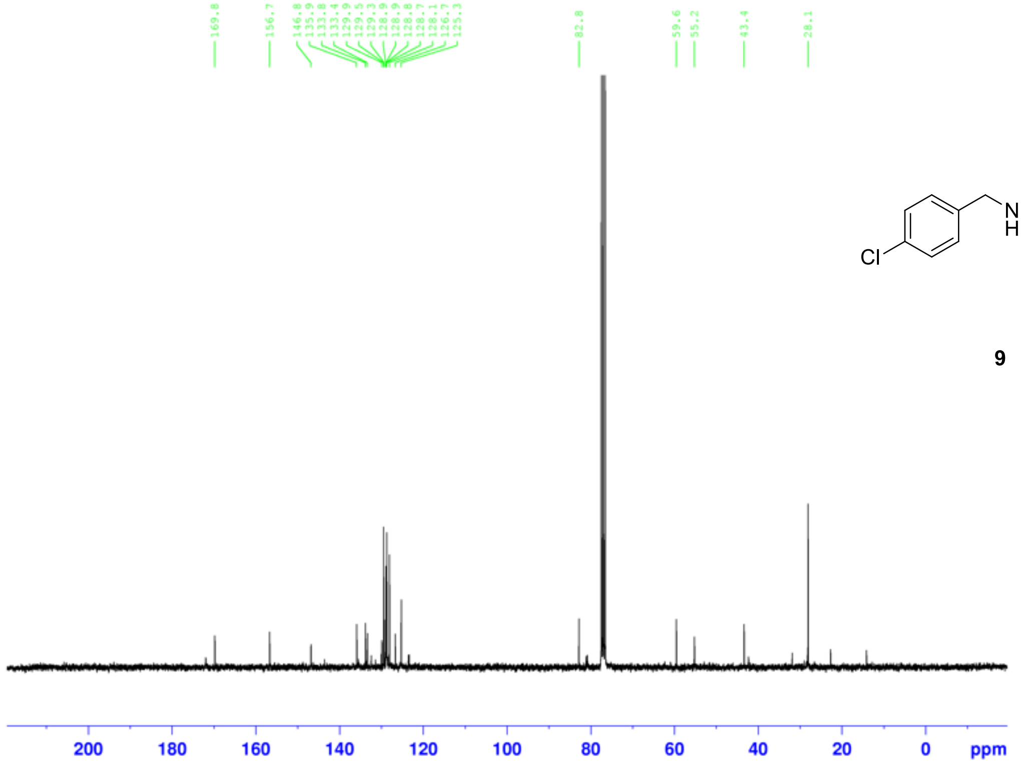

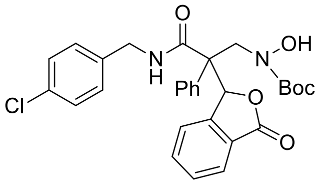

9
